# Bibliometric Study of the Comorbidity of Pain and Depression Research

**DOI:** 10.1155/2019/1657498

**Published:** 2019-10-23

**Authors:** Xue-Qiang Wang, Meng-Si Peng, Lin-Man Weng, Yi-Li Zheng, Zhi-Jie Zhang, Pei-Jie Chen

**Affiliations:** ^1^Department of Sport Rehabilitation, Shanghai University of Sport, 399 Changhai RD, Shanghai 200438, China; ^2^Department of Rehabilitation Medicine, Shanghai Shangti Orthopaedic Hospital, 188 Hengren RD, Shanghai 200438, China; ^3^Luoyang Orthopedic Hospital of Henan Province, 82 Qimingnan RD, Luoyang 471000, China

## Abstract

**Background:**

Comorbid pain and depression occur with high prevalence in clinical observations, and published academic journals about them have been increasing in number over time. However, few studies used the bibliometric method to analyze the general aspects of scientific researches on the comorbidity of pain and depression. The aim of this study is to systematically provide global scientific research in the comorbidity of pain and depression from 1980 to 2018.

**Methods:**

The published papers were searched between 1980 and 2018 in Web of Science. Publications related to comorbid pain and depression research were included. The language was restricted to English, and no species limitations were specified.

**Results:**

A total of 2,519 papers met the inclusion criteria in our study. The results revealed that the publications had a significant growth over time in the comorbidity of pain and depression research (*P* < 0.001) by linear regression analyses. The United States had the largest number of publications and citations and the highest value of *H*-index. According to subject categories of Web of Science, research areas of the 2,519 papers mainly focused on clinical neurology (28.78%), neurosciences (22.9%), and psychiatry (22.23%). In accordance with types of pain, headache (19.09%) was the most popular topic in the included papers on comorbid pain and depression research.

**Conclusions:**

The findings provide useful information for pain and depression researchers to detect new areas related to collaborators, cooperative institutions, popular topics, and research frontiers.

## 1. Introduction

Pain is the most pervasive physical symptom in primary care, which affects physical and emotive functions, deteriorates the quality of life, and reduces the ability to work [1–4]. The prevalence of chronic pain has been estimated to be as high as 40% in the United States [5]. The most common types of pain syndromes [6–8] include headache, low back pain, fibromyalgia, neuropathic pain, arthritis, cancer, and postsurgical pain. The total cost of pain has been calculated to be as high as 3% of the gross domestic product of European countries, which is larger than the cost of cancer or heart disease [9]. Depression is the most common psychological symptom and is a major public health concern in primary care [10, 11]. The prevalence of depressive symptoms or depression among medical students from 43 countries has been reported to be 27.2% [12], and the lifetime prevalence of depression in 30 countries has been calculated to be 10.8% [13]. In Japan, the total cost of depression among adults was estimated to be ¥2.0 trillion in 2005 [14].

Comorbid pain and depression occur with high prevalence in clinical observations, as high as 60% [4]. A number of studies have reported that antidepressant drugs have been used to treat pain since the 1960s [4, 15]. Pain intensity is aggravated by increasing levels of depression [16]. Although the neurobiological mechanisms of the interaction between pain and depression are unclear, human neuroimaging studies indicate that depression can influence changes in multiple brain regions including those related to pain processing and perception [17, 18].

While research on comorbid pain and depression is widely available worldwide, relatively few studies use bibliometric methods to analyze the general aspects of this research topic. Bibliometric studies are extensively used to determine trends of scientific research and involve quantitative analysis of published studies [19, 20]. This type of study could offer readers quantitative information on distribution by country, institution, author, and journal in a specific field. Over the last 10 years, bibliometric studies have been used to analyze scientific research in different areas, such as long noncoding RNA [21], paediatric pain [22], cancer rehabilitation [23], childhood immunization [24], eye disease [25], and drug delivery [26], worldwide.

To address the shortage of quantitative analysis of comorbid pain and depression research, the aim of this study is to systematically provide global scientific research in this field from 1980 to 2018. We used CiteSpace V (Drexel University, Philadelphia, United States) to perform a bibliometric study in Web of Science Core Collection, which is a tool frequently used to assess the trends in the global scientific research [27]. In this study, global scientific research on comorbid pain and depression includes the number of published papers, types of pain, distribution and collaborations between authors/institutions/countries, a citation burst analysis of keywords, cocitation analysis of authors and references.

## 2. Methods

### 2.1. Sources of the Data

The published papers were searched in the recent forty years (from 1980 to 2018). We downloaded and extracted the publications from Science Citation Index Expanded (SCI-Expanded) of Web of Science. The following terms were searched: Title=(pain∗ or headache∗ or migraine∗ or head-ache∗ or “head ache∗” or cephalalgi∗ or “abdominal ache∗” or fibromyalg∗ or “tummy ache∗” or “stomach ache∗” or “belly ache∗” or ear-ache∗ or earache∗ or tooth-ache∗ or toothache∗ or odontalgi∗ or neuralgi∗ or cervicodyn∗ or analg∗ or nocicept∗ or hyperalg∗ or hypoalg∗ or radiculalg∗ or colic or arthralg∗ or causalg∗ or maldyn∗ or eudyn∗ or ophthalmodyn∗ or cephalalg∗ or dysmenorrh∗ or sciatic∗ or otalg∗ or brachialg∗) and Title=(depression or depressions or depressed or despondent or gloomy or depressive).

### 2.2. Inclusion Criteria

We included articles, reviews, letters, and editorial materials published in different academic journals. Meeting abstracts, conference presentations, book reviews, news items, and corrections were excluded. Publications related to comorbid pain and depression research were included. The language was restricted to English, and no species limitations were specified.

### 2.3. Data Extraction

The two authors (Lin-Man Weng and Xue-Qiang Wang) extracted the publications and used EndNote (EndNote X7, Bld 7072, Thomson Research Soft, Stamford) and Microsoft Excel 2016 to perform the downloaded published studies. We extracted and recorded pertinent information, such as publication count, journal, institution, country, and citation frequency, as bibliometric indicators. The *H*-index is recognized as a measurement of the papers' citation frequency for academic journals or researchers. For example, the *H*-index is that an academic journal or researcher has *H* published papers which have at least *H* citation times per paper (If the *H*-index of one researcher is 10, that means that he had published at least 10 papers and each of these 10 papers had been cited at least 10 times.) [28]. Furthermore, these included studies that were classified into the following categories: (1) single- or multiple-authored publications (authors ≥ 2); (2) Web of Science subject category. We would select and assess the top 20 subject categories of Web of Science categories according to the included studies. Every paper or journal is assigned to at least one Web of Science category in Web of Science core collection. Thus, the sum of publications could be more than 2,519. Research areas were classified into five broad categories in Web of Science categories, and the five broad categories included 225 subject categories [29], such as anesthesiology, neurosciences, and psychiatry; (3) types of pain. We would choose and analyze the top ten types of pain (such as headache and low back pain) according to the included studies. Because some papers exceeded one type of pain, the sum of publications could be more than 2,519. For example, the paper by Gesztelyi and Bereczki included headaches and low back pain [30].

### 2.4. Statistical Methods

CiteSpace V and Microsoft Excel 2016 were used to (1) analyze the distribution by journals, years, countries, institutions, and authors, (2) assess the collaborations between countries, institutions, and authors, (3) perform analysis of citations and H-index, and (4) conduct analysis of reference and keywords. In addition, we calculated a percentage score each year for some categories (all the articles were divided into different categories, the number of publications per year of one category divided by the total number of publications of this category), such as single-authored publications and multiple-authored publications, subject categories of Web of Science, and types of pain. To assess whether the percentage statistically decreased or increased over time, the linear regression analyses were conducted with every respective category as the dependent variable and the year as the independent variable. The statistical analyses were performed with IBM SPSS Statistics 22.0 software (SPSS Inc., Chicago, USA). A *P* value less than 0.05 was considered as statistical significance.

## 3. Results

### 3.1. Publication Outputs and Growth Trends

**Figure 1:**
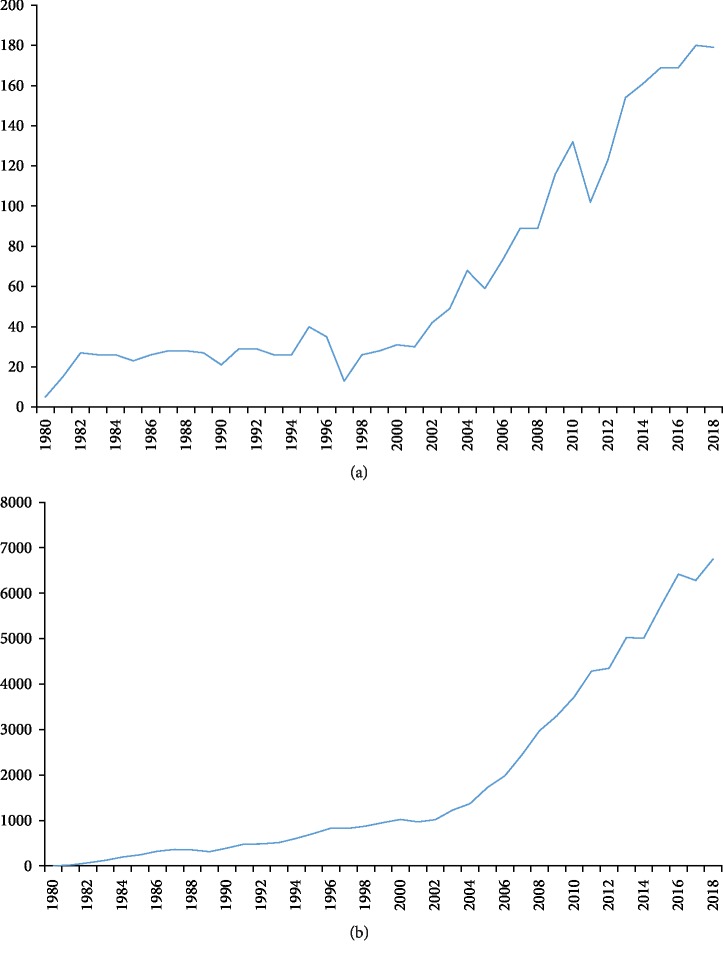
Number of publications and citations. (a) The number of annual publications on pain and depression research from 1980 to 2018; (b) the number of annual citations on pain and depression research from 1980 to 2018.

**Figure 2:**
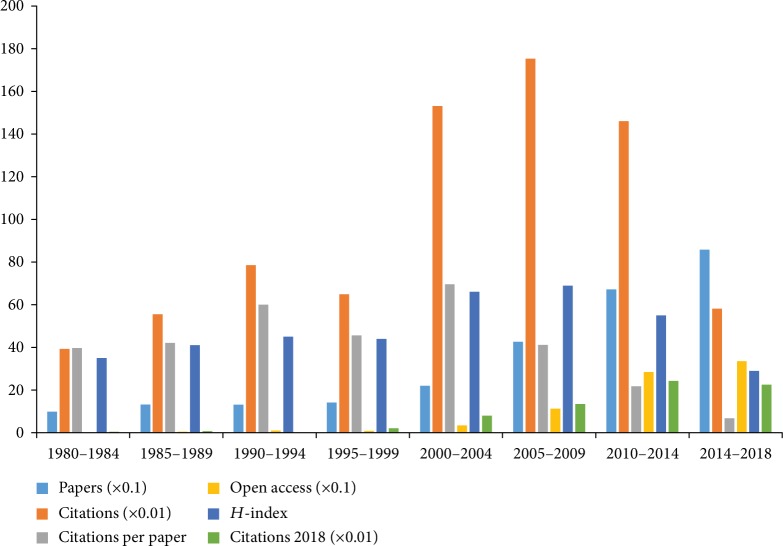
Number of papers, citations, citations per paper, open access paper, *H*-index, and citations in 2018 for each 5-year time period.

A total of 2,519 papers met the inclusion criteria; 1,264 papers (meeting abstracts, proceedings papers, notes, corrections, book reviews, and news items) and 151 non-English papers were excluded (Supplementary [Supplementary-material supplementary-material-1]). The overall trend of publications increased from five publications in 1980 to 179 publications in 2018 (Figure 1(a)). The results of linear regression analyses showed that the percentages had a significant increase over time in the recent forty years (*t* = 11.37, *P* < 0.001). All papers related to comorbid pain and depression research were cited 74,746 times (29.67 times per year, *H*-index 121), and the percentages had a significant increase over time (*t* = 12.47, *P* < 0.001) (Figure 1(b)). Among the eight 5 years (1980-1984, 1985-1989, 1990-1994, 1995-1999, 2000-2004, 2005-2009, 2010-2014, and 2014-2018), 2005-2009 had the largest number of citations (17,528) and the highest value of *H*-index (66), 2000-2004 had the largest number of citations per paper (69.58), 2010-2014 had the largest number of citations (2429) in 2018, and 2014-2018 had the largest number of publications (858) and open access papers (335) (Figure 2).

### 3.2. Distribution by Journals

The 2,519 papers on comorbid pain and depression research were published in 251 academic journals (Supplementary [Supplementary-material supplementary-material-1]). Among the top 20 journals by the number of publications (Table 1), *Pain*, in which impact factor (IF) 2017 was 5.559, contributed to the most publications on comorbid pain and depression research (136 publications, 5.39%), followed by *Clinical Journal of Pain* (IF 2017, 3.209; 57 publications; 2.26%), *Headache* (IF 2017, 3.091; 57 publications; 2.26%), and *Cephalalgia* (IF 2017, 3.886; 50 publications; 1.98%). The top 20 journals contributed to 29.37% of the total number of publications. The highest value of *H*-index was with *Pain* (97), the largest number of citations per paper was with *Psychosomatic Medicine* (IF 2017, 3.81), and the highest value of IF 2017 was with *Anesthesiology* (IF 2017, 6.523). In journal IF quartile of Web of Science, 55% of journals were Q1 (Q1 stands for the top 25% of the IF distribution) and 40% of journals were Q2 (Q2 stands for between top 50% and top 25% of the IF distribution) among the 20 journals.

**Figure 3:**
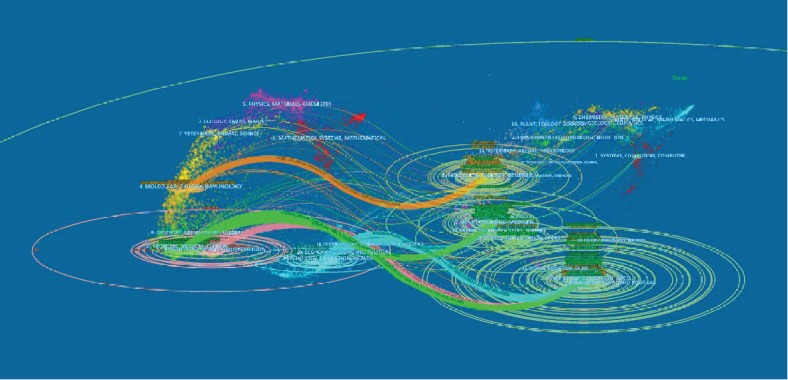
The dual-map overlay of journals related to pain and depression research.

A dual-map overlay of journals is presented in Figure 3. In the dual-map, the map of the citing journals is on the left and the map of the cited journals is on the right. The disciplines included in the journals are shown as labels in the dual map. The lines are considered citation connections, beginning from the citing journals to the cited journals. The dual-map overlay shows that the majority of papers were published in neurology, sports, and ophthalmology journals and these journals mostly cited journals from psychology, education, and social areas.

### 3.3. Subject Categories of Web of Science

**Figure 4:**
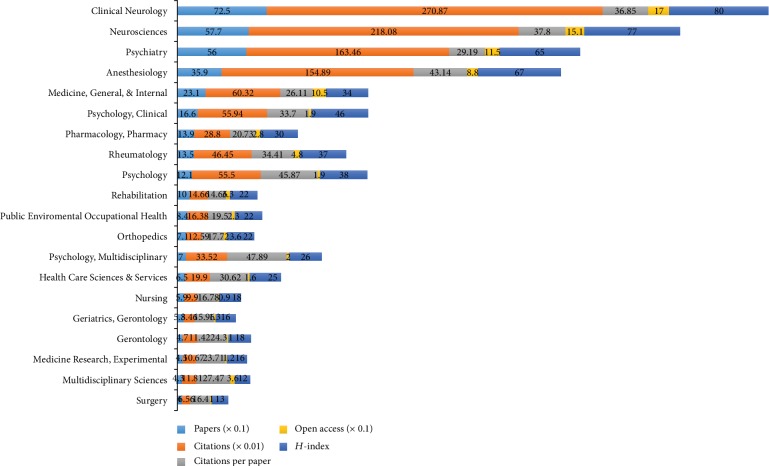
The number of papers, citations, citations per paper, open access papers, and H-index of the top 20 subject categories of Web of Science.

Research areas of the 2,519 papers on comorbid pain and depression research were assigned to 55 subject categories of Web of Science. In top 20 subject categories (Figure 4), according to the number of publications, *Clinical Neurology* had the largest number of publications (725), citations (27,087), and open access papers (170) and the highest value of H-index (80). The *Psychology Multidisciplinary* subject category had the largest number of citations per paper (47.89). Moreover, the results of linear regression analyses showed that the percentages of publication count per year had a statistical increase over time (*P* < 0.01) in the top 20 subject categories (*Clinical Neurology*, *Neurosciences*, *Psychiatry*, *Anesthesiology*, *Medicine General Internal*, *Psychology Clinical*, *Pharmacology Pharmacy*, *Rheumatology*, *Psychology*, *Rehabilitation*, *Public Environmental Occupational Health*, *Orthopedics*, *Psychology Multidisciplinary*, *Health Care Sciences Services*, *Nursing, Geriatrics Gerontology*, *Gerontology*, *Medicine Research Experimental*, *Multidisciplinary Sciences*, and *Surgery*).

### 3.4. Types of Pain

**Figure 5:**
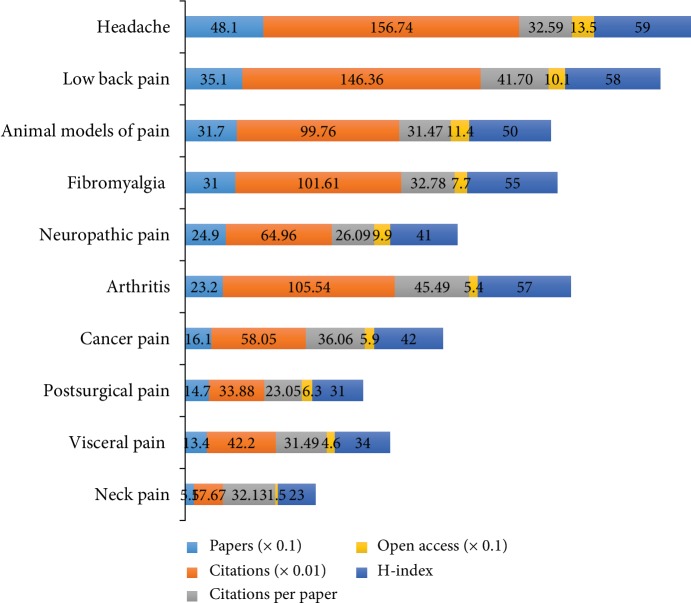
The number of papers, citations, citations per paper, open access papers and H-index of the top 10 types of pain.

As shown in Figure 5, according to the number of publications, headache (19.09%) was the most popular topic in the included papers on comorbid pain and depression research, followed by low back pain, animal models of pain, and fibromyalgia. Among the top 10 types of pain, headache had the largest number of publications (481), citations (15,674), and open access papers (135) and the highest value of *H*-index (59). Arthritis had the largest number of citations per paper (45.49). In addition, the results showed that the percentages of publication count per year had a significant increase over time (*P* < 0.01) in headache, low back pain, animal models of pain, fibromyalgia, neuropathic pain, arthritis, cancer pain, postsurgical pain, visceral pain, and neck pain.

### 3.5. Distribution by Countries and Institutions

**Figure 6:**
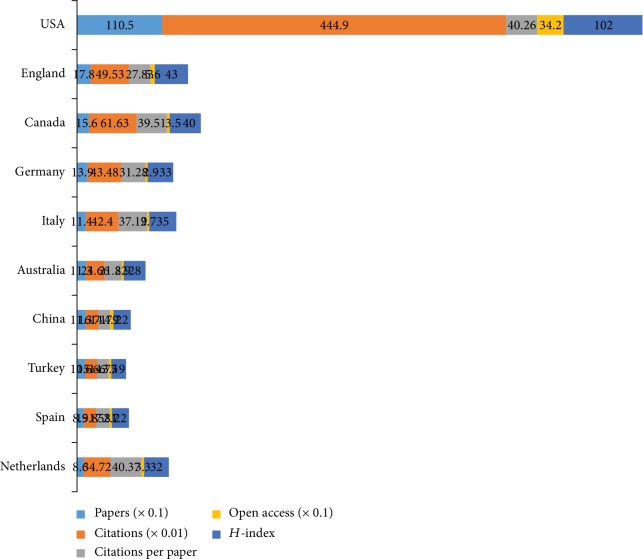
The number of papers, citations, citations per paper, open access papers, and *H*-index of the top 10 countries.

**Figure 7:**
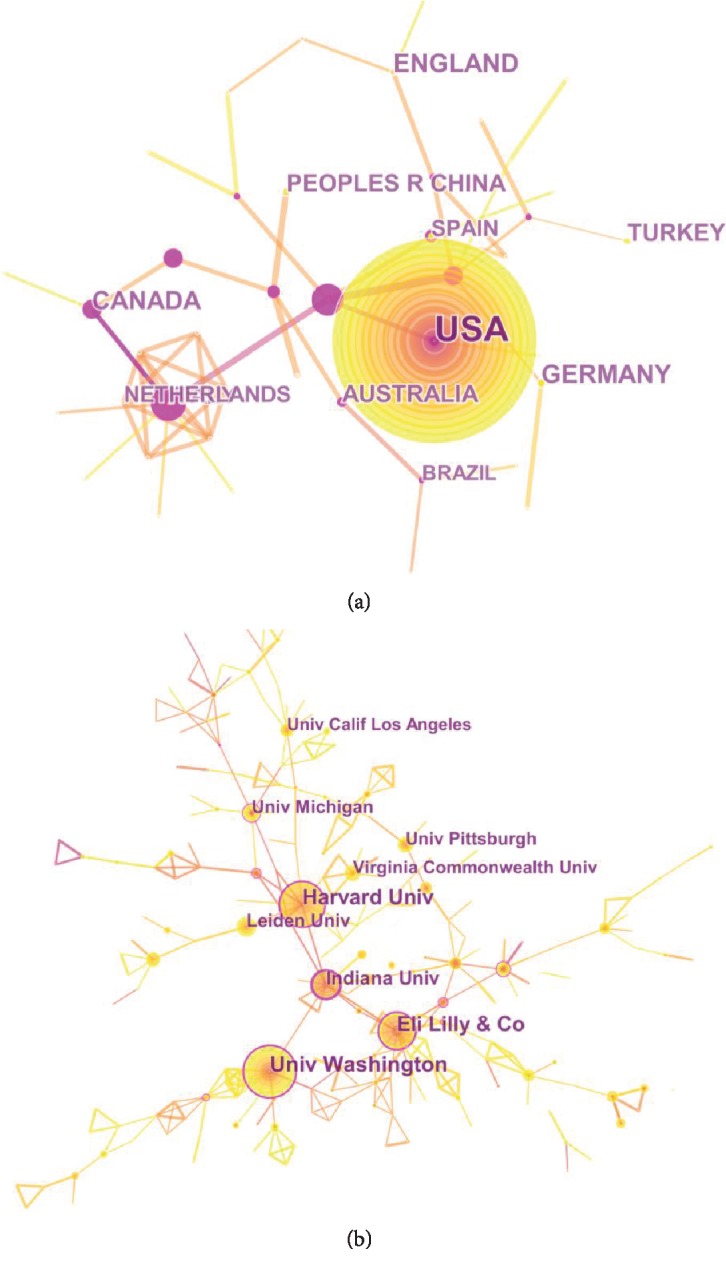
The analysis of countries and institutions. (a) Network map of countries/territories engaged in pain and depression research; (b) network map of institutions engaged in pain and depression research.

**Figure 8:**
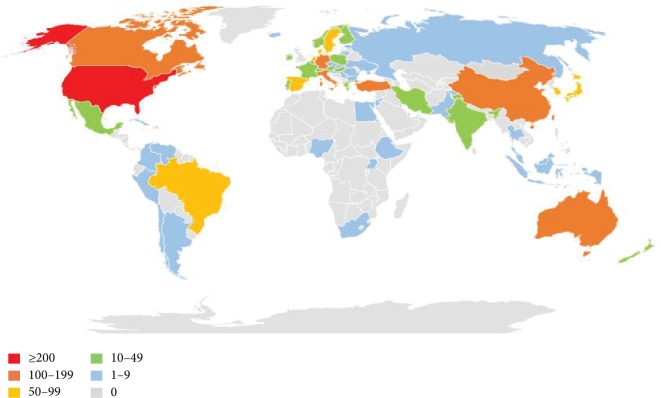
World map of total country output based on pain and depression research.

The 2,519 papers on comorbid pain and depression research were contributed by 75 countries/territories (Supplementary [Supplementary-material supplementary-material-1]).  Figure 6 shows the top 10 countries/territories by quantity of papers, showed as sums of paper fractions. The United States had the largest number of publications (1,105), citations (44,490), and open access papers (342) and the highest value of *H*-index (102). The Netherlands had the largest number of citations per paper (40.37). In relation to the top 10 countries that contributed to comorbid pain and depression research, USA led the first research echelon, followed by the England (178), Canada (156), and Germany (139). There were extensive collaborations between countries/territories (Figure 7(a)). In accordance with the number of published papers, the overview of all countries was presented in world map (Figure 8).

A total of 1,884 institutions contributed to the publications on comorbid pain and depression research (Supplementary [Supplementary-material supplementary-material-1]). The top 10 institutions (Supplementary [Supplementary-material supplementary-material-1]) contributed to 30.77% of the total number of publications. The University of California System had the largest number of publications (108), University of Washington had the largest number of citations (5,680), Eli Lilly had the largest number of citations per paper (108), and Harvard University had the largest number of open access papers (45) and the highest value of H-index (37).  Figure 7(b) showed the network map of institutions engaged in pain and depression research.

### 3.6. Distribution by Authors

**Figure 9:**
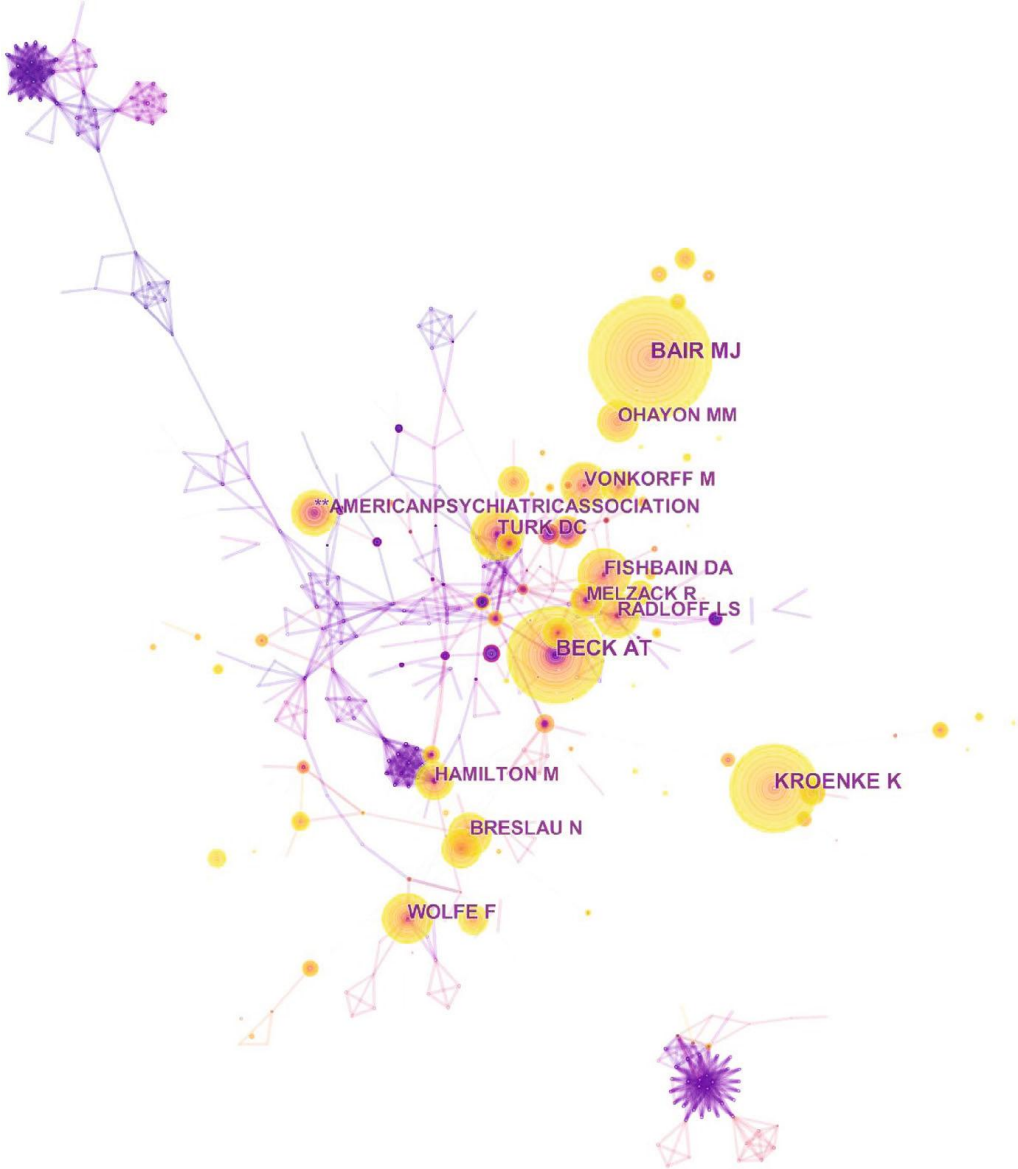
The analysis of authors. Network map of active authors that contributed to pain and depression research.

**Figure 10:**
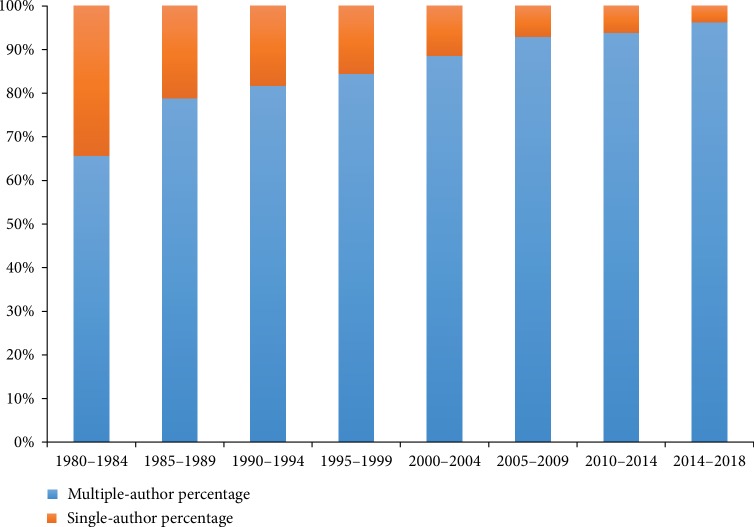
Trends in the percentage of single- vs multiple-authored articles per 5 years.

A total of 8,771 authors contributed to the total number of publications. The network map in Figure 9 outlines the cooperation between authors. Amongst the authors who had the most publications (Table 2), Kroenke K ranked the first with 35 publications, followed by Bair MJ with 21 publications, Negus SS with 19 publications, and Wang SJ with 19 publications. Amongst the top 10 cocited authors (Table 2), Bair MJ with 447 citations, followed by Beck AT with 403 citations, Kroenke K with 337 citations, and Turk DC with 236 citations were the most popular.  Figure 10 indicates that the percentages of multiple-author papers (≥2) increased from 65.66% in 1980–1984 to 96.27% in 2014–2018. The linear regression results revealed that the percentage of papers with multiple authors significantly increased and that with a single author significantly decreased (*t* = 11.76, *P* < 0.001).

### 3.7. Analysis of References

**Figure 11:**
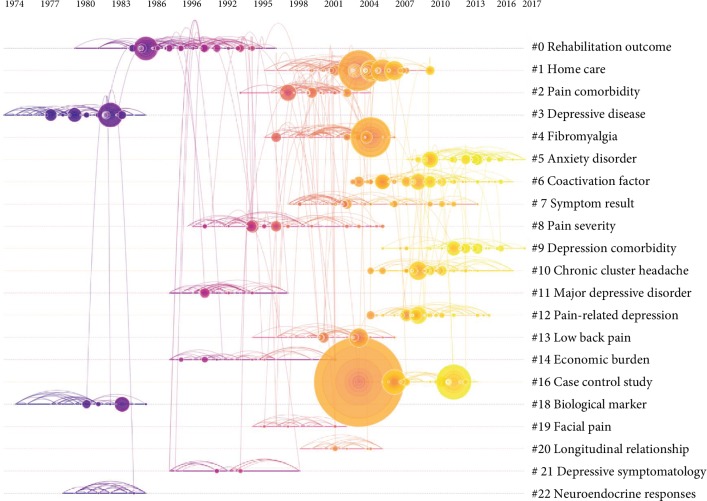
The analysis of references. Cocitation map (timeline view) of references from publications on to pain and depression research.

Analysis of references was regarded as an important indicator in bibliometric study. The scientific relevance of the published papers was presented in the cocitation map of references (Figure 11). The modularity *Q* score was 0.8897 (higher than 0.5), which indicated that the network was reasonably distributed to loosely coupled clusters. All clusters were traced by index terms extracted from the references. The “rehabilitation outcome” was labeled as the largest cluster #0, followed by “home care” as the second largest cluster #1, “pain comorbidity” as the third largest cluster #2, and “depressive disease.” As shown in Figure 11, the top 23 clusters were showed in a timeline view.

### 3.8. Analysis of Keywords

**Figure 12:**
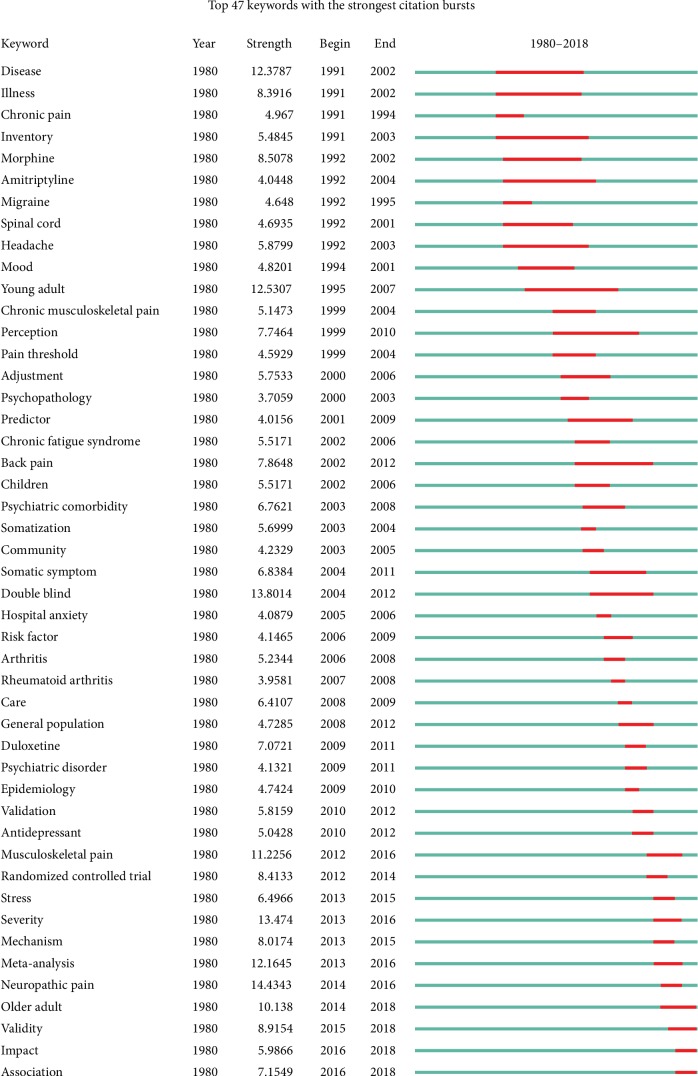
The keywords with the strongest citation bursts of publications on pain and depression research.

The keywords of the 2,519 published papers were extracted by CiteSpace V.  Figure 12 reveals the top 47 keywords with the strongest citation bursts. Keywords with the strongest citation bursts beginning in 1991 were as follows: “disease,” “illness,” “chronic pain,” and “inventory.” The top 47 keywords by the end of 2018 included “older adult” (2014–2018), “validity” (2015–2018), “impact” (2016–2018), and “association” (2016–2018).

### 3.9. Characteristics of Top 10 Papers Cited Most Frequently

Table 3 showed the top ten papers on comorbid pain and depression research with the most citation frequency. The top 10 papers contributed to 7.81% (5,831 citations) of the total number of citations. The article by Bair et al. [31] published in 2003 was the most cited (1,442 citations) paper, and the article is entitled “Depression and pain comorbidity-a literature review.” published in *Archives of Internal Medicine*. Among the top 10 papers, five [31–35] were published in journals with impact factor ≥ 10 (*Archives of Internal Medicine*, *Brain*, *Psychological Bulletin and Neuron*), two [36, 37] in journals with 5 ≤ impact factor < 10 (*Neurology*, *Arthritis & Rheumatology*), two [38, 39] in journals with 3 ≤ impact factor < 5 (*Clinical Journal of Pain*, *Journal of Pain*), and one [40] journal with 1 ≤ impact factor < 3 (*The Journal of Nervous and Mental Disease*).

## 4. Discussion

### 4.1. Global Trends of the Comorbidity of Pain and Depression Research

The results of our bibliometric study provide a systematic overview on the comorbidity of pain and depression research in the recent forty years. The global trends of published papers showed a statistical continued growth over time in the comorbidity of pain and depression research. Although the percentage of publication increased steadily year by year, a noticeable growth was found between 2001 and 2004 in the year-over-year percentage of publication. Among the eight 5 years, 2014-2018 had the largest number of publications (858) and 2005-2009 had the largest number of citations (17,528) and the highest value of *H*-index (66).

In accordance with the number of publications on comorbid pain and depression research, the top 20 journals contributed to 29.37% (740 publications) of the total number of publications. *Pain* contributed to the most publications on comorbid pain and depression research (5.39%) followed by *Clinical Journal of Pain* (2.26%), *Headache* (2.26%), and *Cephalalgia* (1.98%). According to the impact factors of the journals in the Journal Citation Reports (2017 edition), the impact factors were less than 10 among the top 20 journals. In the top 20 journals, the journals with 2 ≤ impact factor < 3 contribute to 40% of the top 20 journals, the journals with 3 ≤ impact factor < 5 contribute to 50%, and the journals with 5 ≤ impact factor < 10 contribute to 10%. However, according to journal IF quartile of Web of Science, 55% of the top 20 journals were Q1 and 40% of the top 20 journals were Q2.

In accordance with the number of publications, USA led the first research echelon (1,105), followed by England (178), Canada (156), and Germany (139). And the top 10 countries were from five European countries, three Asia-Pacific countries, and two American countries. As demonstrated in Figure 7, it had an extensive collaboration between countries. A total of 1,884 institutions contributed to the publications on comorbid pain and depression research. According to the number of publications, nine institutions were from USA and one from England. Compared with the cooperation of countries, the institutions were not significant.

### 4.2. Research Focuses on the Comorbidity of Pain and Depression Research

According to subject categories of Web of Science, the most popular research areas were *Clinical Neurology* (725), followed by *Neurosciences* (577), *Psychiatry* (560), and *Anesthesiology* (359). The top 10 subject categories were *Clinical Neurology*, *Neurosciences*, *Psychiatry*, *Anesthesiology*, *Medicine General Internal*, *Psychology Clinical*, *Pharmacology Pharmacy*, *Rheumatology*, *Psychology*, and *Rehabilitation.* On the basis of types of pain, research topics of the 2,519 papers mainly focused on headache and low back pain for the comorbid pain and depression research. Among the top 10 types of pain (headache, low back pain, animal models of pain, fibromyalgia, neuropathic pain, arthritis, cancer pain, postsurgical pain, visceral pain, and neck pain), headache had the largest number of publications (481), citations (15,674), and open access papers (135) and the highest value of *H*-index (59). According to the cocitation map of references, the “rehabilitation outcome” was labeled as the largest cluster #0, followed by “home care” as the second largest cluster #1, “pain comorbidity” as the third largest cluster #2, and “depressive disease.” According to the keywords with strongest citation bursts, the results found that the top 47 keywords that began in 1991 were as follows: “disease,” “illness,” “chronic pain,” and “inventory.” And the top 47 keywords by the end of 2018 were as follows: “older adult” (2014-2018), “validity” (2015-2018), “impact” (2016-2018), and “association” (2016-2018). In the last 10 years, because of the aging of the global population, the comorbidity of pain and depression research was rapidly increasing in the older adult. The comorbid pain and depression research gradually transitioned from phenomenon to mechanism. The study by Descalzi et al. [41] showed that their findings not only confirm molecular links on comorbid pain and depression but also provided a potential therapeutic approach.

### 4.3. Strengths and Limitations

This study is the first bibliometric analysis to assess the trends of the comorbid pain and depression research from SCI-Expanded of Web of Science in the recent forty years. Furthermore, to gain rich data, it was not restricted to one academic journal. The 2,519 papers on comorbid pain and depression research were published in 251 different academic journals. Moreover, bibliometric analysis of our study not only covered the number of publications, citations, journals, and collaborations between countries/institutions/authors but also included analysis of keywords, cocitation analysis on references and authors, Web of Science subject categories, and types of pain.

This bibliometric study has some limitations. The electronic database is limited to SCI-Expanded of Web of Science, and other electronic databases are not searched and analyzed, for example, PubMed, Embase, Scopus, Cochrane Library. Furthermore, the non-English papers were excluded. Most included papers use English in this study; however, the limitation may induce a publication bias. The last limitation is that influential publications were not cited with high citation frequency, since some potential influential papers were published recently, which could be not cited with frequent times.

## 5. Conclusions

This study provides historical insights into the trends of comorbidity of pain and depression research. The number of published papers significantly increased over the last 40 years, and the overall trend of publications increased from 5 publications in 1980 to 179 publications in 2018. Although this study presents several limitations, it adequately exposes the trends of comorbidity of pain and depression research. Based on the type of pain, the research topics of the 2,519 papers included in this work mainly focused on headache, low back pain, and fibromyalgia. The results of our study could provide useful information for pain and depression researchers, funding agencies, and policy makers.

## Figures and Tables

**Table 1:** The top 20 journals of origin of papers in the pain and depression research.

Journals	Papers	Citations (WoS)	Citations per paper	Open access	WoS categories	IF 2017	Quartile	*H*-index
Pain	136	9036	66.44	18	Anesthesiology; Clinical Neurology; Neurosciences	5.559	Q1; Q1; Q1	56
Clinical Journal of Pain	57	2465	43.25	4	Anesthesiology; Clinical Neurology	3.209	Q2; Q2	23
Headache	57	1671	29.32	10	Clinical Neurology	3.091	Q2	26
Cephalalgia	50	1130	22.60	4	Clinical Neurology; Neurosciences	3.886	Q1; Q2	18
Pain Medicine	47	1022	21.74	45	Anesthesiology; Medicine, General, & Internal	2.782	Q2; Q1	18
Journal of Pain	43	1703	39.60	6	Clinical Neurology; Neurosciences	4.859	Q1; Q1	22
Journal of Affective Disorders	41	1119	27.29	3	Clinical Neurology; Psychiatry	3.786	Q1; Q1	21
European Journal of Pain	40	826	20.65	6	Anesthesiology; Clinical Neurology; Neurosciences	2.991	Q2; Q2; Q3	17
Journal of Clinical Psychiatry	28	1342	47.93	1	Psychiatry; Psychology, Clinical	4.247	Q1; Q1; Q1	18
Journal of Psychosomatic Research	27	1089	40.33	1	Psychiatry	2.947	Q2	16
Journal of Headache and Pain	26	377	14.50	26	Clinical Neurology; Neurosciences	3.403	Q2; Q2	11
General Hospital Psychiatry	25	706	28.24	10	Psychiatry	2.989	Q2	15
Psychosomatic Medicine	24	1719	71.63	5	Psychiatry; Psychology; Psychology, Multidisciplinary	3.81	Q1; Q1; Q1	20
PLoS One	24	274	11.42	24	Multidisciplinary Sciences	2.776	Q1	9
Psychosomatics	23	693	30.13	2	Psychiatry; Psychology	2.534	Q2; Q2	12
Journal of Pain and Symptom Management	22	975	44.32	3	Clinical Neurology; Health Care Sciences & Services; Medicine, General, & Internal	2.498	Q2; Q1; Q1	16
Anesthesia and Analgesia	19	493	25.95	3	Anesthesiology	3.463	Q1	11
Anesthesiology	18	625	34.72	3	Anesthesiology	6.523	Q1	10
Journal of Nervous and Mental Disease	17	799	47	0	Clinical Neurology; Psychiatry	2.776	Q3; Q3	10
Journal of Rheumatology	16	891	55.69	0	Rheumatology	3.47	Q2	16

**Table 2:** The top 10 authors, cocited authors, and cocited references in the pain and depression research.

Author	Published articles	Cocited author	Cited times	Cocited reference	Cited times
Kroenke K	35	Bair MJ	447	Bair MJ, 2003, Arch Intern Med, V163, P2433	154
Bair MJ	21	Beck AT	403	Bair MJ, 2004, Psychosom Med, V66, P17	72
Negus SS	19	Kroenke K	337	Ohayon MM, 2003, Arch Gen Psychiat, V60, P39	72
Wang SJ	19	Turk DC	236	Kroenke K, 2011, J Pain, V12, P964	61
Wu JW	18	Fishbain DA	228	Blumer D, 1982, J Nerv Ment Dis, V170, P381	46
Ferrari MD	15	Wolfe F	223	Romano JM, 1985, Psychol Bull, V97, P18	46
Hung CI	15	Vonkorff M	202	Arnow BA, 2006, Psychosom Med, V68, P262	44
Liu CY	15	^∗∗^American Psychiatric Association	199	Karp JF, 2005, J Clin Psychiat, V66, P591	40
Arnold LM	13	Radloff LS	195	Currie SR, 2004, Pain, V107, P54	38
Ayata C	13	Breslau N	182	Demyttenaere K, 2006, J Affect Disorders, V92, P185	37

**Table 3:** The top 10 papers with the most citation frequency in the pain and depression research.

Title	First author	Journal	Impact factor	Year	Citations (WoS)	WoS categories	Category ranking
Depression and pain comorbidity-a literature review	Bair MJ	Archives of Internal Medicine	17.33 IF 2014	2003	1442	Medicine, General, & Internal	6/154
Pathophysiology of the migraine aura. The spreading depression theory	Lauritzen M.	Brain	10.848	1994	785	Clinical Neurology; Neurosciences	6/197; 13/261
Chronic pain-associated depression: antecedent or consequence of chronic pain? A review	Fishbain DA	Clinical Journal of Pain	3.209	1997	585	Anesthesiology; Clinical Neurology	11/31; 69/197
Chronic pain and depression: does the evidence support a relationship?	Romano JM	Psychological Bulletin	13.25	1985	513	Psychology; Psychology, Multidisciplinary	3/78; 4/135
Amitriptyline relieves diabetic neuropathy pain in patients with normal or depressed mood	Max MB	Neurology	8.055	1987	462	Clinical Neurology	13/197
Chronic pain as a variant of depressive disease: the pain-prone disorder	Blumer D	The Journal of Nervous and Mental Disease	1.94	1982	460	Clinical Neurology; Psychiatry	134/197; 89/142
Explaining high rates of depression in chronic pain: a diathesis-stress framework	Banks SM	Psychological Bulletin	13.25	1996	434	Psychology; Psychology, Multidisciplinary	3/78; 4/135
A double-blind, multicenter trial comparing duloxetine with placebo in the treatment of fibromyalgia patients with or without major depressive disorder	Arnold LM	Arthritis & Rheumatology	7.379	2004	392	Rheumatology	1/22
Common chronic pain conditions in developed and developing countries: gender and age differences and comorbidity with depression-anxiety disorders	Tsang A	Journal of Pain	4.859	2008	382	Clinical Neurology; Neurosciences	27/197; 51/261
A Cacna1a knockin migraine mouse model with increased susceptibility to cortical spreading depression	van den Maagdenberg AMJM	Neuron	14.319	2004	376	Neurosciences	7/261

## Data Availability

All research data used to support the findings of this study are included within the article and the supplementary information file.

## Supplementary Materials

Supplementary 1 Supplementary Figure 1: Overview of the paper selection process.

Supplementary 2 Supplementary Figure 2: The number of papers, citations, citations per paper, open access papers, and H-index of the top 10 institutions.

Supplementary 3 Supplementary Table 1: Raw data on journal sources of pain and depression publications.

Supplementary 4 Supplementary Table 2: Raw data on countries/territories involved in pain and depression publications.

Supplementary 5 Supplementary Table 3: Raw data on institutions involved in pain and depression publications.
