# Supplementary material for: Bibliometric Study of the Comorbidity of Pain and Depression Research
Source: Neural Plast. 2019 Oct 23;2019:1657498. doi: 10.1155/2019/1657498 (PMC6854239; doi:10.1155/2019/1657498)
Supplement: Supplementary 1 — Supplementary Figure 1: Overview of the paper selection process. [file 1657498.f1.docx]

3934 Papers Identified from Web of Science Core Collection

2670 Papers Identified

2519 Papers Identified

Excluded 1264 Papers : Meeting Abstract, Proceedings Paper, Note, Correction, Book Review, News Item.

Excluded Papers 151 Non-English Papers

Supplementary Figure 1. Overview of paper selection process.
