# Supplementary material for: Bibliometric Study of the Comorbidity of Pain and Depression Research
Source: Neural Plast. 2019 Oct 23;2019:1657498. doi: 10.1155/2019/1657498 (PMC6854239; doi:10.1155/2019/1657498)
Supplement: Supplementary 2 — Supplementary Figure 2: The number of papers, citations, citations per paper, open access papers, and H-index of the top 10 institutions. [file 1657498.f2.docx]

Supplementary Figure 2. The number of papers, citations, citations per paper, open access papers and H-index of the top 10 institutions.
