# Supplementary material for: Bibliometric Study of the Comorbidity of Pain and Depression Research
Source: Neural Plast. 2019 Oct 23;2019:1657498. doi: 10.1155/2019/1657498 (PMC6854239; doi:10.1155/2019/1657498)
Supplement: Supplementary 3 — Supplementary Table 1: Raw data on journal sources of pain and depression publications. [file 1657498.f3.doc]

**Supplementary Table 1. Raw data on journal sources of pain and depression publications.**

| **Source Titles** | **Records** | **% of 2519** |
| --- | --- | --- |
| PAIN | 136 | 5.399 |
| CLINICAL JOURNAL OF PAIN | 57 | 2.263 |
| HEADACHE | 57 | 2.263 |
| CEPHALALGIA | 50 | 1.985 |
| PAIN MEDICINE | 47 | 1.866 |
| JOURNAL OF PAIN | 43 | 1.707 |
| JOURNAL OF AFFECTIVE DISORDERS | 41 | 1.628 |
| EUROPEAN JOURNAL OF PAIN | 40 | 1.588 |
| JOURNAL OF CLINICAL PSYCHIATRY | 28 | 1.112 |
| JOURNAL OF PSYCHOSOMATIC RESEARCH | 27 | 1.072 |
| JOURNAL OF HEADACHE AND PAIN | 26 | 1.032 |
| GENERAL HOSPITAL PSYCHIATRY | 25 | 0.992 |
| PLOS ONE | 24 | 0.953 |
| PSYCHOSOMATIC MEDICINE | 24 | 0.953 |
| PSYCHOSOMATICS | 23 | 0.913 |
| JOURNAL OF PAIN AND SYMPTOM MANAGEMENT | 22 | 0.873 |
| ANESTHESIA AND ANALGESIA | 19 | 0.754 |
| ANESTHESIOLOGY | 18 | 0.715 |
| JOURNAL OF NERVOUS AND MENTAL DISEASE | 17 | 0.675 |
| JOURNAL OF RHEUMATOLOGY | 16 | 0.635 |
| CLINICAL AND EXPERIMENTAL RHEUMATOLOGY | 15 | 0.595 |
| NEUROLOGICAL SCIENCES | 15 | 0.595 |
| NEUROLOGY | 15 | 0.595 |
| NEUROPSYCHIATRIC DISEASE AND TREATMENT | 15 | 0.595 |
| NEUROSCIENCE LETTERS | 15 | 0.595 |
| JOURNAL OF PSYCHIATRIC RESEARCH | 14 | 0.556 |
| PAIN MANAGEMENT NURSING | 14 | 0.556 |
| BRAIN RESEARCH | 13 | 0.516 |
| BRITISH JOURNAL OF PSYCHIATRY | 13 | 0.516 |
| RHEUMATOLOGY INTERNATIONAL | 13 | 0.516 |
| BMC PSYCHIATRY | 12 | 0.476 |
| INTERNATIONAL JOURNAL OF BEHAVIORAL MEDICINE | 12 | 0.476 |
| JOURNAL OF MUSCULOSKELETAL PAIN | 12 | 0.476 |
| JOURNAL OF PEDIATRIC PSYCHOLOGY | 12 | 0.476 |
| ARQUIVOS DE NEURO PSIQUIATRIA | 11 | 0.437 |
| BMC MUSCULOSKELETAL DISORDERS | 11 | 0.437 |
| INTERNATIONAL JOURNAL OF PSYCHIATRY IN MEDICINE | 11 | 0.437 |
| JOURNAL OF PAIN RESEARCH | 11 | 0.437 |
| JOURNAL OF PHARMACOLOGY AND EXPERIMENTAL THERAPEUTICS | 11 | 0.437 |
| ARTHRITIS CARE RESEARCH | 10 | 0.397 |
| PSYCHIATRY RESEARCH | 10 | 0.397 |
| ANNALS OF NEUROLOGY | 9 | 0.357 |
| CLINICAL RHEUMATOLOGY | 9 | 0.357 |
| CURRENT MEDICAL RESEARCH AND OPINION | 9 | 0.357 |
| INTERNATIONAL JOURNAL OF GERIATRIC PSYCHIATRY | 9 | 0.357 |
| JOURNAL OF CLINICAL PSYCHOLOGY | 9 | 0.357 |
| REHABILITATION PSYCHOLOGY | 9 | 0.357 |
| AMERICAN JOURNAL OF PSYCHIATRY | 8 | 0.318 |
| ARCHIVES OF PHYSICAL MEDICINE AND REHABILITATION | 8 | 0.318 |
| BIOLOGICAL PSYCHIATRY | 8 | 0.318 |
| COMPREHENSIVE PSYCHIATRY | 8 | 0.318 |
| EUROPEAN JOURNAL OF PHARMACOLOGY | 8 | 0.318 |
| INTERNATIONAL JOURNAL OF CLINICAL PRACTICE | 8 | 0.318 |
| JOURNAL OF BEHAVIORAL MEDICINE | 8 | 0.318 |
| JOURNAL OF THE ROYAL SOCIETY OF MEDICINE | 8 | 0.318 |
| PAIN RESEARCH MANAGEMENT | 8 | 0.318 |
| PSYCHO ONCOLOGY | 8 | 0.318 |
| ACTA PSYCHIATRICA SCANDINAVICA | 7 | 0.278 |
| BRAIN BEHAVIOR AND IMMUNITY | 7 | 0.278 |
| CANADIAN JOURNAL OF PSYCHIATRY REVUE CANADIENNE DE PSYCHIATRIE | 7 | 0.278 |
| DISABILITY AND REHABILITATION | 7 | 0.278 |
| FRONTIERS IN PSYCHOLOGY | 7 | 0.278 |
| HEALTH PSYCHOLOGY | 7 | 0.278 |
| JOURNAL OF CLINICAL PSYCHOPHARMACOLOGY | 7 | 0.278 |
| JOURNAL OF NEUROSCIENCE | 7 | 0.278 |
| NEUROREPORT | 7 | 0.278 |
| POSTGRADUATE MEDICINE | 7 | 0.278 |
| PSYCHOLOGICAL MEDICINE | 7 | 0.278 |
| PSYCHOLOGY HEALTH MEDICINE | 7 | 0.278 |
| PSYCHOTHERAPY AND PSYCHOSOMATICS | 7 | 0.278 |
| AGING MENTAL HEALTH | 6 | 0.238 |
| AMERICAN JOURNAL OF GERIATRIC PSYCHIATRY | 6 | 0.238 |
| BMJ OPEN | 6 | 0.238 |
| BRITISH JOURNAL OF ANAESTHESIA | 6 | 0.238 |
| BRITISH JOURNAL OF PHARMACOLOGY | 6 | 0.238 |
| CLINICAL ORTHOPAEDICS AND RELATED RESEARCH | 6 | 0.238 |
| JOURNAL OF BACK AND MUSCULOSKELETAL REHABILITATION | 6 | 0.238 |
| JOURNAL OF CONSULTING AND CLINICAL PSYCHOLOGY | 6 | 0.238 |
| JOURNAL OF REHABILITATION MEDICINE | 6 | 0.238 |
| LANCET | 6 | 0.238 |
| NEUROSCIENCE | 6 | 0.238 |
| NORDIC JOURNAL OF PSYCHIATRY | 6 | 0.238 |
| PAIN PRACTICE | 6 | 0.238 |
| PSYCHIATRIA DANUBINA | 6 | 0.238 |
| SPINE | 6 | 0.238 |
| ACTA ANAESTHESIOLOGICA SCANDINAVICA | 5 | 0.198 |
| AMERICAN JOURNAL OF CARDIOLOGY | 5 | 0.198 |
| ANAESTHESIA | 5 | 0.198 |
| ANNALS OF BEHAVIORAL MEDICINE | 5 | 0.198 |
| ARCHIVES OF INTERNAL MEDICINE | 5 | 0.198 |
| ARTHRITIS AND RHEUMATISM | 5 | 0.198 |
| ARTHRITIS RHEUMATISM ARTHRITIS CARE RESEARCH | 5 | 0.198 |
| CANADIAN JOURNAL OF ANAESTHESIA JOURNAL CANADIEN D ANESTHESIE | 5 | 0.198 |
| EUROPEAN SPINE JOURNAL | 5 | 0.198 |
| EVIDENCE BASED COMPLEMENTARY AND ALTERNATIVE MEDICINE | 5 | 0.198 |
| EXPERIMENTAL NEUROLOGY | 5 | 0.198 |
| HUMAN PSYCHOPHARMACOLOGY CLINICAL AND EXPERIMENTAL | 5 | 0.198 |
| INTERNATIONAL JOURNAL OF NEUROSCIENCE | 5 | 0.198 |
| JOURNAL OF ADVANCED NURSING | 5 | 0.198 |
| JOURNAL OF CLINICAL EPIDEMIOLOGY | 5 | 0.198 |
| JOURNAL OF FAMILY PRACTICE | 5 | 0.198 |
| JOURNAL OF GENERAL INTERNAL MEDICINE | 5 | 0.198 |
| JOURNAL OF HEALTH PSYCHOLOGY | 5 | 0.198 |
| JOURNAL OF PALLIATIVE MEDICINE | 5 | 0.198 |
| JOURNAL OF THE AMERICAN GERIATRICS SOCIETY | 5 | 0.198 |
| LIFE SCIENCES | 5 | 0.198 |
| PAIN PHYSICIAN | 5 | 0.198 |
| PHARMACOPSYCHIATRY | 5 | 0.198 |
| PROGRESS IN NEURO PSYCHOPHARMACOLOGY BIOLOGICAL PSYCHIATRY | 5 | 0.198 |
| PSYCHIATRY AND CLINICAL NEUROSCIENCES | 5 | 0.198 |
| PSYCHIATRY INVESTIGATION | 5 | 0.198 |
| PSYCHONEUROENDOCRINOLOGY | 5 | 0.198 |
| QUALITY OF LIFE RESEARCH | 5 | 0.198 |
| SLEEP | 5 | 0.198 |
| SPINE JOURNAL | 5 | 0.198 |
| ACTA NEUROLOGICA SCANDINAVICA | 4 | 0.159 |
| ACTA PAEDIATRICA | 4 | 0.159 |
| ADVANCES IN PAIN RESEARCH AND THERAPY | 4 | 0.159 |
| AIDS CARE PSYCHOLOGICAL AND SOCIO MEDICAL ASPECTS OF AIDS HIV | 4 | 0.159 |
| AMERICAN JOURNAL OF MEDICINE | 4 | 0.159 |
| ARTHRITIS RESEARCH THERAPY | 4 | 0.159 |
| BIOLOGICAL PSYCHOLOGY | 4 | 0.159 |
| BRITISH JOURNAL OF CLINICAL PSYCHOLOGY | 4 | 0.159 |
| BRITISH JOURNAL OF MEDICAL PSYCHOLOGY | 4 | 0.159 |
| CLINICAL NEUROPHYSIOLOGY | 4 | 0.159 |
| CURRENT PAIN AND HEADACHE REPORTS | 4 | 0.159 |
| EUROPEAN JOURNAL OF PSYCHIATRY | 4 | 0.159 |
| EUROPEAN PSYCHIATRY | 4 | 0.159 |
| FRONTIERS IN BEHAVIORAL NEUROSCIENCE | 4 | 0.159 |
| FRONTIERS IN NEUROLOGY | 4 | 0.159 |
| HOSPITAL AND COMMUNITY PSYCHIATRY | 4 | 0.159 |
| INTERNATIONAL JOURNAL OF NURSING STUDIES | 4 | 0.159 |
| INTERNATIONAL JOURNAL OF PSYCHIATRY IN CLINICAL PRACTICE | 4 | 0.159 |
| INTERNATIONAL JOURNAL OF RHEUMATIC DISEASES | 4 | 0.159 |
| INTERNATIONAL PSYCHOGERIATRICS | 4 | 0.159 |
| JOURNAL OF ARTHROPLASTY | 4 | 0.159 |
| JOURNAL OF CLINICAL NEUROSCIENCE | 4 | 0.159 |
| JOURNAL OF NEUROLOGY NEUROSURGERY AND PSYCHIATRY | 4 | 0.159 |
| JOURNAL OF NEUROPHYSIOLOGY | 4 | 0.159 |
| JOURNAL OF ORAL REHABILITATION | 4 | 0.159 |
| JOURNAL OF OROFACIAL PAIN | 4 | 0.159 |
| JOURNAL OF PHYSICAL THERAPY SCIENCE | 4 | 0.159 |
| JOURNAL OF SHOULDER AND ELBOW SURGERY | 4 | 0.159 |
| JOURNAL OF WOMENS HEALTH | 4 | 0.159 |
| MEDICINE | 4 | 0.159 |
| MOLECULAR PAIN | 4 | 0.159 |
| NEUROCHEMISTRY INTERNATIONAL | 4 | 0.159 |
| NEUROPSYCHOBIOLOGY | 4 | 0.159 |
| NEUROPSYCHOPHARMACOLOGY | 4 | 0.159 |
| PAKISTAN JOURNAL OF MEDICAL SCIENCES | 4 | 0.159 |
| PHYSICAL THERAPY | 4 | 0.159 |
| PHYSIOLOGY BEHAVIOR | 4 | 0.159 |
| PSYCHOLOGY HEALTH | 4 | 0.159 |
| PSYCHOPATHOLOGY | 4 | 0.159 |
| PSYCHOPHARMACOLOGY | 4 | 0.159 |
| SCANDINAVIAN JOURNAL OF RHEUMATOLOGY | 4 | 0.159 |
| SCIENCE | 4 | 0.159 |
| SCIENTIFIC REPORTS | 4 | 0.159 |
| SOCIAL PSYCHIATRY AND PSYCHIATRIC EPIDEMIOLOGY | 4 | 0.159 |
| SOUTHERN MEDICAL JOURNAL | 4 | 0.159 |
| SPINAL CORD | 4 | 0.159 |
| TURKISH JOURNAL OF RHEUMATOLOGY | 4 | 0.159 |
| ACTA MEDICA MEDITERRANEA | 3 | 0.119 |
| ACTA NEUROLOGICA BELGICA | 3 | 0.119 |
| ACTA OBSTETRICIA ET GYNECOLOGICA SCANDINAVICA | 3 | 0.119 |
| AMERICAN HEART JOURNAL | 3 | 0.119 |
| ANNALS OF INTERNAL MEDICINE | 3 | 0.119 |
| ASIA PACIFIC PSYCHIATRY | 3 | 0.119 |
| AUSTRALIAN NEW ZEALAND JOURNAL OF OBSTETRICS GYNAECOLOGY | 3 | 0.119 |
| BEHAVIORAL MEDICINE | 3 | 0.119 |
| BEHAVIOUR RESEARCH AND THERAPY | 3 | 0.119 |
| BEHAVIOURAL BRAIN RESEARCH | 3 | 0.119 |
| BIPOLAR DISORDERS | 3 | 0.119 |
| BMC NEUROLOGY | 3 | 0.119 |
| BMC PUBLIC HEALTH | 3 | 0.119 |
| BRAIN | 3 | 0.119 |
| BRITISH HEART JOURNAL | 3 | 0.119 |
| BULLETIN OF EXPERIMENTAL BIOLOGY AND MEDICINE | 3 | 0.119 |
| CHINESE MEDICAL JOURNAL | 3 | 0.119 |
| CLINICAL JOURNAL OF THE AMERICAN SOCIETY OF NEPHROLOGY | 3 | 0.119 |
| CLINICAL NEUROLOGY AND NEUROSURGERY | 3 | 0.119 |
| CNS SPECTRUMS | 3 | 0.119 |
| DEPRESSION AND ANXIETY | 3 | 0.119 |
| DRUGS AGING | 3 | 0.119 |
| EUROPEAN NEUROLOGY | 3 | 0.119 |
| EXPERIMENTAL BRAIN RESEARCH | 3 | 0.119 |
| EXPLORE THE JOURNAL OF SCIENCE AND HEALING | 3 | 0.119 |
| FOOT ANKLE INTERNATIONAL | 3 | 0.119 |
| FRONTIERS IN PSYCHIATRY | 3 | 0.119 |
| FUNCTIONAL NEUROLOGY | 3 | 0.119 |
| HEALTH AND QUALITY OF LIFE OUTCOMES | 3 | 0.119 |
| HEART LUNG | 3 | 0.119 |
| JAMA JOURNAL OF THE AMERICAN MEDICAL ASSOCIATION | 3 | 0.119 |
| JCR JOURNAL OF CLINICAL RHEUMATOLOGY | 3 | 0.119 |
| JOURNAL OF ABNORMAL PSYCHOLOGY | 3 | 0.119 |
| JOURNAL OF ADDICTION MEDICINE | 3 | 0.119 |
| JOURNAL OF AGING AND HEALTH | 3 | 0.119 |
| JOURNAL OF ALTERNATIVE AND COMPLEMENTARY MEDICINE | 3 | 0.119 |
| JOURNAL OF CLINICAL ANESTHESIA | 3 | 0.119 |
| JOURNAL OF CLINICAL PSYCHOLOGY IN MEDICAL SETTINGS | 3 | 0.119 |
| JOURNAL OF INTERPERSONAL VIOLENCE | 3 | 0.119 |
| JOURNAL OF NEURAL TRANSMISSION | 3 | 0.119 |
| JOURNAL OF OCCUPATIONAL AND ENVIRONMENTAL MEDICINE | 3 | 0.119 |
| JOURNAL OF PEDIATRICS | 3 | 0.119 |
| JOURNAL OF PERSONALITY ASSESSMENT | 3 | 0.119 |
| JOURNAL OF PHYSIOLOGY LONDON | 3 | 0.119 |
| JOURNAL OF REHABILITATION RESEARCH AND DEVELOPMENT | 3 | 0.119 |
| JOURNAL OF SPINAL CORD MEDICINE | 3 | 0.119 |
| JOURNAL OF THE PAKISTAN MEDICAL ASSOCIATION | 3 | 0.119 |
| JOURNALS OF GERONTOLOGY | 3 | 0.119 |
| KNEE SURGERY SPORTS TRAUMATOLOGY ARTHROSCOPY | 3 | 0.119 |
| MANUAL THERAPY | 3 | 0.119 |
| MEDICAL CARE | 3 | 0.119 |
| MEDICAL HYPOTHESES | 3 | 0.119 |
| NEUROPHARMACOLOGY | 3 | 0.119 |
| NEUROSCIENCE BULLETIN | 3 | 0.119 |
| NEUROSURGERY | 3 | 0.119 |
| PEDIATRICS | 3 | 0.119 |
| PHARMACOLOGY BIOCHEMISTRY AND BEHAVIOR | 3 | 0.119 |
| PSYCHIATRIC SERVICES | 3 | 0.119 |
| PSYCHOGERIATRICS | 3 | 0.119 |
| REGIONAL ANESTHESIA AND PAIN MEDICINE | 3 | 0.119 |
| REVISTA BRASILEIRA DE REUMATOLOGIA | 3 | 0.119 |
| RHEUMATOLOGY | 3 | 0.119 |
| SOUTH AFRICAN MEDICAL JOURNAL | 3 | 0.119 |
| SUPPORTIVE CARE IN CANCER | 3 | 0.119 |
| TURKISH JOURNAL OF MEDICAL SCIENCES | 3 | 0.119 |
| TURKISH JOURNAL OF PHYSICAL MEDICINE AND REHABILITATION | 3 | 0.119 |
| TURKISH NEUROSURGERY | 3 | 0.119 |
| WORLD NEUROSURGERY | 3 | 0.119 |
| ACTA CLINICA CROATICA | 2 | 0.079 |
| ACTA ODONTOLOGICA SCANDINAVICA | 2 | 0.079 |
| ACTA ORTOPEDICA BRASILEIRA | 2 | 0.079 |
| ACTA PHARMACOLOGICA SINICA | 2 | 0.079 |
| ACTA PHYSIOLOGICA SCANDINAVICA | 2 | 0.079 |
| AGE AND AGEING | 2 | 0.079 |
| AGING CLINICAL AND EXPERIMENTAL RESEARCH | 2 | 0.079 |
| AMERICAN FAMILY PHYSICIAN | 2 | 0.079 |
| AMERICAN JOURNAL OF EPIDEMIOLOGY | 2 | 0.079 |
| AMERICAN JOURNAL OF OBSTETRICS AND GYNECOLOGY | 2 | 0.079 |
| ANNALS OF THE RHEUMATIC DISEASES | 2 | 0.079 |
| ARCHIVES OF GENERAL PSYCHIATRY | 2 | 0.079 |
| ARCHIVES OF IRANIAN MEDICINE | 2 | 0.079 |
| ARCHIVES OF ORTHOPAEDIC AND TRAUMA SURGERY | 2 | 0.079 |
| ARCHIVES OF RHEUMATOLOGY | 2 | 0.079 |
| ARTHRITIS CARE AND RESEARCH | 2 | 0.079 |
| AUSTRALIAN AND NEW ZEALAND JOURNAL OF PSYCHIATRY | 2 | 0.079 |
| AUSTRALIAN JOURNAL OF PSYCHOLOGY | 2 | 0.079 |
| BEHAVIOR THERAPY | 2 | 0.079 |
| BEHAVIOURAL PHARMACOLOGY | 2 | 0.079 |
| BIOLOGICAL RESEARCH FOR NURSING | 2 | 0.079 |
| BIOMEDICAL RESEARCH INDIA | 2 | 0.079 |
| BMC FAMILY PRACTICE | 2 | 0.079 |
| BRAIN INJURY | 2 | 0.079 |
| BRAIN RESEARCH BULLETIN | 2 | 0.079 |
| BRAIN STIMULATION | 2 | 0.079 |
| BRITISH JOURNAL OF GENERAL PRACTICE | 2 | 0.079 |
| BRITISH JOURNAL OF HEALTH PSYCHOLOGY | 2 | 0.079 |
| BRITISH JOURNAL OF HOSPITAL MEDICINE | 2 | 0.079 |
| CANADIAN JOURNAL OF PUBLIC HEALTH REVUE CANADIENNE DE SANTE PUBLIQUE | 2 | 0.079 |
| CARDIOLOGY IN THE YOUNG | 2 | 0.079 |
| CHINESE JOURNAL OF PHYSIOLOGY | 2 | 0.079 |
| CLINICAL NEUROPHARMACOLOGY | 2 | 0.079 |
| CLINICAL PSYCHOPHARMACOLOGY AND NEUROSCIENCE | 2 | 0.079 |
| CLINICS | 2 | 0.079 |
| COGNITIVE BEHAVIOUR THERAPY | 2 | 0.079 |
| COGNITIVE THERAPY AND RESEARCH | 2 | 0.079 |
| COLLEGIUM ANTROPOLOGICUM | 2 | 0.079 |
| COMPLEMENTARY THERAPIES IN CLINICAL PRACTICE | 2 | 0.079 |
| COMPLEMENTARY THERAPIES IN MEDICINE | 2 | 0.079 |
| COMPTES RENDUS DE L ACADEMIE BULGARE DES SCIENCES | 2 | 0.079 |
| CONTEMPORARY CLINICAL TRIALS | 2 | 0.079 |
| CRANIO THE JOURNAL OF CRANIOMANDIBULAR PRACTICE | 2 | 0.079 |
| CUAJ CANADIAN UROLOGICAL ASSOCIATION JOURNAL | 2 | 0.079 |
| CURRENT PHARMACEUTICAL DESIGN | 2 | 0.079 |
| CURRENT PSYCHIATRY REPORTS | 2 | 0.079 |
| DISABILITY AND HEALTH JOURNAL | 2 | 0.079 |
| EPILEPSY BEHAVIOR | 2 | 0.079 |
| EUROPEAN JOURNAL OF HAEMATOLOGY | 2 | 0.079 |
| EUROPEAN JOURNAL OF NEUROLOGY | 2 | 0.079 |
| EUROPEAN JOURNAL OF NEUROSCIENCE | 2 | 0.079 |
| EUROPEAN JOURNAL OF SPORT SCIENCE | 2 | 0.079 |
| EVOLUTION MEDICINE AND PUBLIC HEALTH | 2 | 0.079 |
| EXPERT OPINION ON INVESTIGATIONAL DRUGS | 2 | 0.079 |
| FRONTIERS IN BIOSCIENCE LANDMARK | 2 | 0.079 |
| GERONTOLOGIST | 2 | 0.079 |
| HARVARD REVIEW OF PSYCHIATRY | 2 | 0.079 |
| HEADACHE QUARTERLY CURRENT TREATMENT AND RESEARCH | 2 | 0.079 |
| HEALTH CARE FOR WOMEN INTERNATIONAL | 2 | 0.079 |
| HEALTHMED | 2 | 0.079 |
| INFANT MENTAL HEALTH JOURNAL | 2 | 0.079 |
| INTERNAL MEDICINE JOURNAL | 2 | 0.079 |
| INTERNATIONAL CLINICAL PSYCHOPHARMACOLOGY | 2 | 0.079 |
| INTERNATIONAL FORUM OF ALLERGY RHINOLOGY | 2 | 0.079 |
| INTERNATIONAL JOURNAL OF NEUROPSYCHOPHARMACOLOGY | 2 | 0.079 |
| INTERNATIONAL JOURNAL OF OSTEOPATHIC MEDICINE | 2 | 0.079 |
| INTERNATIONAL JOURNAL OF PSYCHOPHYSIOLOGY | 2 | 0.079 |
| INTERNATIONAL JOURNAL OF REHABILITATION RESEARCH | 2 | 0.079 |
| INTERNATIONAL REVIEW OF PSYCHIATRY | 2 | 0.079 |
| JAPANESE HEART JOURNAL | 2 | 0.079 |
| JOURNAL OF ANXIETY DISORDERS | 2 | 0.079 |
| JOURNAL OF CEREBRAL BLOOD FLOW AND METABOLISM | 2 | 0.079 |
| JOURNAL OF CHILD AND ADOLESCENT PSYCHOPHARMACOLOGY | 2 | 0.079 |
| JOURNAL OF CLINICAL INVESTIGATION | 2 | 0.079 |
| JOURNAL OF ECT | 2 | 0.079 |
| JOURNAL OF INTERNATIONAL MEDICAL RESEARCH | 2 | 0.079 |
| JOURNAL OF NEUROLOGY | 2 | 0.079 |
| JOURNAL OF NEUROSCIENCE RESEARCH | 2 | 0.079 |
| JOURNAL OF NURSING SCHOLARSHIP | 2 | 0.079 |
| JOURNAL OF OCCUPATIONAL REHABILITATION | 2 | 0.079 |
| JOURNAL OF ORAL AND MAXILLOFACIAL SURGERY | 2 | 0.079 |
| JOURNAL OF ORAL PATHOLOGY MEDICINE | 2 | 0.079 |
| JOURNAL OF PEDIATRIC AND ADOLESCENT GYNECOLOGY | 2 | 0.079 |
| JOURNAL OF PEDIATRIC GASTROENTEROLOGY AND NUTRITION | 2 | 0.079 |
| JOURNAL OF PSYCHOSOMATIC OBSTETRICS GYNECOLOGY | 2 | 0.079 |
| JOURNAL OF REHABILITATION | 2 | 0.079 |
| JOURNAL OF SOCIAL AND CLINICAL PSYCHOLOGY | 2 | 0.079 |
| JOURNAL OF THE AMERICAN ACADEMY OF CHILD AND ADOLESCENT PSYCHIATRY | 2 | 0.079 |
| JOURNAL OF THE AMERICAN BOARD OF FAMILY MEDICINE | 2 | 0.079 |
| JOURNAL OF TRAUMA DISSOCIATION | 2 | 0.079 |
| JOURNAL OF TRAUMATIC STRESS | 2 | 0.079 |
| JOURNAL OF UROLOGY | 2 | 0.079 |
| KLINIK PSIKOFARMAKOLOJI BULTENI BULLETIN OF CLINICAL PSYCHOPHARMACOLOGY | 2 | 0.079 |
| KOREAN JOURNAL OF PAIN | 2 | 0.079 |
| MEDICAL JOURNAL OF AUSTRALIA | 2 | 0.079 |
| METABOLIC BRAIN DISEASE | 2 | 0.079 |
| MOLECULAR NEUROBIOLOGY | 2 | 0.079 |
| NEURAL PLASTICITY | 2 | 0.079 |
| NEUROLOGICAL RESEARCH | 2 | 0.079 |
| NEUROLOGY ASIA | 2 | 0.079 |
| NEURON | 2 | 0.079 |
| NEUROSCIENCE AND BIOBEHAVIORAL REVIEWS | 2 | 0.079 |
| NEUROSCIENTIST | 2 | 0.079 |
| NEUROSIGNALS | 2 | 0.079 |
| ONCOLOGY NURSING FORUM | 2 | 0.079 |
| ORTHOPAEDIC NURSING | 2 | 0.079 |
| PAEDIATRIC ANAESTHESIA | 2 | 0.079 |
| PANMINERVA MEDICA | 2 | 0.079 |
| PATHOLOGIE BIOLOGIE | 2 | 0.079 |
| PEERJ | 2 | 0.079 |
| PEPTIDES | 2 | 0.079 |
| PERCEPTUAL AND MOTOR SKILLS | 2 | 0.079 |
| PHARMACOTHERAPY | 2 | 0.079 |
| PRIMARY HEALTH CARE RESEARCH AND DEVELOPMENT | 2 | 0.079 |
| PROCEEDINGS OF THE NATIONAL ACADEMY OF SCIENCES OF THE UNITED STATES OF AMERICA | 2 | 0.079 |
| PSYCHIATRIC ANNALS | 2 | 0.079 |
| PSYCHIATRIC CLINICS OF NORTH AMERICA | 2 | 0.079 |
| PSYCHOLOGICAL BULLETIN | 2 | 0.079 |
| PSYCHOLOGICAL REPORTS | 2 | 0.079 |
| PSYCHOLOGICAL SERVICES | 2 | 0.079 |
| PSYCHOLOGY OF MUSIC | 2 | 0.079 |
| PSYCHOPHARMACOLOGY BULLETIN | 2 | 0.079 |
| PSYCHOPHYSIOLOGY | 2 | 0.079 |
| PSYCHOTHERAPY RESEARCH | 2 | 0.079 |
| RESEARCH IN GERONTOLOGICAL NURSING | 2 | 0.079 |
| REVISTA DA ASSOCIACAO MEDICA BRASILEIRA | 2 | 0.079 |
| SCANDINAVIAN JOURNAL OF PRIMARY HEALTH CARE | 2 | 0.079 |
| SLEEP MEDICINE | 2 | 0.079 |
| SOCIAL BEHAVIOR AND PERSONALITY | 2 | 0.079 |
| STRESS MEDICINE | 2 | 0.079 |
| SURGICAL ONCOLOGY OXFORD | 2 | 0.079 |
| TRENDS IN NEUROSCIENCES | 2 | 0.079 |
| TRIALS | 2 | 0.079 |
| TURKISH JOURNAL OF GERIATRICS TURK GERIATRI DERGISI | 2 | 0.079 |
| TURKIYE FIZIKSEL TIP VE REHABILITASYON DERGISI TURKISH JOURNAL OF PHYSICAL MEDICINE AND REHABILITATION | 2 | 0.079 |
| TWIN RESEARCH AND HUMAN GENETICS | 2 | 0.079 |
| ZEITSCHRIFT FUR RHEUMATOLOGIE | 2 | 0.079 |
| ACCIDENT ANALYSIS AND PREVENTION | 1 | 0.04 |
| ACTA MEDICA SCANDINAVICA | 1 | 0.04 |
| ACTA NEUROPSYCHIATRICA | 1 | 0.04 |
| ACTA REUMATOLOGICA PORTUGUESA | 1 | 0.04 |
| ADDICTION | 1 | 0.04 |
| ADDICTIVE BEHAVIORS | 1 | 0.04 |
| ADVANCES IN NURSING SCIENCE | 1 | 0.04 |
| AIDS AND BEHAVIOR | 1 | 0.04 |
| ALABAMA JOURNAL OF MEDICAL SCIENCES | 1 | 0.04 |
| AMERICAN JOURNAL OF NURSING | 1 | 0.04 |
| AMERICAN JOURNAL OF ORTHOPSYCHIATRY | 1 | 0.04 |
| AMERICAN JOURNAL OF PHYSICAL MEDICINE REHABILITATION | 1 | 0.04 |
| AMERICAN JOURNAL OF PSYCHOTHERAPY | 1 | 0.04 |
| AMERICAN JOURNAL OF VETERINARY RESEARCH | 1 | 0.04 |
| ANAESTHESIA AND INTENSIVE CARE | 1 | 0.04 |
| ANAIS DA ACADEMIA BRASILEIRA DE CIENCIAS | 1 | 0.04 |
| ANNALS OF CLINICAL PSYCHIATRY | 1 | 0.04 |
| ANNALS OF GENERAL PSYCHIATRY | 1 | 0.04 |
| ANNALS OF PALLIATIVE MEDICINE | 1 | 0.04 |
| ANNALS OF PHARMACOTHERAPY | 1 | 0.04 |
| ANNALS OF SAUDI MEDICINE | 1 | 0.04 |
| ANNALS OF TRANSLATIONAL MEDICINE | 1 | 0.04 |
| ANTONIE VAN LEEUWENHOEK INTERNATIONAL JOURNAL OF GENERAL AND MOLECULAR MICROBIOLOGY | 1 | 0.04 |
| ANXIETY STRESS AND COPING | 1 | 0.04 |
| APPLIED PSYCHOPHYSIOLOGY AND BIOFEEDBACK | 1 | 0.04 |
| ARCHIVES OF BIOLOGICAL SCIENCES | 1 | 0.04 |
| ARCHIVES OF CLINICAL PSYCHIATRY | 1 | 0.04 |
| ARCHIVES OF DERMATOLOGY | 1 | 0.04 |
| ARCHIVES OF ENVIRONMENTAL OCCUPATIONAL HEALTH | 1 | 0.04 |
| ARCHIVES OF GERONTOLOGY AND GERIATRICS | 1 | 0.04 |
| ARCHIVES OF MEDICAL SCIENCE | 1 | 0.04 |
| ARCHIVES OF PHARMACAL RESEARCH | 1 | 0.04 |
| ARCHIVES OF PSYCHIATRIC NURSING | 1 | 0.04 |
| ARCHIVES OF SEXUAL BEHAVIOR | 1 | 0.04 |
| ARCHIVES OF WOMENS MENTAL HEALTH | 1 | 0.04 |
| ARTHRITIS RHEUMATOLOGY | 1 | 0.04 |
| ARTS IN PSYCHOTHERAPY | 1 | 0.04 |
| ARZNEIMITTELFORSCHUNG DRUG RESEARCH | 1 | 0.04 |
| ASIA PACIFIC JOURNAL OF PUBLIC HEALTH | 1 | 0.04 |
| ASIAN BIOMEDICINE | 1 | 0.04 |
| ATHLETIC THERAPY TODAY | 1 | 0.04 |
| AUSTRALIAN DENTAL JOURNAL | 1 | 0.04 |
| AUSTRALIAN FAMILY PHYSICIAN | 1 | 0.04 |
| AUSTRALIAN JOURNAL OF PHYSIOTHERAPY | 1 | 0.04 |
| AUSTRALIAN PSYCHOLOGIST | 1 | 0.04 |
| AUSTRALIAN VETERINARY JOURNAL | 1 | 0.04 |
| BEHAVIOR GENETICS | 1 | 0.04 |
| BEHAVIOR MODIFICATION | 1 | 0.04 |
| BEHAVIORAL NEUROSCIENCE | 1 | 0.04 |
| BEHAVIORAL PSYCHOLOGY PSICOLOGIA CONDUCTUAL | 1 | 0.04 |
| BEHAVIOURAL PSYCHOTHERAPY | 1 | 0.04 |
| BIOLOGY OF REPRODUCTION | 1 | 0.04 |
| BIOMED RESEARCH INTERNATIONAL | 1 | 0.04 |
| BIOMEDICINE PHARMACOTHERAPY | 1 | 0.04 |
| BIOORGANIC MEDICINAL CHEMISTRY LETTERS | 1 | 0.04 |
| BMC CANCER | 1 | 0.04 |
| BMC GASTROENTEROLOGY | 1 | 0.04 |
| BMC HEALTH SERVICES RESEARCH | 1 | 0.04 |
| BMC PREGNANCY AND CHILDBIRTH | 1 | 0.04 |
| BMC WOMENS HEALTH | 1 | 0.04 |
| BMJ SUPPORTIVE PALLIATIVE CARE | 1 | 0.04 |
| BRAZILIAN JOURNAL OF MEDICAL AND BIOLOGICAL RESEARCH | 1 | 0.04 |
| BRITISH JOURNAL OF SPORTS MEDICINE | 1 | 0.04 |
| BRITISH MEDICAL JOURNAL | 1 | 0.04 |
| CANADIAN JOURNAL OF BEHAVIOURAL SCIENCE REVUE CANADIENNE DES SCIENCES DU COMPORTEMENT | 1 | 0.04 |
| CANADIAN VETERINARY JOURNAL REVUE VETERINAIRE CANADIENNE | 1 | 0.04 |
| CANCER | 1 | 0.04 |
| CANCER NURSING | 1 | 0.04 |
| CANCER PRACTICE | 1 | 0.04 |
| CELLULAR AND MOLECULAR NEUROBIOLOGY | 1 | 0.04 |
| CEREAL FOODS WORLD | 1 | 0.04 |
| CEREBROVASCULAR DISEASES | 1 | 0.04 |
| CHAOS | 1 | 0.04 |
| CHILD ABUSE NEGLECT | 1 | 0.04 |
| CHILDREN AND YOUTH SERVICES REVIEW | 1 | 0.04 |
| CHILDRENS HEALTH CARE | 1 | 0.04 |
| CIRCULATION | 1 | 0.04 |
| CLINICA CHIMICA ACTA | 1 | 0.04 |
| CLINICAL AND EXPERIMENTAL PHARMACOLOGY AND PHYSIOLOGY | 1 | 0.04 |
| CLINICAL AND INVESTIGATIVE MEDICINE | 1 | 0.04 |
| CLINICAL CARDIOLOGY | 1 | 0.04 |
| CLINICAL CASE STUDIES | 1 | 0.04 |
| CLINICAL DRUG INVESTIGATION | 1 | 0.04 |
| CLINICAL EEG AND NEUROSCIENCE | 1 | 0.04 |
| CLINICAL GASTROENTEROLOGY AND HEPATOLOGY | 1 | 0.04 |
| CLINICAL GERONTOLOGIST | 1 | 0.04 |
| CLINICAL INTERVENTIONS IN AGING | 1 | 0.04 |
| CLINICAL INVESTIGATOR | 1 | 0.04 |
| CLINICAL MEDICINE | 1 | 0.04 |
| CLINICAL NEUROPSYCHOLOGIST | 1 | 0.04 |
| CLINICAL ORAL INVESTIGATIONS | 1 | 0.04 |
| CLINICAL PHARMACOLOGY THERAPEUTICS | 1 | 0.04 |
| CLINICAL PSYCHOLOGY PSYCHOTHERAPY | 1 | 0.04 |
| CLINICAL REHABILITATION | 1 | 0.04 |
| CNS DRUGS | 1 | 0.04 |
| COGNITIVE AND BEHAVIORAL PRACTICE | 1 | 0.04 |
| COMMUNITY DENTAL HEALTH | 1 | 0.04 |
| CULTURE MEDICINE AND PSYCHIATRY | 1 | 0.04 |
| CURRENT ALZHEIMER RESEARCH | 1 | 0.04 |
| CURRENT DIABETES REPORTS | 1 | 0.04 |
| CURRENT MEDICINAL CHEMISTRY | 1 | 0.04 |
| CURRENT NEUROLOGY AND NEUROSCIENCE REPORTS | 1 | 0.04 |
| CURRENT OPINION IN INVESTIGATIONAL DRUGS | 1 | 0.04 |
| CURRENT OPINION IN NEUROLOGY | 1 | 0.04 |
| CURRENT OPINION IN PHARMACOLOGY | 1 | 0.04 |
| CURRENT OPINION IN PSYCHIATRY | 1 | 0.04 |
| CYTOKINE | 1 | 0.04 |
| DERMATOLOGY | 1 | 0.04 |
| DEVELOPMENTAL MEDICINE AND CHILD NEUROLOGY | 1 | 0.04 |
| DIABETES CARE | 1 | 0.04 |
| DIABETES VASCULAR DISEASE RESEARCH | 1 | 0.04 |
| DIABETIC MEDICINE | 1 | 0.04 |
| DIGESTIVE DISEASES AND SCIENCES | 1 | 0.04 |
| DISEASES OF THE COLON RECTUM | 1 | 0.04 |
| DRUG DEVELOPMENT RESEARCH | 1 | 0.04 |
| ENDOKRYNOLOGIA POLSKA | 1 | 0.04 |
| ENVIRONMENTAL HEALTH AND PREVENTIVE MEDICINE | 1 | 0.04 |
| ENVIRONMENTAL RESEARCH LETTERS | 1 | 0.04 |
| EPIDEMIOLOGY AND PSYCHIATRIC SCIENCES | 1 | 0.04 |
| EPILEPSY RESEARCH | 1 | 0.04 |
| EUROPEAN ARCHIVES OF PSYCHIATRY AND CLINICAL NEUROSCIENCE | 1 | 0.04 |
| EUROPEAN CHILD ADOLESCENT PSYCHIATRY | 1 | 0.04 |
| EUROPEAN JOURNAL OF CARDIOVASCULAR NURSING | 1 | 0.04 |
| EUROPEAN JOURNAL OF CARDIOVASCULAR PREVENTION REHABILITATION | 1 | 0.04 |
| EUROPEAN JOURNAL OF GASTROENTEROLOGY HEPATOLOGY | 1 | 0.04 |
| EUROPEAN JOURNAL OF HUMAN GENETICS | 1 | 0.04 |
| EUROPEAN JOURNAL OF ONCOLOGY NURSING | 1 | 0.04 |
| EUROPEAN JOURNAL OF PAIN LONDON | 1 | 0.04 |
| EUROPEAN JOURNAL OF PHYSICAL AND REHABILITATION MEDICINE | 1 | 0.04 |
| EUROPEAN JOURNAL OF TRAUMA AND EMERGENCY SURGERY | 1 | 0.04 |
| EUROPEAN NEUROPSYCHOPHARMACOLOGY | 1 | 0.04 |
| EUROPEAN RESPIRATORY JOURNAL | 1 | 0.04 |
| EUROPEAN REVIEW OF APPLIED PSYCHOLOGY REVUE EUROPEENNE DE PSYCHOLOGIE APPLIQUEE | 1 | 0.04 |
| EXPERIMENTAL AGING RESEARCH | 1 | 0.04 |
| EXPERIMENTAL AND CLINICAL ENDOCRINOLOGY DIABETES | 1 | 0.04 |
| EXPERIMENTAL AND THERAPEUTIC MEDICINE | 1 | 0.04 |
| EXPERT OPINION ON PHARMACOTHERAPY | 1 | 0.04 |
| EXPERT OPINION ON THERAPEUTIC TARGETS | 1 | 0.04 |
| EXPERT REVIEW OF CLINICAL PHARMACOLOGY | 1 | 0.04 |
| EXPERT REVIEW OF NEUROTHERAPEUTICS | 1 | 0.04 |
| FAMILY COMMUNITY HEALTH | 1 | 0.04 |
| FAMILY PRACTICE | 1 | 0.04 |
| FRONTIERS IN CELLULAR NEUROSCIENCE | 1 | 0.04 |
| FRONTIERS IN MOLECULAR NEUROSCIENCE | 1 | 0.04 |
| FRONTIERS IN PHARMACOLOGY | 1 | 0.04 |
| GENES BRAIN AND BEHAVIOR | 1 | 0.04 |
| GERIATRIC NURSING | 1 | 0.04 |
| GERIATRICS | 1 | 0.04 |
| GINEKOLOGIA POLSKA | 1 | 0.04 |
| GYNECOLOGICAL ENDOCRINOLOGY | 1 | 0.04 |
| HEART LUNG AND CIRCULATION | 1 | 0.04 |
| HEMODIALYSIS INTERNATIONAL | 1 | 0.04 |
| HOLISTIC NURSING PRACTICE | 1 | 0.04 |
| HUMAN GENETICS | 1 | 0.04 |
| IDRUGS | 1 | 0.04 |
| IEEE ACCESS | 1 | 0.04 |
| IEEE PULSE | 1 | 0.04 |
| INDIAN JOURNAL OF PHARMACEUTICAL EDUCATION AND RESEARCH | 1 | 0.04 |
| INFLAMMATORY BOWEL DISEASES | 1 | 0.04 |
| INTENSIVE CARE MEDICINE | 1 | 0.04 |
| INTERNAL MEDICINE | 1 | 0.04 |
| INTERNATIONAL JOURNAL OF CARDIOLOGY | 1 | 0.04 |
| INTERNATIONAL JOURNAL OF EATING DISORDERS | 1 | 0.04 |
| INTERNATIONAL JOURNAL OF ENVIRONMENTAL RESEARCH AND PUBLIC HEALTH | 1 | 0.04 |
| INTERNATIONAL JOURNAL OF IMPOTENCE RESEARCH | 1 | 0.04 |
| INTERNATIONAL JOURNAL OF NURSING PRACTICE | 1 | 0.04 |
| INTERNATIONAL JOURNAL OF PROSTHODONTICS | 1 | 0.04 |
| INTERNATIONAL JOURNAL OF RADIATION ONCOLOGY BIOLOGY PHYSICS | 1 | 0.04 |
| INTERNATIONAL JOURNAL OF SEXUAL HEALTH | 1 | 0.04 |
| INTERNATIONAL JOURNAL OF SURGERY | 1 | 0.04 |
| INTERNATIONAL NEUROUROLOGY JOURNAL | 1 | 0.04 |
| INTERNATIONAL ORTHOPAEDICS | 1 | 0.04 |
| INTERNATIONAL UROLOGY AND NEPHROLOGY | 1 | 0.04 |
| IRANIAN JOURNAL OF PUBLIC HEALTH | 1 | 0.04 |
| IRISH JOURNAL OF MEDICAL SCIENCE | 1 | 0.04 |
| ISRAEL JOURNAL OF PSYCHIATRY AND RELATED SCIENCES | 1 | 0.04 |
| JNCI JOURNAL OF THE NATIONAL CANCER INSTITUTE | 1 | 0.04 |
| JNP JOURNAL FOR NURSE PRACTITIONERS | 1 | 0.04 |
| JOURNAL OF ADOLESCENT HEALTH | 1 | 0.04 |
| JOURNAL OF AGROMEDICINE | 1 | 0.04 |
| JOURNAL OF APPLIED SOCIAL PSYCHOLOGY | 1 | 0.04 |
| JOURNAL OF APPLIED TOXICOLOGY | 1 | 0.04 |
| JOURNAL OF BONE AND JOINT SURGERY AMERICAN VOLUME | 1 | 0.04 |
| JOURNAL OF CARDIOVASCULAR NURSING | 1 | 0.04 |
| JOURNAL OF CLINICAL AND EXPERIMENTAL NEUROPSYCHOLOGY | 1 | 0.04 |
| JOURNAL OF CLINICAL CHILD AND ADOLESCENT PSYCHOLOGY | 1 | 0.04 |
| JOURNAL OF CLINICAL NURSING | 1 | 0.04 |
| JOURNAL OF CLINICAL ONCOLOGY | 1 | 0.04 |
| JOURNAL OF CLINICAL SLEEP MEDICINE | 1 | 0.04 |
| JOURNAL OF CONTEXTUAL BEHAVIORAL SCIENCE | 1 | 0.04 |
| JOURNAL OF DENTISTRY | 1 | 0.04 |
| JOURNAL OF ELDER ABUSE NEGLECT | 1 | 0.04 |
| JOURNAL OF EMERGENCY MEDICINE | 1 | 0.04 |
| JOURNAL OF EMERGENCY NURSING | 1 | 0.04 |
| JOURNAL OF ETHNOPHARMACOLOGY | 1 | 0.04 |
| JOURNAL OF EVOLUTIONARY BIOCHEMISTRY AND PHYSIOLOGY | 1 | 0.04 |
| JOURNAL OF EXPERIMENTAL BIOLOGY | 1 | 0.04 |
| JOURNAL OF FAMILY PSYCHOLOGY | 1 | 0.04 |
| JOURNAL OF FAMILY VIOLENCE | 1 | 0.04 |
| JOURNAL OF GERIATRIC ONCOLOGY | 1 | 0.04 |
| JOURNAL OF GERIATRIC PSYCHIATRY AND NEUROLOGY | 1 | 0.04 |
| JOURNAL OF HAND SURGERY AMERICAN VOLUME | 1 | 0.04 |
| JOURNAL OF HEALTH AND SOCIAL BEHAVIOR | 1 | 0.04 |
| JOURNAL OF MARRIAGE AND THE FAMILY | 1 | 0.04 |
| JOURNAL OF MEDICAL INTERNET RESEARCH | 1 | 0.04 |
| JOURNAL OF MEDICINAL CHEMISTRY | 1 | 0.04 |
| JOURNAL OF MENTAL HEALTH POLICY AND ECONOMICS | 1 | 0.04 |
| JOURNAL OF MOLECULAR NEUROSCIENCE | 1 | 0.04 |
| JOURNAL OF NEUROENDOCRINOLOGY | 1 | 0.04 |
| JOURNAL OF NEUROLOGICAL AND ORTHOPAEDIC MEDICINE AND SURGERY | 1 | 0.04 |
| JOURNAL OF NEUROLOGICAL SCIENCES TURKISH | 1 | 0.04 |
| JOURNAL OF NEUROPSYCHIATRY AND CLINICAL NEUROSCIENCES | 1 | 0.04 |
| JOURNAL OF NEUROSURGERY SPINE | 1 | 0.04 |
| JOURNAL OF NEUROSURGICAL SCIENCES | 1 | 0.04 |
| JOURNAL OF NUTRITION HEALTH AGING | 1 | 0.04 |
| JOURNAL OF OBSTETRICS AND GYNAECOLOGY | 1 | 0.04 |
| JOURNAL OF ORTHOMOLECULAR PSYCHIATRY | 1 | 0.04 |
| JOURNAL OF ORTHOPAEDIC SCIENCE | 1 | 0.04 |
| JOURNAL OF ORTHOPAEDIC SPORTS PHYSICAL THERAPY | 1 | 0.04 |
| JOURNAL OF OTOLARYNGOLOGY HEAD NECK SURGERY | 1 | 0.04 |
| JOURNAL OF PALLIATIVE CARE | 1 | 0.04 |
| JOURNAL OF PERSONALITY | 1 | 0.04 |
| JOURNAL OF PHARMACY AND PHARMACOLOGY | 1 | 0.04 |
| JOURNAL OF PROSTHETIC DENTISTRY | 1 | 0.04 |
| JOURNAL OF PSYCHIATRIC PRACTICE | 1 | 0.04 |
| JOURNAL OF PSYCHIATRIC TREATMENT AND EVALUATION | 1 | 0.04 |
| JOURNAL OF PSYCHOPATHOLOGY AND BEHAVIORAL ASSESSMENT | 1 | 0.04 |
| JOURNAL OF PSYCHOSOCIAL NURSING AND MENTAL HEALTH SERVICES | 1 | 0.04 |
| JOURNAL OF PSYCHOSOCIAL ONCOLOGY | 1 | 0.04 |
| JOURNAL OF PSYCHOSOMATIC OBSTETRICS AND GYNECOLOGY | 1 | 0.04 |
| JOURNAL OF RATIONAL EMOTIVE AND COGNITIVE BEHAVIOR THERAPY | 1 | 0.04 |
| JOURNAL OF REPRODUCTIVE AND INFANT PSYCHOLOGY | 1 | 0.04 |
| JOURNAL OF REPRODUCTIVE MEDICINE | 1 | 0.04 |
| JOURNAL OF SEX RESEARCH | 1 | 0.04 |
| JOURNAL OF SEXUAL MEDICINE | 1 | 0.04 |
| JOURNAL OF STUDIES ON ALCOHOL AND DRUGS | 1 | 0.04 |
| JOURNAL OF SUBSTANCE ABUSE TREATMENT | 1 | 0.04 |
| JOURNAL OF THE AMERICAN ASSOCIATION OF NURSE PRACTITIONERS | 1 | 0.04 |
| JOURNAL OF THE AMERICAN DENTAL ASSOCIATION | 1 | 0.04 |
| JOURNAL OF THE AMERICAN PODIATRIC MEDICAL ASSOCIATION | 1 | 0.04 |
| JOURNAL OF THE AMERICAN SOCIETY FOR MASS SPECTROMETRY | 1 | 0.04 |
| JOURNAL OF THE FORMOSAN MEDICAL ASSOCIATION | 1 | 0.04 |
| JOURNAL OF THE INTERNATIONAL NEUROPSYCHOLOGICAL SOCIETY | 1 | 0.04 |
| JOURNAL OF THE NATIONAL CANCER INSTITUTE | 1 | 0.04 |
| JOURNAL OF THE NATIONAL MEDICAL ASSOCIATION | 1 | 0.04 |
| JOURNAL OF THE NEUROLOGICAL SCIENCES | 1 | 0.04 |
| JOURNAL OF THORACIC DISEASE | 1 | 0.04 |
| JOURNAL OF VISUAL IMPAIRMENT BLINDNESS | 1 | 0.04 |
| JOURNAL OF WOMEN AGING | 1 | 0.04 |
| JOURNAL OF ZOO AND WILDLIFE MEDICINE | 1 | 0.04 |
| JOURNALS OF GERONTOLOGY SERIES B PSYCHOLOGICAL SCIENCES AND SOCIAL SCIENCES | 1 | 0.04 |
| JOVE JOURNAL OF VISUALIZED EXPERIMENTS | 1 | 0.04 |
| KEXUE TONGBAO | 1 | 0.04 |
| KUWAIT MEDICAL JOURNAL | 1 | 0.04 |
| LAB ANIMAL | 1 | 0.04 |
| LANCET PSYCHIATRY | 1 | 0.04 |
| LUNG CANCER | 1 | 0.04 |
| LUPUS | 1 | 0.04 |
| MAGNESIUM RESEARCH | 1 | 0.04 |
| MATURITAS | 1 | 0.04 |
| MEDIATORS OF INFLAMMATION | 1 | 0.04 |
| MEDICAL PROBLEMS OF PERFORMING ARTISTS | 1 | 0.04 |
| MEDICAL SCIENCE MONITOR | 1 | 0.04 |
| MEDICINA LITHUANIA | 1 | 0.04 |
| MEDICINA ORAL PATOLOGIA ORAL Y CIRUGIA BUCAL | 1 | 0.04 |
| MEDICINE AND SCIENCE IN SPORTS AND EXERCISE | 1 | 0.04 |
| MILITARY MEDICINE | 1 | 0.04 |
| MOLECULAR BIOSYSTEMS | 1 | 0.04 |
| MOLECULAR MEDICINE | 1 | 0.04 |
| MOLECULAR MEDICINE REPORTS | 1 | 0.04 |
| MULTIPLE SCLEROSIS | 1 | 0.04 |
| MUSCULOSKELETAL SCIENCE AND PRACTICE | 1 | 0.04 |
| NATURE REVIEWS NEUROLOGY | 1 | 0.04 |
| NATURE REVIEWS RHEUMATOLOGY | 1 | 0.04 |
| NAUNYN SCHMIEDEBERGS ARCHIVES OF PHARMACOLOGY | 1 | 0.04 |
| NEUROBIOLOGY OF DISEASE | 1 | 0.04 |
| NEUROCASE | 1 | 0.04 |
| NEUROENDOCRINOLOGY | 1 | 0.04 |
| NEUROENDOCRINOLOGY LETTERS | 1 | 0.04 |
| NEUROEPIDEMIOLOGY | 1 | 0.04 |
| NEUROGASTROENTEROLOGY AND MOTILITY | 1 | 0.04 |
| NEUROIMAGE | 1 | 0.04 |
| NEUROLOGIA I NEUROCHIRURGIA POLSKA | 1 | 0.04 |
| NEUROMODULATION | 1 | 0.04 |
| NEUROMOLECULAR MEDICINE | 1 | 0.04 |
| NEUROPHYSIOLOGIE CLINIQUE CLINICAL NEUROPHYSIOLOGY | 1 | 0.04 |
| NEUROPSYCHOLOGY | 1 | 0.04 |
| NEUROREHABILITATION | 1 | 0.04 |
| NEUROSCIENCE RESEARCH | 1 | 0.04 |
| NEUROTHERAPEUTICS | 1 | 0.04 |
| NEUROUROLOGY AND URODYNAMICS | 1 | 0.04 |
| NEW ZEALAND MEDICAL JOURNAL | 1 | 0.04 |
| NICOTINE TOBACCO RESEARCH | 1 | 0.04 |
| NMR IN BIOMEDICINE | 1 | 0.04 |
| NOROPSIKIYATRI ARSIVI ARCHIVES OF NEUROPSYCHIATRY | 1 | 0.04 |
| NURSING CLINICS OF NORTH AMERICA | 1 | 0.04 |
| OBSTETRICS AND GYNECOLOGY | 1 | 0.04 |
| OBSTETRICS AND GYNECOLOGY CLINICS OF NORTH AMERICA | 1 | 0.04 |
| OCCUPATIONAL AND ENVIRONMENTAL MEDICINE | 1 | 0.04 |
| ONCOTARGET | 1 | 0.04 |
| ONCOTARGETS AND THERAPY | 1 | 0.04 |
| ORAL SURGERY ORAL MEDICINE ORAL PATHOLOGY ORAL RADIOLOGY AND ENDODONTICS | 1 | 0.04 |
| ORAL SURGERY ORAL MEDICINE ORAL PATHOLOGY ORAL RADIOLOGY AND ENDODONTOLOGY | 1 | 0.04 |
| OSTEOARTHRITIS AND CARTILAGE | 1 | 0.04 |
| PAIN AND THE BRAIN | 1 | 0.04 |
| PALLIATIVE MEDICINE | 1 | 0.04 |
| PATIENT EDUCATION AND COUNSELING | 1 | 0.04 |
| PATIENT PATIENT CENTERED OUTCOMES RESEARCH | 1 | 0.04 |
| PATIENT PREFERENCE AND ADHERENCE | 1 | 0.04 |
| PEDIATRIC BLOOD CANCER | 1 | 0.04 |
| PERSONALITY AND INDIVIDUAL DIFFERENCES | 1 | 0.04 |
| PERSPECTIVES IN PSYCHIATRIC CARE | 1 | 0.04 |
| PFLUGERS ARCHIV EUROPEAN JOURNAL OF PHYSIOLOGY | 1 | 0.04 |
| PHARMACEUTICAL RESEARCH | 1 | 0.04 |
| PHARMACOEPIDEMIOLOGY AND DRUG SAFETY | 1 | 0.04 |
| PHARMACOGENETICS AND GENOMICS | 1 | 0.04 |
| PHARMACOLOGICAL REPORTS | 1 | 0.04 |
| PHARMACOLOGICAL REVIEWS | 1 | 0.04 |
| PHARMACOLOGY TOXICOLOGY | 1 | 0.04 |
| PHYSICAL MEDICINE AND REHABILITATION CLINICS OF NORTH AMERICA | 1 | 0.04 |
| PHYSIKALISCHE MEDIZIN REHABILITATIONSMEDIZIN KURORTMEDIZIN | 1 | 0.04 |
| PLASTIC AND RECONSTRUCTIVE SURGERY | 1 | 0.04 |
| PLOS GENETICS | 1 | 0.04 |
| PLOS MEDICINE | 1 | 0.04 |
| PRACTITIONER | 1 | 0.04 |
| PRIMARY CARE | 1 | 0.04 |
| PRIMARY CARE COMMUNITY PSYCHIATRY | 1 | 0.04 |
| PRIMARY CARE PSYCHIATRY | 1 | 0.04 |
| PROCEEDINGS OF THE ROYAL SOCIETY B BIOLOGICAL SCIENCES | 1 | 0.04 |
| PROFESSIONAL PSYCHOLOGY RESEARCH AND PRACTICE | 1 | 0.04 |
| PSYCHIATRIA CLINICA | 1 | 0.04 |
| PSYCHIATRIC QUARTERLY | 1 | 0.04 |
| PSYCHIATRY INTERPERSONAL AND BIOLOGICAL PROCESSES | 1 | 0.04 |
| PSYCHIATRY RESEARCH NEUROIMAGING | 1 | 0.04 |
| PSYCHOLOGICAL RECORD | 1 | 0.04 |
| PSYCHOLOGICAL SCIENCE | 1 | 0.04 |
| PSYCHOLOGY AND AGING | 1 | 0.04 |
| PSYCHOTHERAPIE PSYCHOSOMATIK MEDIZINISCHE PSYCHOLOGIE | 1 | 0.04 |
| PUBLIC HEALTH | 1 | 0.04 |
| QJM AN INTERNATIONAL JOURNAL OF MEDICINE | 1 | 0.04 |
| REHABILITATION COUNSELING BULLETIN | 1 | 0.04 |
| REPRODUCTIVE HEALTH | 1 | 0.04 |
| RESEARCH AND THEORY FOR NURSING PRACTICE | 1 | 0.04 |
| RESEARCH ON AGING | 1 | 0.04 |
| REVIEWS IN CARDIOVASCULAR MEDICINE | 1 | 0.04 |
| REVIEWS IN THE NEUROSCIENCES | 1 | 0.04 |
| REVISTA DA ESCOLA DE ENFERMAGEM DA USP | 1 | 0.04 |
| REVISTA DE PSIQUIATRIA CLINICA | 1 | 0.04 |
| REVISTA LATINO AMERICANA DE ENFERMAGEM | 1 | 0.04 |
| REVUE NEUROLOGIQUE | 1 | 0.04 |
| RHEUMATIC DISEASE CLINICS OF NORTH AMERICA | 1 | 0.04 |
| SCANDINAVIAN JOURNAL OF CARING SCIENCES | 1 | 0.04 |
| SCANDINAVIAN JOURNAL OF PSYCHOLOGY | 1 | 0.04 |
| SCANDINAVIAN JOURNAL OF PUBLIC HEALTH | 1 | 0.04 |
| SCANDINAVIAN JOURNAL OF REHABILITATION MEDICINE | 1 | 0.04 |
| SCIENCE SIGNALING | 1 | 0.04 |
| SCIENTIFIC WORLD JOURNAL | 1 | 0.04 |
| SEMINARS IN DIALYSIS | 1 | 0.04 |
| SLEEP MEDICINE REVIEWS | 1 | 0.04 |
| SOCIAL PSYCHOLOGY QUARTERLY | 1 | 0.04 |
| SOCIAL SCIENCE MEDICINE | 1 | 0.04 |
| SOCIAL WORK IN HEALTH CARE | 1 | 0.04 |
| SPANISH JOURNAL OF PSYCHOLOGY | 1 | 0.04 |
| SPRINGERPLUS | 1 | 0.04 |
| STRESS AND HEALTH | 1 | 0.04 |
| STROKE | 1 | 0.04 |
| SUICIDE AND LIFE THREATENING BEHAVIOR | 1 | 0.04 |
| SWISS MEDICAL WEEKLY | 1 | 0.04 |
| TELEMEDICINE JOURNAL AND E HEALTH | 1 | 0.04 |
| TEXAS HEART INSTITUTE JOURNAL | 1 | 0.04 |
| TOHOKU JOURNAL OF EXPERIMENTAL MEDICINE | 1 | 0.04 |
| TOXICOLOGY AND APPLIED PHARMACOLOGY | 1 | 0.04 |
| TRANSLATIONAL PSYCHIATRY | 1 | 0.04 |
| UNDERSEA BIOMEDICAL RESEARCH | 1 | 0.04 |
| UPSALA JOURNAL OF MEDICAL SCIENCES | 1 | 0.04 |
| UROLOGY | 1 | 0.04 |
| WEST INDIAN MEDICAL JOURNAL | 1 | 0.04 |
| WIENER KLINISCHE WOCHENSCHRIFT | 1 | 0.04 |
| WOMEN HEALTH | 1 | 0.04 |
| WOMENS HEALTH ISSUES | 1 | 0.04 |
| WORK A JOURNAL OF PREVENTION ASSESSMENT REHABILITATION | 1 | 0.04 |
| WORLDVIEWS ON EVIDENCE BASED NURSING | 1 | 0.04 |
| ZDRAVNISKI VESTNIK SLOVENIAN MEDICAL JOURNAL | 1 | 0.04 |
| ZDRAVSTVENO VARSTVO | 1 | 0.04 |
