# Supplementary material for: Bibliometric Study of the Comorbidity of Pain and Depression Research
Source: Neural Plast. 2019 Oct 23;2019:1657498. doi: 10.1155/2019/1657498 (PMC6854239; doi:10.1155/2019/1657498)
Supplement: Supplementary 4 — Supplementary Table 2: Raw data on countries/territories involved in pain and depression publications. [file 1657498.f4.doc]

**Supplementary Table 2. Raw data on countries/territories involved in pain and depression publications.**

| **Countries/Territories** | **Records** | **% of 2519** |
| --- | --- | --- |
| USA | 1105 | 43.867 |
| ENGLAND | 178 | 7.066 |
| CANADA | 156 | 6.193 |
| GERMANY | 139 | 5.518 |
| ITALY | 114 | 4.526 |
| AUSTRALIA | 113 | 4.486 |
| PEOPLES R CHINA | 113 | 4.486 |
| TURKEY | 106 | 4.208 |
| SPAIN | 89 | 3.533 |
| NETHERLANDS | 86 | 3.414 |
| SWEDEN | 72 | 2.858 |
| JAPAN | 68 | 2.699 |
| BRAZIL | 64 | 2.541 |
| TAIWAN | 63 | 2.501 |
| DENMARK | 52 | 2.064 |
| SOUTH KOREA | 51 | 2.025 |
| NORWAY | 43 | 1.707 |
| FRANCE | 42 | 1.667 |
| SWITZERLAND | 39 | 1.548 |
| FINLAND | 38 | 1.509 |
| BELGIUM | 29 | 1.151 |
| ISRAEL | 21 | 0.834 |
| AUSTRIA | 18 | 0.715 |
| IRELAND | 17 | 0.675 |
| NEW ZEALAND | 16 | 0.635 |
| IRAN | 15 | 0.595 |
| SCOTLAND | 14 | 0.556 |
| INDIA | 13 | 0.516 |
| PORTUGAL | 13 | 0.516 |
| GREECE | 11 | 0.437 |
| HUNGARY | 11 | 0.437 |
| POLAND | 11 | 0.437 |
| MEXICO | 10 | 0.397 |
| CROATIA | 9 | 0.357 |
| SINGAPORE | 9 | 0.357 |
| FED REP GER | 8 | 0.318 |
| RUSSIA | 7 | 0.278 |
| WALES | 7 | 0.278 |
| SOUTH AFRICA | 6 | 0.238 |
| THAILAND | 6 | 0.238 |
| INDONESIA | 5 | 0.198 |
| PAKISTAN | 5 | 0.198 |
| BULGARIA | 4 | 0.159 |
| CZECH REPUBLIC | 4 | 0.159 |
| ARGENTINA | 3 | 0.119 |
| CHILE | 3 | 0.119 |
| COLOMBIA | 3 | 0.119 |
| EGYPT | 3 | 0.119 |
| ICELAND | 3 | 0.119 |
| JORDAN | 3 | 0.119 |
| MALAYSIA | 3 | 0.119 |
| NIGERIA | 3 | 0.119 |
| SLOVENIA | 3 | 0.119 |
| UGANDA | 3 | 0.119 |
| CUBA | 2 | 0.079 |
| ETHIOPIA | 2 | 0.079 |
| LEBANON | 2 | 0.079 |
| LITHUANIA | 2 | 0.079 |
| LUXEMBOURG | 2 | 0.079 |
| QATAR | 2 | 0.079 |
| UKRAINE | 2 | 0.079 |
| VENEZUELA | 2 | 0.079 |
| ESTONIA | 1 | 0.04 |
| HONG KONG | 1 | 0.04 |
| KOSOVO | 1 | 0.04 |
| KUWAIT | 1 | 0.04 |
| MALTA | 1 | 0.04 |
| NORTH IRELAND | 1 | 0.04 |
| PERU | 1 | 0.04 |
| REP OF GEORGIA | 1 | 0.04 |
| ROMANIA | 1 | 0.04 |
| SERBIA | 1 | 0.04 |
| SLOVAKIA | 1 | 0.04 |
| U ARAB EMIRATES | 1 | 0.04 |
| USSR | 1 | 0.04 |
