# Supplementary material for: Bibliometric Study of the Comorbidity of Pain and Depression Research
Source: Neural Plast. 2019 Oct 23;2019:1657498. doi: 10.1155/2019/1657498 (PMC6854239; doi:10.1155/2019/1657498)
Supplement: Supplementary 5 — Supplementary Table 3: Raw data on institutions involved in pain and depression publications. [file 1657498.f5.doc]

**Supplementary Table 3. Raw data on institutions involved in pain and depression publications.**

| **Institutions** | **records** | **% of 2519** |
| --- | --- | --- |
| UNIVERSITY OF CALIFORNIA SYSTEM | 108 | 4.287 |
| HARVARD UNIVERSITY | 95 | 3.771 |
| UNIVERSITY OF WASHINGTON | 85 | 3.374 |
| UNIVERSITY OF WASHINGTON SEATTLE | 85 | 3.374 |
| ELI LILLY | 68 | 2.699 |
| VA BOSTON HEALTHCARE SYSTEM | 64 | 2.541 |
| UNIVERSITY OF LONDON | 60 | 2.382 |
| UNIVERSITY OF TEXAS SYSTEM | 56 | 2.223 |
| INDIANA UNIVERSITY SYSTEM | 55 | 2.183 |
| INDIANA UNIVERSITY PURDUE UNIVERSITY INDIANAPOLIS | 54 | 2.144 |
| PENNSYLVANIA COMMONWEALTH SYSTEM OF HIGHER EDUCATION PCSHE | 45 | 1.786 |
| UNIVERSITY OF MICHIGAN | 45 | 1.786 |
| UNIVERSITY OF MICHIGAN SYSTEM | 45 | 1.786 |
| UNIVERSITY OF CALIFORNIA SAN DIEGO | 41 | 1.628 |
| DUKE UNIVERSITY | 38 | 1.509 |
| MASSACHUSETTS GENERAL HOSPITAL | 38 | 1.509 |
| UNIVERSITY OF TORONTO | 38 | 1.509 |
| UNIVERSITY OF PITTSBURGH | 37 | 1.469 |
| JOHNS HOPKINS UNIVERSITY | 36 | 1.429 |
| UNIVERSITY OF CALIFORNIA LOS ANGELES | 36 | 1.429 |
| LEIDEN UNIVERSITY | 35 | 1.389 |
| KINGS COLLEGE LONDON | 34 | 1.35 |
| REGENSTRIEF INSTITUTE INC | 34 | 1.35 |
| STATE UNIVERSITY SYSTEM OF FLORIDA | 32 | 1.27 |
| VIRGINIA COMMONWEALTH UNIVERSITY | 32 | 1.27 |
| STANFORD UNIVERSITY | 30 | 1.191 |
| VRIJE UNIVERSITEIT AMSTERDAM | 30 | 1.191 |
| NATIONAL INSTITUTES OF HEALTH NIH USA | 29 | 1.151 |
| STATE UNIVERSITY OF NEW YORK SUNY SYSTEM | 29 | 1.151 |
| UNIVERSITY OF SYDNEY | 29 | 1.151 |
| US DEPARTMENT OF VETERAN AFFAIRS | 29 | 1.151 |
| UNIVERSITY OF PENNSYLVANIA | 28 | 1.112 |
| KAROLINSKA INSTITUTET | 27 | 1.072 |
| UNIVERSITY OF CALIFORNIA SAN FRANCISCO | 26 | 1.032 |
| UNIVERSITY OF CINCINNATI | 26 | 1.032 |
| UNIVERSITY OF COPENHAGEN | 26 | 1.032 |
| UNIVERSITY OF ROCHESTER | 26 | 1.032 |
| YALE UNIVERSITY | 26 | 1.032 |
| COLUMBIA UNIVERSITY | 25 | 0.992 |
| NATIONAL YANG MING UNIVERSITY | 25 | 0.992 |
| UNIVERSITY OF NORTH CAROLINA | 23 | 0.913 |
| CHANG GUNG UNIVERSITY | 22 | 0.873 |
| TAIPEI VETERANS GENERAL HOSPITAL | 22 | 0.873 |
| UNIVERSIDADE DE SAO PAULO | 22 | 0.873 |
| VETERANS HEALTH ADMINISTRATION VHA | 22 | 0.873 |
| CHANG GUNG MEMORIAL HOSPITAL | 21 | 0.834 |
| EMORY UNIVERSITY | 21 | 0.834 |
| CIBER CENTRO DE INVESTIGACION BIOMEDICA EN RED | 20 | 0.794 |
| UNIVERSITY COLLEGE LONDON | 20 | 0.794 |
| UNIVERSITY OF NORTH CAROLINA CHAPEL HILL | 20 | 0.794 |
| UNIVERSITY OF ALABAMA SYSTEM | 19 | 0.754 |
| UNIVERSITY OF OSLO | 19 | 0.754 |
| UNIVERSITY OF PADUA | 19 | 0.754 |
| VANDERBILT UNIVERSITY | 19 | 0.754 |
| UNIVERSITY OF TEXAS SOUTHWESTERN MEDICAL CENTER DALLAS | 18 | 0.715 |
| OREGON HEALTH SCIENCE UNIVERSITY | 17 | 0.675 |
| UNIVERSIDAD REY JUAN CARLOS | 17 | 0.675 |
| UNIVERSITY OF BERGEN | 17 | 0.675 |
| UNIVERSITY OF CALGARY | 17 | 0.675 |
| UNIVERSITY OF FLORIDA | 17 | 0.675 |
| CIBERSAM | 16 | 0.635 |
| DAVID GEFFEN SCHOOL OF MEDICINE AT UCLA | 16 | 0.635 |
| IMPERIAL COLLEGE LONDON | 16 | 0.635 |
| KU LEUVEN | 16 | 0.635 |
| MAYO CLINIC | 16 | 0.635 |
| UNIVERSITY OF TURIN | 16 | 0.635 |
| BAYLOR COLLEGE OF MEDICINE | 15 | 0.595 |
| BROWN UNIVERSITY | 15 | 0.595 |
| CALIFORNIA STATE UNIVERSITY SYSTEM | 15 | 0.595 |
| FRIEDRICH SCHILLER UNIVERSITY OF JENA | 15 | 0.595 |
| NEW YORK UNIVERSITY | 15 | 0.595 |
| UNIVERSITY OF MANCHESTER | 15 | 0.595 |
| UPPSALA UNIVERSITY | 15 | 0.595 |
| ASSISTANCE PUBLIQUE HOPITAUX PARIS APHP | 14 | 0.556 |
| CHINESE ACADEMY OF SCIENCES | 14 | 0.556 |
| CORNELL UNIVERSITY | 14 | 0.556 |
| MAASTRICHT UNIVERSITY | 14 | 0.556 |
| MCGILL UNIVERSITY | 14 | 0.556 |
| RUSH UNIVERSITY | 14 | 0.556 |
| SAPIENZA UNIVERSITY ROME | 14 | 0.556 |
| UNIVERSITY OF GRANADA | 14 | 0.556 |
| UNIVERSITY OF MIAMI | 14 | 0.556 |
| UNIVERSITY OF OTTAWA | 14 | 0.556 |
| UNIVERSITY OF TEXAS HEALTH SCIENCE CENTER HOUSTON | 14 | 0.556 |
| UNIVERSITY SYSTEM OF GEORGIA | 14 | 0.556 |
| WASHINGTON UNIVERSITY WUSTL | 14 | 0.556 |
| YESHIVA UNIVERSITY | 14 | 0.556 |
| AALBORG UNIVERSITY | 13 | 0.516 |
| CATHOLIC UNIVERSITY OF KOREA | 13 | 0.516 |
| NORWEGIAN UNIVERSITY OF SCIENCE TECHNOLOGY NTNU | 13 | 0.516 |
| RUPRECHT KARLS UNIVERSITY HEIDELBERG | 13 | 0.516 |
| RUTGERS STATE UNIVERSITY NEW BRUNSWICK | 13 | 0.516 |
| SEOUL NATIONAL UNIVERSITY | 13 | 0.516 |
| STATE UNIVERSITY OF NEW YORK SUNY STONY BROOK | 13 | 0.516 |
| UNIVERSITY OF GOTHENBURG | 13 | 0.516 |
| UNIVERSITY OF GRONINGEN | 13 | 0.516 |
| UNIVERSITY OF HAMBURG | 13 | 0.516 |
| UNIVERSITY OF NEW SOUTH WALES SYDNEY | 13 | 0.516 |
| UNIVERSITY OF UTAH | 13 | 0.516 |
| UTAH SYSTEM OF HIGHER EDUCATION | 13 | 0.516 |
| UTMD ANDERSON CANCER CENTER | 13 | 0.516 |
| ALBERT EINSTEIN COLLEGE OF MEDICINE | 12 | 0.476 |
| CASE WESTERN RESERVE UNIVERSITY | 12 | 0.476 |
| CLEVELAND CLINIC FOUNDATION | 12 | 0.476 |
| ONDOKUZ MAYIS UNIVERSITY | 12 | 0.476 |
| PEKING UNIVERSITY | 12 | 0.476 |
| RAND CORPORATION | 12 | 0.476 |
| UNIVERSITY OF ALABAMA BIRMINGHAM | 12 | 0.476 |
| UNIVERSITY OF ALBERTA | 12 | 0.476 |
| UNIVERSITY OF BARCELONA | 12 | 0.476 |
| UNIVERSITY OF BERN | 12 | 0.476 |
| UNIVERSITY OF HONG KONG | 12 | 0.476 |
| UNIVERSITY OF IOWA | 12 | 0.476 |
| UNIVERSITY OF KENTUCKY | 12 | 0.476 |
| UNIVERSITY SYSTEM OF MARYLAND | 12 | 0.476 |
| VA CONNECTICUT HEALTHCARE SYSTEM | 12 | 0.476 |
| ARIZONA STATE UNIVERSITY | 11 | 0.437 |
| DALHOUSIE UNIVERSITY | 11 | 0.437 |
| DARTMOUTH COLLEGE | 11 | 0.437 |
| ICAHN SCHOOL OF MEDICINE AT MOUNT SINAI | 11 | 0.437 |
| INSTITUTE OF PSYCHOLOGY CAS | 11 | 0.437 |
| LINKOPING UNIVERSITY | 11 | 0.437 |
| MCLEAN HOSPITAL | 11 | 0.437 |
| NATIONAL TAIWAN UNIVERSITY | 11 | 0.437 |
| PFIZER | 11 | 0.437 |
| ROYAL NORTH SHORE HOSPITAL | 11 | 0.437 |
| RUHR UNIVERSITY BOCHUM | 11 | 0.437 |
| RUTGERS STATE UNIVERSITY MEDICAL CENTER | 11 | 0.437 |
| UNIVERSITY OF EDINBURGH | 11 | 0.437 |
| UNIVERSITY OF HELSINKI | 11 | 0.437 |
| UNIVERSITY OF MONTREAL | 11 | 0.437 |
| GERIATRIC RESEARCH EDUCATION CLINICAL CENTER | 10 | 0.397 |
| HAUKELAND UNIVERSITY HOSPITAL | 10 | 0.397 |
| HENRY FORD HOSPITAL | 10 | 0.397 |
| JOHNS HOPKINS BLOOMBERG SCHOOL OF PUBLIC HEALTH | 10 | 0.397 |
| JOHNS HOPKINS MEDICINE | 10 | 0.397 |
| KAISER PERMANENTE | 10 | 0.397 |
| KEELE UNIVERSITY | 10 | 0.397 |
| NIH NATIONAL INSTITUTE ON DRUG ABUSE NIDA | 10 | 0.397 |
| PHILIPPS UNIVERSITY MARBURG | 10 | 0.397 |
| QIMR BERGHOFER MEDICAL RESEARCH INSTITUTE | 10 | 0.397 |
| SAARLAND UNIVERSITY | 10 | 0.397 |
| STATE UNIVERSITY OF NEW YORK SUNY BUFFALO | 10 | 0.397 |
| UNIVERSITY HEALTH NETWORK TORONTO | 10 | 0.397 |
| UNIVERSITY OF CONNECTICUT | 10 | 0.397 |
| UNIVERSITY OF EASTERN FINLAND | 10 | 0.397 |
| UNIVERSITY OF FREIBURG | 10 | 0.397 |
| UNIVERSITY OF MINNESOTA SYSTEM | 10 | 0.397 |
| UNIVERSITY OF MINNESOTA TWIN CITIES | 10 | 0.397 |
| UNIVERSITY OF MUNICH | 10 | 0.397 |
| UNIVERSITY OF SOUTH FLORIDA | 10 | 0.397 |
| VA PITTSBURGH HEALTHCARE SYSTEM | 10 | 0.397 |
| WESTERN PSYCHIATRIC INSTITUTE CLINIC OF UPMC | 10 | 0.397 |
| COMPLUTENSE UNIVERSITY OF MADRID | 9 | 0.357 |
| CONSIGLIO NAZIONALE DELLE RICERCHE CNR | 9 | 0.357 |
| GROUP HEALTH COOPERATIVE | 9 | 0.357 |
| KAROLINSKA UNIVERSITY HOSPITAL | 9 | 0.357 |
| KUOPIO UNIVERSITY HOSPITAL | 9 | 0.357 |
| MONASH UNIVERSITY | 9 | 0.357 |
| NORTHWESTERN UNIVERSITY | 9 | 0.357 |
| OHIO STATE UNIVERSITY | 9 | 0.357 |
| OTTAWA HOSPITAL RESEARCH INSTITUTE | 9 | 0.357 |
| ROUDEBUSH VA MED CTR | 9 | 0.357 |
| SAINT LOUIS UNIVERSITY | 9 | 0.357 |
| SWEDISH MEDICAL CENTER | 9 | 0.357 |
| TEL AVIV UNIVERSITY | 9 | 0.357 |
| UNIVERSIDADE FEDERAL DE SAO PAULO UNIFESP | 9 | 0.357 |
| UNIVERSITY OF COLORADO SYSTEM | 9 | 0.357 |
| UNIVERSITY OF ILLINOIS SYSTEM | 9 | 0.357 |
| UNIVERSITY OF MARYLAND BALTIMORE | 9 | 0.357 |
| UNIVERSITY OF MELBOURNE | 9 | 0.357 |
| UNIVERSITY OF PISA | 9 | 0.357 |
| UNIVERSITY OF TEXAS DALLAS | 9 | 0.357 |
| UNIVERSITY OF VIRGINIA | 9 | 0.357 |
| UNIVERSITY OF ZURICH | 9 | 0.357 |
| UTRECHT UNIVERSITY | 9 | 0.357 |
| WESTERN UNIVERSITY UNIVERSITY OF WESTERN ONTARIO | 9 | 0.357 |
| BOSTON UNIVERSITY | 8 | 0.318 |
| CHINESE UNIVERSITY OF HONG KONG | 8 | 0.318 |
| FINNISH INSTITUTE OF OCCUPATIONAL HEALTH | 8 | 0.318 |
| FOURTH MILITARY MEDICAL UNIVERSITY | 8 | 0.318 |
| GEORGE INSTITUTE FOR GLOBAL HEALTH | 8 | 0.318 |
| GLAXOSMITHKLINE | 8 | 0.318 |
| HELSINKI UNIVERSITY CENTRAL HOSPITAL | 8 | 0.318 |
| MCMASTER UNIVERSITY | 8 | 0.318 |
| MEDICAL UNIVERSITY OF SOUTH CAROLINA | 8 | 0.318 |
| MEMORIAL SLOAN KETTERING CANCER CENTER | 8 | 0.318 |
| NEW YORK STATE PSYCHIATRY INSTITUTE | 8 | 0.318 |
| NIH NATIONAL INSTITUTE ON ALCOHOL ABUSE ALCOHOLISM NIAAA | 8 | 0.318 |
| QUEEN MARY UNIVERSITY LONDON | 8 | 0.318 |
| RESEARCH TRIANGLE INSTITUTE | 8 | 0.318 |
| SACKLER FACULTY OF MEDICINE | 8 | 0.318 |
| SAN DIEGO STATE UNIVERSITY | 8 | 0.318 |
| TECHNICAL UNIVERSITY OF MUNICH | 8 | 0.318 |
| TILBURG UNIVERSITY | 8 | 0.318 |
| TUFTS UNIVERSITY | 8 | 0.318 |
| UNIVERSIDAD DE CADIZ | 8 | 0.318 |
| UNIVERSITY OF ADELAIDE | 8 | 0.318 |
| UNIVERSITY OF ARIZONA | 8 | 0.318 |
| UNIVERSITY OF ARKANSAS SYSTEM | 8 | 0.318 |
| UNIVERSITY OF BASEL | 8 | 0.318 |
| UNIVERSITY OF BRITISH COLUMBIA | 8 | 0.318 |
| UNIVERSITY OF MANITOBA | 8 | 0.318 |
| UNIVERSITY OF OULU | 8 | 0.318 |
| UNIVERSITY OF OXFORD | 8 | 0.318 |
| UNIVERSITY OF SOUTHERN CALIFORNIA | 8 | 0.318 |
| UNIVERSITY OF WISCONSIN MADISON | 8 | 0.318 |
| UNIVERSITY OF WISCONSIN SYSTEM | 8 | 0.318 |
| VA SAN DIEGO HEALTHCARE SYSTEM | 8 | 0.318 |
| WAYNE STATE UNIVERSITY | 8 | 0.318 |
| AUTONOMOUS UNIVERSITY OF BARCELONA | 7 | 0.278 |
| BEN GURION UNIVERSITY | 7 | 0.278 |
| BOEHRINGER INGELHEIM | 7 | 0.278 |
| DREXEL UNIVERSITY | 7 | 0.278 |
| FEINBERG SCHOOL OF MEDICINE | 7 | 0.278 |
| FUDAN UNIVERSITY | 7 | 0.278 |
| GGZ INGEEST | 7 | 0.278 |
| HACETTEPE UNIVERSITY | 7 | 0.278 |
| INSTITUT NATIONAL DE LA SANTE ET DE LA RECHERCHE MEDICALE INSERM | 7 | 0.278 |
| KAOHSIUNG MEDICAL UNIVERSITY | 7 | 0.278 |
| KOREA UNIVERSITY | 7 | 0.278 |
| NATIONAL TAIWAN UNIVERSITY HOSPITAL | 7 | 0.278 |
| NATIONAL UNIVERSITY OF IRELAND NUI GALWAY | 7 | 0.278 |
| NIH NATIONAL INSTITUTE OF MENTAL HEALTH NIMH | 7 | 0.278 |
| NIH NATIONAL INSTITUTE ON AGING NIA | 7 | 0.278 |
| RIGSHOSPITALET | 7 | 0.278 |
| SEMMELWEIS UNIVERSITY | 7 | 0.278 |
| SHAHED UNIVERSITY | 7 | 0.278 |
| SORBONNE UNIVERSITE | 7 | 0.278 |
| UNIVERSIDADE DE LISBOA | 7 | 0.278 |
| UNIVERSITY HOSPITAL LEUVEN | 7 | 0.278 |
| UNIVERSITY HOSPITAL OF BERN | 7 | 0.278 |
| UNIVERSITY OF ALABAMA TUSCALOOSA | 7 | 0.278 |
| UNIVERSITY OF ARKANSAS MEDICAL SCIENCES | 7 | 0.278 |
| UNIVERSITY OF BIRMINGHAM | 7 | 0.278 |
| UNIVERSITY OF DUISBURG ESSEN | 7 | 0.278 |
| UNIVERSITY OF ILLINOIS CHICAGO | 7 | 0.278 |
| UNIVERSITY OF ILLINOIS CHICAGO HOSPITAL | 7 | 0.278 |
| UNIVERSITY OF KANSAS | 7 | 0.278 |
| UNIVERSITY OF LEIPZIG | 7 | 0.278 |
| UNIVERSITY OF LIVERPOOL | 7 | 0.278 |
| UNIVERSITY OF MISSISSIPPI | 7 | 0.278 |
| UNIVERSITY OF NAPLES FEDERICO II | 7 | 0.278 |
| UNIVERSITY OF NEW MEXICO | 7 | 0.278 |
| UNIVERSITY OF PAVIA | 7 | 0.278 |
| UNIVERSITY OF QUEENSLAND | 7 | 0.278 |
| UNIVERSITY OF TEXAS HEALTH SAN ANTONIO | 7 | 0.278 |
| UNIVERSITY OF VERMONT | 7 | 0.278 |
| WAKE FOREST UNIVERSITY | 7 | 0.278 |
| YORK UNIVERSITY CANADA | 7 | 0.278 |
| CEDARS SINAI MEDICAL CENTER | 6 | 0.238 |
| CHANG GUNG UNIVERSITY OF SCIENCE TECHNOLOGY | 6 | 0.238 |
| DICLE UNIVERSITY | 6 | 0.238 |
| ERASMUS UNIVERSITY ROTTERDAM | 6 | 0.238 |
| FLINDERS UNIVERSITY SOUTH AUSTRALIA | 6 | 0.238 |
| HALLYM UNIVERSITY | 6 | 0.238 |
| HARBORVIEW MEDICAL CENTER | 6 | 0.238 |
| HOSPITAL CLINICO UNIVERSITARIO VIRGEN DE LA ARRIXACA | 6 | 0.238 |
| HUMBOLDT UNIVERSITY OF BERLIN | 6 | 0.238 |
| LOYOLA UNIVERSITY CHICAGO | 6 | 0.238 |
| MONTEFIORE MEDICAL CENTER | 6 | 0.238 |
| NATIONAL UNIVERSITY OF SINGAPORE | 6 | 0.238 |
| NEWCASTLE UNIVERSITY UK | 6 | 0.238 |
| PHILADELPHIA VETERANS AFFAIRS MEDICAL CENTER | 6 | 0.238 |
| RICHARD L ROUDEBUSH VA MEDICAL CENTER | 6 | 0.238 |
| RWTH AACHEN UNIVERSITY | 6 | 0.238 |
| SAHLGRENSKA UNIVERSITY HOSPITAL | 6 | 0.238 |
| SEOUL NATIONAL UNIVERSITY HOSPITAL | 6 | 0.238 |
| SHANDONG UNIVERSITY | 6 | 0.238 |
| SHIONOGI CO LTD | 6 | 0.238 |
| STOCKHOLM UNIVERSITY | 6 | 0.238 |
| TAMPERE UNIVERSITY | 6 | 0.238 |
| TAMPERE UNIVERSITY HOSPITAL | 6 | 0.238 |
| TRINITY COLLEGE DUBLIN | 6 | 0.238 |
| UNIVERSIDAD DE ALMERIA | 6 | 0.238 |
| UNIVERSITY OF CALIFORNIA DAVIS | 6 | 0.238 |
| UNIVERSITY OF CHICAGO | 6 | 0.238 |
| UNIVERSITY OF GENEVA | 6 | 0.238 |
| UNIVERSITY OF GEORGIA | 6 | 0.238 |
| UNIVERSITY OF KANSAS MEDICAL CENTER | 6 | 0.238 |
| UNIVERSITY OF MILAN | 6 | 0.238 |
| UNIVERSITY OF MISSOURI SYSTEM | 6 | 0.238 |
| UNIVERSITY OF MURCIA | 6 | 0.238 |
| UNIVERSITY OF SHERBROOKE | 6 | 0.238 |
| UNIVERSITY OF SOUTHERN DENMARK | 6 | 0.238 |
| AARHUS UNIVERSITY | 5 | 0.198 |
| ASTRAZENECA | 5 | 0.198 |
| BOSTON CHILDREN S HOSPITAL | 5 | 0.198 |
| BUTLER HOSP | 5 | 0.198 |
| CARDIFF UNIVERSITY | 5 | 0.198 |
| CHAIM SHEBA MEDICAL CENTER | 5 | 0.198 |
| CHILDRENS HOSPITAL OF PHILADELPHIA | 5 | 0.198 |
| CHINESE PEOPLE S LIBERATION ARMY GENERAL HOSPITAL | 5 | 0.198 |
| CHUNG ANG UNIVERSITY | 5 | 0.198 |
| EBERHARD KARLS UNIVERSITY OF TUBINGEN | 5 | 0.198 |
| GEORGE WASHINGTON UNIVERSITY | 5 | 0.198 |
| GEORGETOWN UNIVERSITY | 5 | 0.198 |
| GUY S ST THOMAS NHS FOUNDATION TRUST | 5 | 0.198 |
| HEBREW SENIORLIFE | 5 | 0.198 |
| HOSPITAL ISRAELITA ALBERT EINSTEIN | 5 | 0.198 |
| INSTITUTO DE SALUD CARLOS III | 5 | 0.198 |
| IRCCS FONDAZIONE CASIMIRO MONDINO | 5 | 0.198 |
| ISTANBUL UNIVERSITY | 5 | 0.198 |
| KEIO UNIVERSITY | 5 | 0.198 |
| KYOTO UNIVERSITY | 5 | 0.198 |
| LA TROBE UNIVERSITY | 5 | 0.198 |
| MEDICAL UNIVERSITY OF VIENNA | 5 | 0.198 |
| MURDOCH UNIVERSITY | 5 | 0.198 |
| NATIONAL CHENG KUNG UNIVERSITY | 5 | 0.198 |
| NATIONAL DATA BANK FOR RHEUMATIC DISEASES | 5 | 0.198 |
| OHIO UNIVERSITY | 5 | 0.198 |
| PAMUKKALE UNIVERSITY | 5 | 0.198 |
| QUEENS UNIVERSITY CANADA | 5 | 0.198 |
| QUEENSLAND UNIVERSITY OF TECHNOLOGY QUT | 5 | 0.198 |
| ROYAL HOLLOWAY UNIVERSITY LONDON | 5 | 0.198 |
| SICHUAN UNIVERSITY | 5 | 0.198 |
| SOROKA MEDICAL CENTER | 5 | 0.198 |
| SUN YAT SEN UNIVERSITY | 5 | 0.198 |
| SYRACUSE UNIVERSITY | 5 | 0.198 |
| TAIPEI MEDICAL UNIVERSITY | 5 | 0.198 |
| TRI SERVICE GENERAL HOSPITAL | 5 | 0.198 |
| TRIMBOS INSTITUTE | 5 | 0.198 |
| UMEA UNIVERSITY | 5 | 0.198 |
| UNIVERSITY OF AMSTERDAM | 5 | 0.198 |
| UNIVERSITY OF CHINESE ACADEMY OF SCIENCES CAS | 5 | 0.198 |
| UNIVERSITY OF COLORADO BOULDER | 5 | 0.198 |
| UNIVERSITY OF COLORADO HEALTH SCIENCE CENTER | 5 | 0.198 |
| UNIVERSITY OF GENOA | 5 | 0.198 |
| UNIVERSITY OF GLASGOW | 5 | 0.198 |
| UNIVERSITY OF GOTTINGEN | 5 | 0.198 |
| UNIVERSITY OF HOUSTON | 5 | 0.198 |
| UNIVERSITY OF HOUSTON SYSTEM | 5 | 0.198 |
| UNIVERSITY OF LEEDS | 5 | 0.198 |
| UNIVERSITY OF LOUISVILLE | 5 | 0.198 |
| UNIVERSITY OF MASSACHUSETTS SYSTEM | 5 | 0.198 |
| UNIVERSITY OF SALAMANCA | 5 | 0.198 |
| UNIVERSITY OF SOUTHAMPTON | 5 | 0.198 |
| UNIVERSITY OF TOKYO | 5 | 0.198 |
| UNIVERSITY OF TOLEDO | 5 | 0.198 |
| UNIVERSITY OF VALENCIA | 5 | 0.198 |
| UNIVERSITY OF ZAGREB | 5 | 0.198 |
| UPPSALA UNIVERSITY HOSPITAL | 5 | 0.198 |
| VU UNIVERSITY MEDICAL CENTER | 5 | 0.198 |
| WASHINGTON STATE UNIVERSITY | 5 | 0.198 |
| YONSEI UNIVERSITY | 5 | 0.198 |
| YONSEI UNIVERSITY HEALTH SYSTEM | 5 | 0.198 |
| ABANT IZZET BAYSAL UNIVERSITY | 4 | 0.159 |
| ACIBADEM UNIVERSITY | 4 | 0.159 |
| ALLIANT INTERNATIONAL UNIVERSITY | 4 | 0.159 |
| ANKARA PHYSICAL THERAPY REHABILITATION HOSPITAL | 4 | 0.159 |
| ASAN MEDICAL CENTER | 4 | 0.159 |
| BOSTON MEDICAL CENTER | 4 | 0.159 |
| BUDDHIST TZU CHI GENERAL HOSPITAL | 4 | 0.159 |
| CALIF SCH PROFESS PSYCHOL | 4 | 0.159 |
| CALIFORNIA STATE UNIVERSITY LONG BEACH | 4 | 0.159 |
| CATHOLIC UNIVERSITY OF THE SACRED HEART | 4 | 0.159 |
| CENTRAL FINLAND CENTRAL HOSPITAL | 4 | 0.159 |
| CENTRAL INSTITUTE OF MENTAL HEALTH | 4 | 0.159 |
| CENTRAL SOUTH UNIVERSITY | 4 | 0.159 |
| CHARITE MEDICAL UNIVERSITY OF BERLIN | 4 | 0.159 |
| CHULALONGKORN UNIVERSITY | 4 | 0.159 |
| CINCINNATI CHILDREN S HOSPITAL MEDICAL CENTER | 4 | 0.159 |
| CITY UNIVERSITY OF NEW YORK CUNY SYSTEM | 4 | 0.159 |
| DANA FARBER CANCER INSTITUTE | 4 | 0.159 |
| DURHAM VA MEDICAL CENTER | 4 | 0.159 |
| FLORIDA STATE UNIVERSITY | 4 | 0.159 |
| FONDATION I SITE ULNE | 4 | 0.159 |
| FREE UNIVERSITY OF BERLIN | 4 | 0.159 |
| GEORGIA STATE UNIVERSITY | 4 | 0.159 |
| GOETHE UNIVERSITY FRANKFURT | 4 | 0.159 |
| HEINRICH HEINE UNIVERSITY DUSSELDORF | 4 | 0.159 |
| INSTITUT HOSPITAL DEL MAR D INVESTIGACIONS MEDIQUES IMIM | 4 | 0.159 |
| INSTITUTE FOR WORK HEALTH | 4 | 0.159 |
| IRCCS ISTITUTO NEUROLOGICO BESTA | 4 | 0.159 |
| IRCCS POLICLINICO GEMELLI | 4 | 0.159 |
| ISTITUTO DI NEUROSCIENZE IN CNR | 4 | 0.159 |
| JAMES CANCER HOSPITAL SOLOVE RESEARCH INSTITUTE | 4 | 0.159 |
| KANTAR HLTH | 4 | 0.159 |
| KING S COLLEGE HOSPITAL NHS FOUNDATION TRUST | 4 | 0.159 |
| KOLLING INSTITUTE OF MEDICAL RESEARCH | 4 | 0.159 |
| KOREA UNIVERSITY MEDICAL CENTER KUMC | 4 | 0.159 |
| LUND UNIVERSITY | 4 | 0.159 |
| MACQUARIE UNIVERSITY | 4 | 0.159 |
| MERCK COMPANY | 4 | 0.159 |
| MUNICIPAL BERGEN | 4 | 0.159 |
| NANJING MEDICAL UNIVERSITY | 4 | 0.159 |
| NATIONAL DEFENSE MEDICAL CENTER | 4 | 0.159 |
| NORTHWELL HEALTH | 4 | 0.159 |
| NORWEGIAN INSTITUTE OF PUBLIC HEALTH NIPH | 4 | 0.159 |
| OREBRO UNIVERSITY | 4 | 0.159 |
| PENN STATE UNIVERSITY | 4 | 0.159 |
| PRINCE OF WALES HOSPITAL | 4 | 0.159 |
| RADBOUD UNIVERSITY NIJMEGEN | 4 | 0.159 |
| RENMIN UNIVERSITY OF CHINA | 4 | 0.159 |
| ROUDEBUSH VAMC | 4 | 0.159 |
| SEATTLE CHILDREN S HOSPITAL | 4 | 0.159 |
| TEL AVIV SOURASKY MEDICAL CENTER | 4 | 0.159 |
| TEMPLE UNIVERSITY | 4 | 0.159 |
| TEXAS A M HEALTH SCIENCE CENTER | 4 | 0.159 |
| TEXAS A M UNIVERSITY SYSTEM | 4 | 0.159 |
| UCL MEDICAL SCHOOL | 4 | 0.159 |
| ULM UNIVERSITY | 4 | 0.159 |
| UNITED STATES DEPARTMENT OF DEFENSE | 4 | 0.159 |
| UNIVERSIDADE DE COIMBRA | 4 | 0.159 |
| UNIVERSIDADE FEDERAL DO PARANA | 4 | 0.159 |
| UNIVERSITE LILLE NORD DE FRANCE COMUE | 4 | 0.159 |
| UNIVERSITE PARIS SACLAY | 4 | 0.159 |
| UNIVERSITE PARIS SACLAY COMUE | 4 | 0.159 |
| UNIVERSITY COLLEGE LONDON HOSPITALS NHS FOUNDATION TRUST | 4 | 0.159 |
| UNIVERSITY OF ABERDEEN | 4 | 0.159 |
| UNIVERSITY OF ANTWERP | 4 | 0.159 |
| UNIVERSITY OF ATHENS | 4 | 0.159 |
| UNIVERSITY OF AUCKLAND | 4 | 0.159 |
| UNIVERSITY OF BATH | 4 | 0.159 |
| UNIVERSITY OF CALIFORNIA IRVINE | 4 | 0.159 |
| UNIVERSITY OF CAMBRIDGE | 4 | 0.159 |
| UNIVERSITY OF FLORENCE | 4 | 0.159 |
| UNIVERSITY OF KENT | 4 | 0.159 |
| UNIVERSITY OF LAUSANNE | 4 | 0.159 |
| UNIVERSITY OF MISSOURI COLUMBIA | 4 | 0.159 |
| UNIVERSITY OF MUNSTER | 4 | 0.159 |
| UNIVERSITY OF NEBRASKA SYSTEM | 4 | 0.159 |
| UNIVERSITY OF NEW ENGLAND USA | 4 | 0.159 |
| UNIVERSITY OF NORTH TEXAS SYSTEM | 4 | 0.159 |
| UNIVERSITY OF OTAGO | 4 | 0.159 |
| UNIVERSITY OF RIJEKA | 4 | 0.159 |
| UNIVERSITY OF ROME TOR VERGATA | 4 | 0.159 |
| UNIVERSITY OF SIENA | 4 | 0.159 |
| UNIVERSITY OF SOUTH CAROLINA COLUMBIA | 4 | 0.159 |
| UNIVERSITY OF SOUTH CAROLINA SYSTEM | 4 | 0.159 |
| UNIVERSITY OF TEXAS ARLINGTON | 4 | 0.159 |
| UNIVERSITY OF TEXAS AUSTIN | 4 | 0.159 |
| UNIVERSITY OF TEXAS SCHOOL PUBLIC HEALTH | 4 | 0.159 |
| UNIVERSITY OF TOLEDO HEALTH SCIENCE CAMPUS | 4 | 0.159 |
| UNIVERSITY OF TURKU | 4 | 0.159 |
| UNIVERSITY OF ULSAN | 4 | 0.159 |
| UNIVERSITY OF VIENNA | 4 | 0.159 |
| UNIVERSITY OF WESTERN AUSTRALIA | 4 | 0.159 |
| WYETH AYERST | 4 | 0.159 |
| ZHEJIANG UNIVERSITY | 4 | 0.159 |
| AFYON KOCATEPE UNIVERSITY | 3 | 0.119 |
| ANKARA NUMUNE TRAINING RESEARCH HOSPITAL | 3 | 0.119 |
| ARKIN | 3 | 0.119 |
| ATHENS MEDICAL SCHOOL | 3 | 0.119 |
| AUGUSTA UNIVERSITY | 3 | 0.119 |
| AUTONOMOUS UNIVERSITY OF MADRID | 3 | 0.119 |
| BLACK DOG INSTITUTE | 3 | 0.119 |
| BULGARIAN ACADEMY OF SCIENCES | 3 | 0.119 |
| CASE WESTERN RESERVE UNIVERSITY HOSPITAL | 3 | 0.119 |
| CATALAN HEALTH INSTITUTE | 3 | 0.119 |
| CENTRE HOSPITALIER UNIVERSITAIRE VAUDOIS CHUV | 3 | 0.119 |
| CENTRO HOSPITALAR DE LISBOA OCIDENTAL EPE | 3 | 0.119 |
| CHANGHUA CHRISTIAN HOSPITAL | 3 | 0.119 |
| CHARLES UNIVERSITY PRAGUE | 3 | 0.119 |
| CHENGDU UNIVERSITY OF TRADITIONAL CHINESE MEDICINE | 3 | 0.119 |
| CHILD FAMILY RESEARCH INSTITUTE | 3 | 0.119 |
| CHU NICE | 3 | 0.119 |
| CHUNG HO MEMORIAL HOSPITAL | 3 | 0.119 |
| COMMUNITY HLTH NETWORK | 3 | 0.119 |
| CUMHURIYET UNIVERSITY | 3 | 0.119 |
| DEPT PSYCHIAT | 3 | 0.119 |
| EGAS MONIZ HOSPITAL | 3 | 0.119 |
| ERCIYES UNIVERSITY | 3 | 0.119 |
| ERNST MORITZ ARNDT UNIVERSITAT GREIFSWALD | 3 | 0.119 |
| ETH ZURICH | 3 | 0.119 |
| EUROPEAN UNIVERSITY OF MADRID | 3 | 0.119 |
| EWHA WOMANS UNIVERSITY | 3 | 0.119 |
| FAR EASTERN MEMORIAL HOSPITAL | 3 | 0.119 |
| FLINDERS MEDICAL CENTRE | 3 | 0.119 |
| FLOREY INSTITUTE OF NEUROSCIENCE MENTAL HEALTH | 3 | 0.119 |
| FORDHAM UNIVERSITY | 3 | 0.119 |
| GAZI UNIVERSITY | 3 | 0.119 |
| GAZIANTEP UNIVERSITY | 3 | 0.119 |
| GGZ BREBURG | 3 | 0.119 |
| GGZINGEEST | 3 | 0.119 |
| GREEN LANE HOSP | 3 | 0.119 |
| GRIFFITH UNIVERSITY | 3 | 0.119 |
| GULHANE MILITARY MEDICAL ACADEMY | 3 | 0.119 |
| HARRAN UNIVERSITY | 3 | 0.119 |
| HEBREW UNIVERSITY OF JERUSALEM | 3 | 0.119 |
| HITSUJI CLIN | 3 | 0.119 |
| HOFSTRA UNIVERSITY | 3 | 0.119 |
| HONG KONG POLYTECHNIC UNIVERSITY | 3 | 0.119 |
| HOSPITAL CLINIC DE BARCELONA | 3 | 0.119 |
| HOSPITAL DEL MAR | 3 | 0.119 |
| HOSPITAL UNIVERSITARIO 12 DE OCTUBRE | 3 | 0.119 |
| HOSPITAL UNIVERSITY HOSPITAL G MARTINO | 3 | 0.119 |
| HOTCHKISS BRAIN INST | 3 | 0.119 |
| HUNGARIAN ACADEMY OF SCIENCES | 3 | 0.119 |
| INDIANA UNIVERSITY BLOOMINGTON | 3 | 0.119 |
| INONU UNIVERSITY | 3 | 0.119 |
| IRCCS SANTA LUCIA | 3 | 0.119 |
| JAMES COOK UNIVERSITY | 3 | 0.119 |
| JILIN UNIVERSITY | 3 | 0.119 |
| JINNAH POSTGRAD MED CTR | 3 | 0.119 |
| JORDAN UNIVERSITY OF SCIENCE TECHNOLOGY | 3 | 0.119 |
| KAHRAMANMARAS SUTCU IMAM UNIVERSITY | 3 | 0.119 |
| KAOHSIUNG MUNICIPAL KAI SYUAN PSYCHIAT HOSP | 3 | 0.119 |
| KEIMYUNG UNIVERSITY | 3 | 0.119 |
| KING S COLLEGE HOSPITAL | 3 | 0.119 |
| KOCAELI UNIVERSITY | 3 | 0.119 |
| KYORIN UNIVERSITY | 3 | 0.119 |
| LANGUEDOC ROUSSILLON UNIVERSITES COMUE | 3 | 0.119 |
| LIVERPOOL JOHN MOORES UNIVERSITY | 3 | 0.119 |
| LUIGI SACCO HOSPITAL | 3 | 0.119 |
| MARMARA UNIVERSITY | 3 | 0.119 |
| MARTIN LUTHER UNIVERSITY HALLE WITTENBERG | 3 | 0.119 |
| MEDICAL UNIVERSITY VARNA | 3 | 0.119 |
| MINISTRY OF HEALTH TURKEY | 3 | 0.119 |
| MINNEAPOLIS VA HEALTH CARE SYSTEM | 3 | 0.119 |
| MONTEFIORE HEADACHE CTR | 3 | 0.119 |
| NASHVILLE NEUROSCI GRP | 3 | 0.119 |
| NATIONAL RESEARCH CENTRE FOR THE WORKING ENVIRONMENT | 3 | 0.119 |
| NEWYORK PRESBYTERIAN HOSPITAL | 3 | 0.119 |
| NIH CLINICAL CENTER CC | 3 | 0.119 |
| NIH NATIONAL INSTITUTE OF DENTAL CRANIOFACIAL RESEARCH NIDCR | 3 | 0.119 |
| NIPPON MEDICAL SCHOOL | 3 | 0.119 |
| OSAKA UNIVERSITY | 3 | 0.119 |
| PANJAB UNIVERSITY | 3 | 0.119 |
| PORTLAND VA MEDICAL CENTER | 3 | 0.119 |
| POZNAN UNIVERSITY OF MEDICAL SCIENCES | 3 | 0.119 |
| PRN CONSULTING | 3 | 0.119 |
| PUSAN NATIONAL UNIVERSITY | 3 | 0.119 |
| ROYAL MARSDEN NHS FOUNDATION TRUST | 3 | 0.119 |
| ROYAL MELBOURNE HOSPITAL | 3 | 0.119 |
| RUSSIAN ACADEMY OF MEDICAL SCIENCE | 3 | 0.119 |
| RUSSIAN ACADEMY OF SCIENCES | 3 | 0.119 |
| SAGA UNIVERSITY | 3 | 0.119 |
| SAKARYA UNIVERSITY | 3 | 0.119 |
| SAN DIEGO VET AFFAIRS MED CTR | 3 | 0.119 |
| SCRIPPS RESEARCH INSTITUTE | 3 | 0.119 |
| SECOND MILITARY MEDICAL UNIVERSITY | 3 | 0.119 |
| SHANGHAI JIAO TONG UNIVERSITY | 3 | 0.119 |
| SHIN KONG WU HO SU MEMORIAL HOSPITAL | 3 | 0.119 |
| SHIOIRI MENTAL CLIN | 3 | 0.119 |
| SOONCHUNHYANG UNIVERSITY | 3 | 0.119 |
| SOUTH LONDON MAUDSLEY NHS TRUST | 3 | 0.119 |
| ST GEORGES UNIVERSITY LONDON | 3 | 0.119 |
| STATE UNIVERSITY OF NEW YORK SUNY UPSTATE MEDICAL CENTER | 3 | 0.119 |
| STAVANGER UNIVERSITY HOSPITAL | 3 | 0.119 |
| SULEYMAN DEMIREL UNIVERSITY | 3 | 0.119 |
| TEHRAN UNIVERSITY OF MEDICAL SCIENCES | 3 | 0.119 |
| THE ROYAL MARSDEN HOSPITAL LONDON | 3 | 0.119 |
| TONGJI UNIVERSITY | 3 | 0.119 |
| ULUDAG UNIVERSITY | 3 | 0.119 |
| UNI RES | 3 | 0.119 |
| UNITED CHRISTIAN HOSPITAL | 3 | 0.119 |
| UNITED STATES NAVY | 3 | 0.119 |
| UNIV APPL SCI ARTS SOUTHERN SWITZERLAND | 3 | 0.119 |
| UNIV TRONDHEIM | 3 | 0.119 |
| UNIVERSIDAD DE ALCALA | 3 | 0.119 |
| UNIVERSIDADE FEDERAL DA BAHIA | 3 | 0.119 |
| UNIVERSIDADE FEDERAL DE MINAS GERAIS | 3 | 0.119 |
| UNIVERSIDADE FEDERAL DE SANTA MARIA UFSM | 3 | 0.119 |
| UNIVERSIDADE NOVA DE LISBOA | 3 | 0.119 |
| UNIVERSITAT DE LES ILLES BALEARS | 3 | 0.119 |
| UNIVERSITE DE LILLE | 3 | 0.119 |
| UNIVERSITE DE MONTPELLIER | 3 | 0.119 |
| UNIVERSITE PARIS SUD PARIS 11 | 3 | 0.119 |
| UNIVERSITY HOSPITALS OF CLEVELAND | 3 | 0.119 |
| UNIVERSITY OF BASQUE COUNTRY | 3 | 0.119 |
| UNIVERSITY OF BOLOGNA | 3 | 0.119 |
| UNIVERSITY OF BONN | 3 | 0.119 |
| UNIVERSITY OF BRISTOL | 3 | 0.119 |
| UNIVERSITY OF CATANIA | 3 | 0.119 |
| UNIVERSITY OF DUNDEE | 3 | 0.119 |
| UNIVERSITY OF ERLANGEN NUREMBERG | 3 | 0.119 |
| UNIVERSITY OF GREENWICH | 3 | 0.119 |
| UNIVERSITY OF HAWAII SYSTEM | 3 | 0.119 |
| UNIVERSITY OF LEICESTER | 3 | 0.119 |
| UNIVERSITY OF LIEGE | 3 | 0.119 |
| UNIVERSITY OF LJUBLJANA | 3 | 0.119 |
| UNIVERSITY OF MESSINA | 3 | 0.119 |
| UNIVERSITY OF MISSISSIPPI MEDICAL CENTER | 3 | 0.119 |
| UNIVERSITY OF NORTH TEXAS DENTON | 3 | 0.119 |
| UNIVERSITY OF PARMA | 3 | 0.119 |
| UNIVERSITY OF PUERTO RICO | 3 | 0.119 |
| UNIVERSITY OF QUEBEC | 3 | 0.119 |
| UNIVERSITY OF REGENSBURG | 3 | 0.119 |
| UNIVERSITY OF SASKATCHEWAN | 3 | 0.119 |
| UNIVERSITY OF SHEFFIELD | 3 | 0.119 |
| UNIVERSITY OF SOUTH AUSTRALIA | 3 | 0.119 |
| UNIVERSITY OF SOUTH CAROLINA | 3 | 0.119 |
| UNIVERSITY OF SOUTH DAKOTA | 3 | 0.119 |
| UTRECHT UNIVERSITY MEDICAL CENTER | 3 | 0.119 |
| VA ANN ARBOR HEALTHCARE SYSTEM | 3 | 0.119 |
| VA GREATER LOS ANGELES HEALTHCARE SYSTEM | 3 | 0.119 |
| VA LONG BEACH HEALTHCARE SYSTEM | 3 | 0.119 |
| VA STROKE QUERI CTR | 3 | 0.119 |
| WALTON CENTRE | 3 | 0.119 |
| WEILL CORNELL MEDICAL COLLEGE QATAR | 3 | 0.119 |
| WORLD HEALTH ORGANIZATION | 3 | 0.119 |
| XI AN JIAOTONG UNIVERSITY | 3 | 0.119 |
| ZHEJIANG CHINESE MEDICAL UNIVERSITY | 3 | 0.119 |
| ZNA STUIVENBERG | 3 | 0.119 |
| ABARBANEL MENTAL HLTH CTR | 2 | 0.079 |
| ACADEMIC MEDICAL CENTER AMSTERDAM | 2 | 0.079 |
| ACADEMY OF MILITARY MEDICAL SCIENCES CHINA | 2 | 0.079 |
| ACIBADEM HASTANELERI | 2 | 0.079 |
| ACIBADEM HOSPITALS GROUP | 2 | 0.079 |
| ADDIS ABABA UNIVERSITY | 2 | 0.079 |
| AKDENIZ UNIVERSITY | 2 | 0.079 |
| ALBANY MEDICAL COLLEGE | 2 | 0.079 |
| ALBERTA CHILDRENS HOSPITAL | 2 | 0.079 |
| ALCORCON FOUNDATION UNIVERSITY HOSPITAL | 2 | 0.079 |
| ALICE HO MIU LING NETHERSOLE HOSPITAL | 2 | 0.079 |
| AN DING HOSP | 2 | 0.079 |
| ANGLIA RUSKIN UNIVERSITY | 2 | 0.079 |
| ARIEL UNIVERSITY | 2 | 0.079 |
| ARISTOTLE UNIVERSITY OF THESSALONIKI | 2 | 0.079 |
| ARZTPRAXIS HOTTINGEN | 2 | 0.079 |
| ASANTE PHYS PARTNERS | 2 | 0.079 |
| ATATURK UNIVERSITY | 2 | 0.079 |
| ATHENS NAVAL HOSP | 2 | 0.079 |
| AUCKLAND UNIVERSITY OF TECHNOLOGY | 2 | 0.079 |
| AVRASYA HOSPITAL | 2 | 0.079 |
| AVRUPA SAFAK HOSPITAL | 2 | 0.079 |
| BALIKESIR UNIVERSITY | 2 | 0.079 |
| BANARAS HINDU UNIVERSITY | 2 | 0.079 |
| BAYLOR HEALTH CARE SYSTEM | 2 | 0.079 |
| BC WOMEN S HOSPITAL HEALTH CENTRE | 2 | 0.079 |
| BOSTON COLLEGE | 2 | 0.079 |
| BOZOK UNIVERSITY | 2 | 0.079 |
| BRISTOL MYERS SQUIBB | 2 | 0.079 |
| BRISTOL ROYAL INFIRMARY | 2 | 0.079 |
| BRYCE HOSP | 2 | 0.079 |
| BULENT ECEVIT UNIVERSITY | 2 | 0.079 |
| BUTABIKA NATL REFERRAL MENTAL HLTH HOSP | 2 | 0.079 |
| CALIFORNIA STATE UNIVERSITY FULLERTON | 2 | 0.079 |
| CALIFORNIA STATE UNIVERSITY NORTHRIDGE | 2 | 0.079 |
| CAPITAL MEDICAL UNIVERSITY | 2 | 0.079 |
| CAULFIELD PAIN MANAGEMENT RES CTR | 2 | 0.079 |
| CENT TEXAS VET HLTH CARE SYST | 2 | 0.079 |
| CENTRAL TAIWAN UNIVERSITY SCIENCE TECHNOLOGY | 2 | 0.079 |
| CENTRASTATE MED CTR | 2 | 0.079 |
| CENTRE FOR ADDICTION MENTAL HEALTH CANADA | 2 | 0.079 |
| CENTRE NATIONAL DE LA RECHERCHE SCIENTIFIQUE CNRS | 2 | 0.079 |
| CHANG GUNG UNIV SCI TECHNOL | 2 | 0.079 |
| CHILDRENS HEALTHCARE OF ATLANTA CHOA | 2 | 0.079 |
| CHINA ACADEMY OF CHINESE MEDICAL SCIENCES | 2 | 0.079 |
| CHINA MEDICAL UNIVERSITY HOSPITAL TAIWAN | 2 | 0.079 |
| CHINA MEDICAL UNIVERSITY TAIWAN | 2 | 0.079 |
| CHONGQING MEDICAL UNIVERSITY | 2 | 0.079 |
| CHU DE MONTPELLIER | 2 | 0.079 |
| CHU DE NIMES | 2 | 0.079 |
| CHU LILLE | 2 | 0.079 |
| CHUNGNAM NATIONAL UNIVERSITY | 2 | 0.079 |
| CIBERER | 2 | 0.079 |
| CITY COUNCIL OSTERGOTLAND | 2 | 0.079 |
| CITY UNIVERSITY OF HONG KONG | 2 | 0.079 |
| COLORADO STATE UNIVERSITY | 2 | 0.079 |
| COMMUNITY CANC CARE | 2 | 0.079 |
| CONSEJO SUPERIOR DE INVESTIGACIONES CIENTIFICAS CSIC | 2 | 0.079 |
| CONSORCI SANITARI TERRASSA | 2 | 0.079 |
| COPENHAGEN UNIV HOSP BISPEBJERG FREDERIKSBERG | 2 | 0.079 |
| CTR SALUD ERIA | 2 | 0.079 |
| CTR SLEEP CHRONOBIOL | 2 | 0.079 |
| CTY HOSP RYHOV | 2 | 0.079 |
| DANUBE UNIVERSITY KREMS | 2 | 0.079 |
| DEPT CLIN NEUROSCI | 2 | 0.079 |
| DEPT PSYCHOL | 2 | 0.079 |
| DEPT VET AFFAIRS MED CTR | 2 | 0.079 |
| DOKUZ EYLUL UNIVERSITY | 2 | 0.079 |
| DONGGUK UNIVERSITY | 2 | 0.079 |
| DOW UNIVERSITY OF HEALTH SCIENCES | 2 | 0.079 |
| DRESDEN UNIVERSITY OF TECHNOLOGY | 2 | 0.079 |
| DUMLUPINAR UNIVERSITY | 2 | 0.079 |
| DUZCE UNIVERSITY | 2 | 0.079 |
| DWA HEALTHCARE COMMUN GRP | 2 | 0.079 |
| DYNAMED | 2 | 0.079 |
| E DA HOSPITAL | 2 | 0.079 |
| EAST TENNESSEE STATE UNIVERSITY | 2 | 0.079 |
| EASTERN MICHIGAN UNIVERSITY | 2 | 0.079 |
| EASTERN TORONTO MOVEMENT DISORDERS CTR | 2 | 0.079 |
| EDIRNE STATE HOSPITAL | 2 | 0.079 |
| EDUCATION UNIVERSITY OF HONG KONG EDUHK | 2 | 0.079 |
| EMGO INST HLTH CARE RES EMGO | 2 | 0.079 |
| ERASMUS UNIVERSITY MEDICAL CENTER | 2 | 0.079 |
| ESKISEHIR OSMANGAZI UNIVERSITY | 2 | 0.079 |
| FACULDADE DE MEDICINA DE SAO JOSE DO RIO PRETO FAMERP | 2 | 0.079 |
| FINLAND NATIONAL INSTITUTE FOR HEALTH WELFARE | 2 | 0.079 |
| FOOYIN UNIVERSITY | 2 | 0.079 |
| FREDERIKSBORG CENT CTY HOSP | 2 | 0.079 |
| FUJITA HEALTH UNIVERSITY | 2 | 0.079 |
| GAZIOSMANPASA UNIVERSITY | 2 | 0.079 |
| GEISINGER MEDICAL CENTER | 2 | 0.079 |
| GEM CLIN RES CONSULTING M GERBER | 2 | 0.079 |
| GERMANTOWN HOSP MED CTR | 2 | 0.079 |
| GHENT UNIVERSITY | 2 | 0.079 |
| GIRESUN UNIVERSITY | 2 | 0.079 |
| GORDON HOSP | 2 | 0.079 |
| GSS HOSP UNIV SANTA MARIA | 2 | 0.079 |
| HAMAD MEDICAL CORPORATION | 2 | 0.079 |
| HANNOVER MEDICAL SCHOOL | 2 | 0.079 |
| HARRY S TRUMAN MEMORIAL VETERANS HOSPITAL | 2 | 0.079 |
| HASSELT UNIVERSITY | 2 | 0.079 |
| HLTH CTR | 2 | 0.079 |
| HOBART WILLIAM SMITH COLL | 2 | 0.079 |
| HOP ARMAND TROUSSEAU | 2 | 0.079 |
| HOPITAL UNIVERSITAIRE BICETRE APHP | 2 | 0.079 |
| HOPITAL UNIVERSITAIRE LARIBOISIERE FERNAND WIDAL APHP | 2 | 0.079 |
| HOPITAL UNIVERSITAIRE ROTHSCHILD APHP | 2 | 0.079 |
| HOSPITAL CLINICO SAN CARLOS | 2 | 0.079 |
| HOSPITAL FOR SICK CHILDREN SICKKIDS | 2 | 0.079 |
| HOSPITAL PUERTA DE HIERRO MAJADAHONDA | 2 | 0.079 |
| HOSPITAL UNIVERSITARIO SAN CECILIO | 2 | 0.079 |
| HUAZHONG UNIVERSITY OF SCIENCE TECHNOLOGY | 2 | 0.079 |
| HUNT RES CTR | 2 | 0.079 |
| I SHOU UNIVERSITY | 2 | 0.079 |
| INNER MONGOLIA UNIVERSITY OF SCIENCE TECHNOLOGY | 2 | 0.079 |
| INNOVAT MED RES | 2 | 0.079 |
| INOVA FAIRFAX HOSPITAL | 2 | 0.079 |
| INST CLIN RES EDUC MED IREM | 2 | 0.079 |
| INST MENTAL HLTH | 2 | 0.079 |
| INST UNIV SANTE MENTALE MONTREAL | 2 | 0.079 |
| INSTITUTE OF HIGHER NERVOUS ACTIVITY NEUROPHYSIOLOGY OF RAS | 2 | 0.079 |
| INSTITUTO NACIONAL DE PSIQUIATRIA RAMON DE LA FUENTE MUNIZ | 2 | 0.079 |
| INSTITUTO SUPERIOR PSICOLOGIA APLICADA ISPA | 2 | 0.079 |
| INTERNATIONAL UNIVERSITY OF HEALTH WELFARE | 2 | 0.079 |
| INTERUNIVERSITY CARDIOLOGY INSTITUTE OF THE NETHERLANDS | 2 | 0.079 |
| INVENTIV HLTH CLIN LLC | 2 | 0.079 |
| IOWA CITY VA HEALTH CARE SYSTEM | 2 | 0.079 |
| IRCCS CA GRANDA OSPEDALE MAGGIORE POLICLINICO | 2 | 0.079 |
| IRCCS ISTITUTO AUXOLOGICO ITALIANO | 2 | 0.079 |
| IRCCS ISTITUTO NAZIONALE PER LA RICERCA SUL CANCRO IST | 2 | 0.079 |
| IRCCS MARIO NEGRI | 2 | 0.079 |
| ISTANBUL HAYDARPASA NUMUNE TRAINING RESEARCH HOSPITAL | 2 | 0.079 |
| ISTANBUL KARTAL DR LUTFI KIRDAR TRAINING RESEARCH HOSPITAL | 2 | 0.079 |
| ISTITUTO DI FISIOLOGIA CLINICA IFC CNR | 2 | 0.079 |
| IZMIR ATATURK TRAINING RESEARCH HOSPITAL | 2 | 0.079 |
| JICHI MEDICAL UNIVERSITY | 2 | 0.079 |
| JOHANNES GUTENBERG UNIVERSITY OF MAINZ | 2 | 0.079 |
| JUSTUS LIEBIG UNIVERSITY GIESSEN | 2 | 0.079 |
| KAISER PERMANENTE WASHINGTON HLTH RES INST | 2 | 0.079 |
| KARADENIZ TEKNIK UNIVERSITY | 2 | 0.079 |
| KAWASAKI UNIV MED WELF | 2 | 0.079 |
| KINGSTON UNIVERSITY | 2 | 0.079 |
| KLINIKUM SAARBRUCKEN | 2 | 0.079 |
| KLINIKUM SAARBRUCKEN GGMBH | 2 | 0.079 |
| KOCHI UNIVERSITY | 2 | 0.079 |
| KONYA EGITIM TRAINING RESEARCH HOSPITAL | 2 | 0.079 |
| KYAMBOGO UNIV | 2 | 0.079 |
| LAVAL UNIVERSITY | 2 | 0.079 |
| LEV HASHARON MED CTR | 2 | 0.079 |
| LILLY SA | 2 | 0.079 |
| LILLY UK | 2 | 0.079 |
| LITHUANIAN UNIVERSITY OF HEALTH SCIENCES | 2 | 0.079 |
| LOMA LINDA UNIVERSITY | 2 | 0.079 |
| LOUISIANA STATE UNIVERSITY | 2 | 0.079 |
| LOUISIANA STATE UNIVERSITY SYSTEM | 2 | 0.079 |
| LUNDBECK RES | 2 | 0.079 |
| LUNDBECK RES USA | 2 | 0.079 |
| MALMO GEN HOSP | 2 | 0.079 |
| MANSOURA UNIVERSITY | 2 | 0.079 |
| MASSACHUSETTS INSTITUTE OF TECHNOLOGY MIT | 2 | 0.079 |
| MASSEY UNIVERSITY | 2 | 0.079 |
| MAX PLANCK SOCIETY | 2 | 0.079 |
| MEDICAL COLLEGE OF WISCONSIN | 2 | 0.079 |
| MEDICAL UNIVERSITY OF GRAZ | 2 | 0.079 |
| MEDICAL UNIVERSITY SOFIA | 2 | 0.079 |
| METROPOLITAN STATE UNIVERSITY | 2 | 0.079 |
| MIAMI VA MED CTR | 2 | 0.079 |
| MICHIGAN STATE UNIVERSITY | 2 | 0.079 |
| MID SWEDEN UNIVERSITY | 2 | 0.079 |
| MIRIAM HOSPITAL | 2 | 0.079 |
| MOLINETTE MAURIZIANO HOSPITAL | 2 | 0.079 |
| MOVEMENT DISORDERS CTR | 2 | 0.079 |
| MURDOCH CHILDREN S RESEARCH INSTITUTE | 2 | 0.079 |
| NAGOYA CITY UNIVERSITY | 2 | 0.079 |
| NAGOYA UNIVERSITY | 2 | 0.079 |
| NANJING UNIVERSITY | 2 | 0.079 |
| NATIONAL AGEING RESEARCH INSTITUTE | 2 | 0.079 |
| NATIONAL CANCER INSTITUTE INCA | 2 | 0.079 |
| NATIONAL CHENG KUNG UNIVERSITY HOSPITAL | 2 | 0.079 |
| NATIONAL CHIAO TUNG UNIVERSITY | 2 | 0.079 |
| NATIONAL KAOHSIUNG NORMAL UNIVERSITY | 2 | 0.079 |
| NATL RES DEV CTR WELF HLTH | 2 | 0.079 |
| NEUROBIZ CONSULTING COMMUN | 2 | 0.079 |
| NEUROL INST C BESTA IRCCS FDN | 2 | 0.079 |
| NEUROPSYCHIAT HOSP DR IVAN BARBOT | 2 | 0.079 |
| NEUROSCI EDUC INST | 2 | 0.079 |
| NEVADA SYSTEM OF HIGHER EDUCATION NSHE | 2 | 0.079 |
| NEWTON WELLESLEY HOSPITAL | 2 | 0.079 |
| NIH NATIONAL CANCER INSTITUTE NCI | 2 | 0.079 |
| NORTHSHORE UNIVERSITY HEALTH SYSTEM | 2 | 0.079 |
| NORWEGIAN CTR VIOLENCE TRAUMAT STRESS STUDIES | 2 | 0.079 |
| NOVO NORDISK | 2 | 0.079 |
| NYNASHAMN REHABIL CTR | 2 | 0.079 |
| OPTUMINSIGHT | 2 | 0.079 |
| OTTO FRIEDRICH UNIV BAMBERG | 2 | 0.079 |
| PARDEE RAND GRADUATE SCHOOL | 2 | 0.079 |
| PARKINSONS CLIN EASTERN TORONTO | 2 | 0.079 |
| PAVLOV INSTITUTE OF PHYSIOLOGY RUSSIAN ACADEMY OF SCIENCES | 2 | 0.079 |
| PAYNE WHITNEY PSYCHIAT CLIN | 2 | 0.079 |
| PHILADELPHIA GERIATR CTR | 2 | 0.079 |
| PLA | 2 | 0.079 |
| POLIAMBULATORIO FISIOCTR | 2 | 0.079 |
| PONTIFICIA UNIVERSIDAD CATOLICA DE CHILE | 2 | 0.079 |
| PORTLAND STATE UNIVERSITY | 2 | 0.079 |
| PRAGUE PSYCHIAT CTR | 2 | 0.079 |
| PRIDE RES FDN | 2 | 0.079 |
| PRINCE CHARLES HOSPITAL | 2 | 0.079 |
| PRINCE WALES HOSP | 2 | 0.079 |
| PRO PERSONA | 2 | 0.079 |
| PURDUE PHARMA LP | 2 | 0.079 |
| PURDUE UNIVERSITY | 2 | 0.079 |
| PURDUE UNIVERSITY SYSTEM | 2 | 0.079 |
| QUEEN ELIZABETH HOSP | 2 | 0.079 |
| RAAHE HOSP | 2 | 0.079 |
| RAINBOW BABIES CHILDREN S HOSPITAL | 2 | 0.079 |
| REHABIL CTR SEEHOF | 2 | 0.079 |
| RES INST HEBREW HOME GREATER WASHINGTON | 2 | 0.079 |
| RHODE ISLAND HOSPITAL | 2 | 0.079 |
| RIKEN | 2 | 0.079 |
| ROCHE HOLDING | 2 | 0.079 |
| ROUDEBUSH VA CTR EXCELLENCE IMPLEMENTING EVIDENCE | 2 | 0.079 |
| ROYAL ADELAIDE HOSPITAL | 2 | 0.079 |
| ROYAL INSTITUTE OF TECHNOLOGY | 2 | 0.079 |
| ROYAL MELBOURNE INSTITUTE OF TECHNOLOGY RMIT | 2 | 0.079 |
| SAFAK HEALTHCARE GROUP | 2 | 0.079 |
| SAINT LUKE S HOSPITAL MISSOURI | 2 | 0.079 |
| SAMSUNG MEDICAL CENTER | 2 | 0.079 |
| SAN FRANCISCO VA MEDICAL CENTER | 2 | 0.079 |
| SAN GIUSEPPE MOSCATI HOSPITAL | 2 | 0.079 |
| SCHON KLIN HAMBURG EILBEK | 2 | 0.079 |
| SCUOLA NORMALE SUPERIORE DI PISA | 2 | 0.079 |
| SELCUK UNIVERSITY | 2 | 0.079 |
| SEOUL METROPOLITAN EUNPYEONG HOSP | 2 | 0.079 |
| SEOUL ST MARY S HOSPITAL | 2 | 0.079 |
| SHAARE ZEDEK MEDICAL CENTER | 2 | 0.079 |
| SHANDONG MENTAL HLTH CTR | 2 | 0.079 |
| SHANGHAI INST MENTAL HLTH | 2 | 0.079 |
| SHIRAZ UNIVERSITY OF MEDICAL SCIENCE | 2 | 0.079 |
| SINAI HOSPITAL OF BALTIMORE | 2 | 0.079 |
| SINT MAARTENS CLINIC | 2 | 0.079 |
| SOUTH LONDON MAUDSLEY NHS FDN TRUST | 2 | 0.079 |
| SOUTHDOWN INST | 2 | 0.079 |
| ST ANDREA HOSP | 2 | 0.079 |
| ST JOAN DE DEU TERRES LLEIDA | 2 | 0.079 |
| ST PETERSBURG SCIENTIFIC CENTRE OF THE RUSSIAN ACADEMY OF SCIENCES | 2 | 0.079 |
| STANLEY ST TREATMENT RESOURCES | 2 | 0.079 |
| STATE UNIVERSITY OF NEW YORK SUNY ALBANY | 2 | 0.079 |
| SUNGKYUNKWAN UNIVERSITY | 2 | 0.079 |
| SUNNYBROOK HEALTH SCIENCE CENTER | 2 | 0.079 |
| SUNNYBROOK RESEARCH INSTITUTE | 2 | 0.079 |
| TAIPEI CITY HOSPITAL | 2 | 0.079 |
| THOMAS JEFFERSON UNIVERSITY | 2 | 0.079 |
| TOHO UNIVERSITY | 2 | 0.079 |
| TOKAI UNIVERSITY | 2 | 0.079 |
| TOKYO SHINJUKU MED CTR | 2 | 0.079 |
| TOYO UNIVERSITY | 2 | 0.079 |
| TRAKYA UNIVERSITY | 2 | 0.079 |
| TUFTS MEDICAL CENTER | 2 | 0.079 |
| TULANE UNIVERSITY | 2 | 0.079 |
| UBE FRONTIER COLL | 2 | 0.079 |
| UIJEONGBU HOSP | 2 | 0.079 |
| UNIV BAMBERG | 2 | 0.079 |
| UNIV COLL IBADAN HOSP | 2 | 0.079 |
| UNIV FLENSBURG | 2 | 0.079 |
| UNIV HOSP ZURICH | 2 | 0.079 |
| UNIV LOUVAIN | 2 | 0.079 |
| UNIV MED CTR | 2 | 0.079 |
| UNIV ZULIA | 2 | 0.079 |
| UNIVERSIDAD DE EXTREMADURA | 2 | 0.079 |
| UNIVERSIDAD DE GUADALAJARA | 2 | 0.079 |
| UNIVERSIDAD DE JAEN | 2 | 0.079 |
| UNIVERSIDAD NACIONAL AUTONOMA DE MEXICO | 2 | 0.079 |
| UNIVERSIDADE DA CORUNA | 2 | 0.079 |
| UNIVERSIDADE DO MINHO | 2 | 0.079 |
| UNIVERSIDADE DO PORTO | 2 | 0.079 |
| UNIVERSIDADE ESTADUAL DE CAMPINAS | 2 | 0.079 |
| UNIVERSIDADE ESTADUAL PAULISTA | 2 | 0.079 |
| UNIVERSIDADE FEDERAL DE PELOTAS | 2 | 0.079 |
| UNIVERSIDADE FEDERAL DE SAO CARLOS | 2 | 0.079 |
| UNIVERSIDADE FEDERAL DO CEARA | 2 | 0.079 |
| UNIVERSIDADE FEDERAL DO RIO DE JANEIRO | 2 | 0.079 |
| UNIVERSITAT DE GIRONA | 2 | 0.079 |
| UNIVERSITAT DE LLEIDA | 2 | 0.079 |
| UNIVERSITAT ROVIRA I VIRGILI | 2 | 0.079 |
| UNIVERSITAT TRIER | 2 | 0.079 |
| UNIVERSITE DE NIMES | 2 | 0.079 |
| UNIVERSITE LIBRE DE BRUXELLES | 2 | 0.079 |
| UNIVERSITE SORBONNE PARIS CITE USPC COMUE | 2 | 0.079 |
| UNIVERSITY COLLEGE DUBLIN | 2 | 0.079 |
| UNIVERSITY HOSPITAL SAPIENZA ROME | 2 | 0.079 |
| UNIVERSITY OF AQUILA | 2 | 0.079 |
| UNIVERSITY OF BARI | 2 | 0.079 |
| UNIVERSITY OF BUENOS AIRES | 2 | 0.079 |
| UNIVERSITY OF CAGLIARI | 2 | 0.079 |
| UNIVERSITY OF CALIFORNIA LOS ANGELES MEDICAL CENTER | 2 | 0.079 |
| UNIVERSITY OF CHESTER | 2 | 0.079 |
| UNIVERSITY OF CHICAGO MEDICAL CENTER | 2 | 0.079 |
| UNIVERSITY OF COLOGNE | 2 | 0.079 |
| UNIVERSITY OF EXETER | 2 | 0.079 |
| UNIVERSITY OF FERRARA | 2 | 0.079 |
| UNIVERSITY OF FOGGIA | 2 | 0.079 |
| UNIVERSITY OF FRIBOURG | 2 | 0.079 |
| UNIVERSITY OF HAWAII MANOA | 2 | 0.079 |
| UNIVERSITY OF HERTFORDSHIRE | 2 | 0.079 |
| UNIVERSITY OF ICELAND | 2 | 0.079 |
| UNIVERSITY OF INDONESIA | 2 | 0.079 |
| UNIVERSITY OF JYVASKYLA | 2 | 0.079 |
| UNIVERSITY OF KIEL | 2 | 0.079 |
| UNIVERSITY OF KWAZULU NATAL | 2 | 0.079 |
| UNIVERSITY OF LUBECK | 2 | 0.079 |
| UNIVERSITY OF MAINE SYSTEM | 2 | 0.079 |
| UNIVERSITY OF MASSACHUSETTS AMHERST | 2 | 0.079 |
| UNIVERSITY OF MISSOURI KANSAS CITY | 2 | 0.079 |
| UNIVERSITY OF NEBRASKA LINCOLN | 2 | 0.079 |
| UNIVERSITY OF NEBRASKA MEDICAL CENTER | 2 | 0.079 |
| UNIVERSITY OF NEVADA LAS VEGAS | 2 | 0.079 |
| UNIVERSITY OF NEW HAMPSHIRE | 2 | 0.079 |
| UNIVERSITY OF PUERTO RICO MEDICAL SCIENCES CAMPUS | 2 | 0.079 |
| UNIVERSITY OF REGINA | 2 | 0.079 |
| UNIVERSITY OF SAN DIEGO | 2 | 0.079 |
| UNIVERSITY OF SEVILLA | 2 | 0.079 |
| UNIVERSITY OF SPLIT | 2 | 0.079 |
| UNIVERSITY OF TECHNOLOGY SYDNEY | 2 | 0.079 |
| UNIVERSITY OF TENNESSEE SYSTEM | 2 | 0.079 |
| UNIVERSITY OF TEXAS RIO GRANDE VALLEY | 2 | 0.079 |
| UNIVERSITY OF TSUKUBA | 2 | 0.079 |
| UNIVERSITY OF VICTORIA | 2 | 0.079 |
| UNIVERSITY OF WATERLOO | 2 | 0.079 |
| UNIVERSITY OF WURZBURG | 2 | 0.079 |
| UNIVERSITY OF YORK UK | 2 | 0.079 |
| UNIVERSITY QUEBEC ABITIBI TEMISCAMINGUE | 2 | 0.079 |
| UNIVERSITY SYSTEM OF NEW HAMPSHIRE | 2 | 0.079 |
| VESTFOLD HOSP | 2 | 0.079 |
| VET AFFAIRS PUGET SOUND HEALTH CARE SYSTEM | 2 | 0.079 |
| VET GEN HOSP | 2 | 0.079 |
| VRIJE UNIVERSITEIT BRUSSEL | 2 | 0.079 |
| WENZHOU MEDICAL UNIVERSITY | 2 | 0.079 |
| WEST VIRGINIA UNIVERSITY | 2 | 0.079 |
| WESTERN SYDNEY UNIVERSITY | 2 | 0.079 |
| WESTSIDE FAMILY MED CTR | 2 | 0.079 |
| WOMENS COLLEGE HOSPITAL | 2 | 0.079 |
| WONKWANG UNIVERSITY | 2 | 0.079 |
| WROCLAW MEDICAL UNIVERSITY | 2 | 0.079 |
| XIDIAN UNIVERSITY | 2 | 0.079 |
| YAMAGUCHI UNIVERSITY | 2 | 0.079 |
| YEDITEPE UNIVERSITY | 2 | 0.079 |
| YOZGAT SORGUN STATE HOSPITAL | 2 | 0.079 |
| YUZUNCU YIL UNIVERSITY | 2 | 0.079 |
| ZHENGZHOU UNIVERSITY | 2 | 0.079 |
| 23ANDME INC | 1 | 0.04 |
| 251 GEN AF HOSP | 1 | 0.04 |
| 2ND IKA HOSP | 1 | 0.04 |
| 2ND XIANGYA HOSP | 1 | 0.04 |
| 4TH MIL TEACHING HOSP | 1 | 0.04 |
| A A BOGOMOLETZ INSTITUTE OF PHYSIOLOGY | 1 | 0.04 |
| A O U CITTA DELLA SALUTE E DELLA SCIENZA DI TORINO | 1 | 0.04 |
| ABBOT NW HOSP | 1 | 0.04 |
| ABINGDON HOSP | 1 | 0.04 |
| ABT BIOPHARM SOLUT INC | 1 | 0.04 |
| ACAD HOSP CHU NIMES | 1 | 0.04 |
| ACADEMIA SINICA TAIWAN | 1 | 0.04 |
| ACADEMIC CENTER FOR DENTISTRY AMSTERDAM | 1 | 0.04 |
| ACASTER CONSULTING | 1 | 0.04 |
| ACCELERATED COMMUNITY ONCOL RES NETWORK | 1 | 0.04 |
| ACO LAKEMEDEL AB | 1 | 0.04 |
| ADELANTE CTR EXPERTISE REHABIL AUDIOL | 1 | 0.04 |
| ADELPHI UNIVERSITY | 1 | 0.04 |
| ADNAN MENDERES UNIVERSITY | 1 | 0.04 |
| ADV PAIN MANAGEMENT REHAB GRP INC | 1 | 0.04 |
| AESTHET MIND CLIN | 1 | 0.04 |
| AFYONKARAHISAR STATE HOSPITAL | 1 | 0.04 |
| AGENCY FOR HEALTHCARE RESEARCH QUALITY | 1 | 0.04 |
| AGIOS DIMITRIOS HOSP | 1 | 0.04 |
| AHI EVRAN UNIVERSITY | 1 | 0.04 |
| AHQR | 1 | 0.04 |
| AICHI MEDICAL UNIVERSITY | 1 | 0.04 |
| AIX MARSEILLE UNIVERSITE | 1 | 0.04 |
| AJOU UNIVERSITY | 1 | 0.04 |
| AKITA UNIVERSITY | 1 | 0.04 |
| AL AHLI HOSP | 1 | 0.04 |
| ALABAMA A M UNIVERSITY | 1 | 0.04 |
| ALBERTA INNOVATES HLTH SCI FDN | 1 | 0.04 |
| ALDER HEY CHILDREN S NHS FOUNDATION TRUST | 1 | 0.04 |
| ALEXANDRU IOAN CUZA UNIVERSITY | 1 | 0.04 |
| ALEXIANER HOSP KREFELD | 1 | 0.04 |
| ALEXIANER KRANKENHAUS KREFELD | 1 | 0.04 |
| ALFRED HLTH | 1 | 0.04 |
| ALL INDIA INSTITUTE OF MEDICAL SCIENCES | 1 | 0.04 |
| ALLEGHENY UNIV GRAD HOSP | 1 | 0.04 |
| ALLERGAN | 1 | 0.04 |
| ALLIANT UNIV | 1 | 0.04 |
| ALRIJNE HOSP | 1 | 0.04 |
| ALTARUM INST | 1 | 0.04 |
| AMC CANCER RESEARCH CENTER | 1 | 0.04 |
| AMER HLTH NETWORK | 1 | 0.04 |
| AMER INT GRP | 1 | 0.04 |
| AMERICAN COLL PHYS | 1 | 0.04 |
| AMERICAN UNIVERSITY OF BEIRUT | 1 | 0.04 |
| AMSTERDAM REHABIL RES CTR READE | 1 | 0.04 |
| ANADOLU UNIVERSITY | 1 | 0.04 |
| ANAL GRP | 1 | 0.04 |
| ANALGES SOLUT | 1 | 0.04 |
| ANDALUSIA HLTH SERV | 1 | 0.04 |
| ANDALUSIAN CENTER FOR DEVELOPMENTAL BIOLOGY CABD | 1 | 0.04 |
| ANKARA UNIVERSITY | 1 | 0.04 |
| ANKARA YENIMAHALLE TRAINING RESEARCH HOSPITAL | 1 | 0.04 |
| ANN ROBERT H LURIE CHILDREN S HOSPITAL OF CHICAGO | 1 | 0.04 |
| ANTALYA TRAINING RESEARCH HOSPITAL | 1 | 0.04 |
| ANXIETY TREATMENT RES CTR | 1 | 0.04 |
| AO BKH KUFSTEIN | 1 | 0.04 |
| APPL INNOVAT PSYCHIAT | 1 | 0.04 |
| ARAMIS | 1 | 0.04 |
| ARKANSAS CHILDRENS NUTR CTR | 1 | 0.04 |
| ARMED FORCES CAPITAL HOSP | 1 | 0.04 |
| ARMED FORCES DENT HOSP | 1 | 0.04 |
| ARMY MEDICAL UNIVERSITY | 1 | 0.04 |
| ARTHRIT RES CTR CANADA | 1 | 0.04 |
| ASIA UNIVERSITY TAIWAN | 1 | 0.04 |
| ASKLEPIOS MED CTR BAD ABBACH | 1 | 0.04 |
| ASL 13 | 1 | 0.04 |
| ASL 2 | 1 | 0.04 |
| ASL 4 | 1 | 0.04 |
| ASOCIAC ESPANOLA PACIENTES CEFALEA AEPAC | 1 | 0.04 |
| ASOCIAC PSICOANALIT MEXICANA | 1 | 0.04 |
| ASSISTANCE PUBLIQUE HOPITAUX DE MARSEILLE | 1 | 0.04 |
| ASST FATEBENEFRATELLI L SACCO UNIV HOSP | 1 | 0.04 |
| ASTELLAS PHARMACEUTICALS | 1 | 0.04 |
| ATHENS NAVAL VET HOSP | 1 | 0.04 |
| ATKINSON MORLEYS HOSP | 1 | 0.04 |
| ATLANTA VA HEALTH CARE SYSTEM | 1 | 0.04 |
| AUBURN UNIVERSITY | 1 | 0.04 |
| AUBURN UNIVERSITY SYSTEM | 1 | 0.04 |
| AUDIE L MURPHY MEMORIAL VETERANS HOSPITAL | 1 | 0.04 |
| AUSL REGGIO EMILIA | 1 | 0.04 |
| AUSTEN BIOINNOVAT INST AKRON | 1 | 0.04 |
| AUSTRALIAN CATHOLIC UNIVERSITY | 1 | 0.04 |
| AUSTRALIAN COLL APPL PSYCHOL | 1 | 0.04 |
| AUSTRALIAN NATIONAL UNIVERSITY | 1 | 0.04 |
| AUSTRALIAN PSYCHOL SOC | 1 | 0.04 |
| AVALON HLTH SOLUT INC | 1 | 0.04 |
| AVICENNA HOSPITAL | 1 | 0.04 |
| AVON ORTHOPAED CTR | 1 | 0.04 |
| AZ OSP PROV LECCO | 1 | 0.04 |
| AZIENDA OSPED POLICLIN BARI | 1 | 0.04 |
| BACK PAIN CTR | 1 | 0.04 |
| BAHIA SCH MED PUBL HLTH | 1 | 0.04 |
| BAHIANA SCH MED PUBL HLTH | 1 | 0.04 |
| BAKER HEART AND DIABETES INSTITUTE | 1 | 0.04 |
| BALEAR ISL HLTH RES INST IDISBA | 1 | 0.04 |
| BALTIMORE VA MEDICAL CENTER | 1 | 0.04 |
| BAQAI MEDICAL UNIVERSITY | 1 | 0.04 |
| BAQIYATALLAH HOSP | 1 | 0.04 |
| BAQIYATALLAH UNIVERSITY OF MEDICAL SCIENCES BMSU | 1 | 0.04 |
| BARON CTR INC | 1 | 0.04 |
| BARROW NEUROLOGICAL INSTITUTE | 1 | 0.04 |
| BASKENT UNIVERSITY | 1 | 0.04 |
| BASURTO HOSPITAL | 1 | 0.04 |
| BATEMAN HORNE CTR | 1 | 0.04 |
| BAYER AG | 1 | 0.04 |
| BAYER HEALTHCARE PHARMACEUTICALS | 1 | 0.04 |
| BAYINDIR HASTANESI | 1 | 0.04 |
| BAYSTATE MEDICAL CENTER | 1 | 0.04 |
| BC WOMENS CHILDRENS HOSP | 1 | 0.04 |
| BEATRIZ ANGELO HOSP | 1 | 0.04 |
| BEAUMONT HOSP | 1 | 0.04 |
| BEAVER MED GRP | 1 | 0.04 |
| BEHAV MED NETWORK | 1 | 0.04 |
| BEIJING NORMAL UNIVERSITY | 1 | 0.04 |
| BELLVITGE UNIVERSITY HOSPITAL | 1 | 0.04 |
| BENEMERITA UNIVERSIDAD AUTONOMA DE PUEBLA | 1 | 0.04 |
| BERGEN PINES CTY HOSP | 1 | 0.04 |
| BHAYANGKARA HOSP | 1 | 0.04 |
| BIOENGN MED INFORMAT CONSORTIUM | 1 | 0.04 |
| BIRLA INSTITUTE OF TECHNOLOGY AND SCIENCE PILANI BITS PILANI | 1 | 0.04 |
| BIRMINGHAM MIDLAND EYE HOSP | 1 | 0.04 |
| BIRMINGHAM MIGRAINE CLIN | 1 | 0.04 |
| BISHOPS UNIVERSITY | 1 | 0.04 |
| BLUE RIDGE HEADACHE CTR | 1 | 0.04 |
| BOOTS FORMENTI SPA | 1 | 0.04 |
| BOURNEMOUTH UNIVERSITY | 1 | 0.04 |
| BRAIN RES NEW ZEALAND | 1 | 0.04 |
| BRESSANONE HOSP | 1 | 0.04 |
| BRIGHAM YOUNG UNIVERSITY | 1 | 0.04 |
| BRITISH COLUMBIA INST TECHNOL | 1 | 0.04 |
| BRITISH SCH OSTEOPATHY | 1 | 0.04 |
| BROAD INSTITUTE | 1 | 0.04 |
| BROCK UNIVERSITY | 1 | 0.04 |
| BRONX PSYCHIAT CTR | 1 | 0.04 |
| BROOKWOOD MED CTR | 1 | 0.04 |
| BUDAPEST UNIVERSITY OF TECHNOLOGY ECONOMICS | 1 | 0.04 |
| BUFFALO STATE COLLEGE | 1 | 0.04 |
| BUHARA MEDICAL CENTER | 1 | 0.04 |
| BUR BUSINESS ECON RES | 1 | 0.04 |
| BURWOOD HOSP | 1 | 0.04 |
| C BESTA NEUROL INST FDN | 1 | 0.04 |
| CABINET MED SPECIALISE ETATS ESPRIT | 1 | 0.04 |
| CABRINI HEALTH | 1 | 0.04 |
| CADWGAN SURG | 1 | 0.04 |
| CALIFORNIA PACIFIC MEDICAL CENTER | 1 | 0.04 |
| CAMPBELL UNIVERSITY | 1 | 0.04 |
| CANADIAN INSTITUTE FOR ADVANCED RESEARCH CIFAR | 1 | 0.04 |
| CANANDAIGUA VET ADM MED CTR | 1 | 0.04 |
| CANBERRA SURGICTR | 1 | 0.04 |
| CANCER RESEARCH UK | 1 | 0.04 |
| CAPITAL REG DENMARK | 1 | 0.04 |
| CAREWEST OPERAT STRESS INJURY CLIN | 1 | 0.04 |
| CARL GUSTAV CARUS UNIVERSITY HOSPITAL | 1 | 0.04 |
| CARL VON OSSIETZKY UNIVERSITAT OLDENBURG | 1 | 0.04 |
| CARLETON UNIVERSITY | 1 | 0.04 |
| CARMEL MEDICAL CENTER | 1 | 0.04 |
| CAROLINAS MED CTR | 1 | 0.04 |
| CATALPINAR SITESI | 1 | 0.04 |
| CATHAY GENERAL HOSPITAL | 1 | 0.04 |
| CATHOLIC UNIVERSITY KOREA HOSPITAL | 1 | 0.04 |
| CATHOLIC UNIVERSITY OF DAEGU | 1 | 0.04 |
| CECIL G SHEPS CTR HLTH SERV RES | 1 | 0.04 |
| CEDOC HEADACHE CTR | 1 | 0.04 |
| CELAL BAYAR UNIVERSITY | 1 | 0.04 |
| CELENUS KLINIKEN GMBH | 1 | 0.04 |
| CENT HOSP SHAOYANG | 1 | 0.04 |
| CENTERS FOR DISEASE CONTROL PREVENTION USA | 1 | 0.04 |
| CENTRAL ARKANSAS VETERANS HEALTHCARE SYSTEM | 1 | 0.04 |
| CENTRAL CONNECTICUT STATE UNIVERSITY | 1 | 0.04 |
| CENTRAL INSTITUTE OF PSYCHIATRY | 1 | 0.04 |
| CENTRE OF POSTGRADUATE MEDICAL EDUCATION POLAND | 1 | 0.04 |
| CENTRO DI RIFERIMENTO PER EPIDEMIOLOGIA E LA PREVENZIONE ONCOLOGICA IN PIEMONTE | 1 | 0.04 |
| CHARLES STURT UNIVERSITY | 1 | 0.04 |
| CHATHAM COLL | 1 | 0.04 |
| CHENGDU UNIVERSITY OF TECHNOLOGY | 1 | 0.04 |
| CHI MEI HOSPITAL | 1 | 0.04 |
| CHIA NAN UNIVERSITY OF PHARMACY SCIENCE | 1 | 0.04 |
| CHIA YI CHRISTIAN HOSP | 1 | 0.04 |
| CHIANG MAI UNIVERSITY | 1 | 0.04 |
| CHIBA UNIVERSITY | 1 | 0.04 |
| CHILDREN S HOSPITAL LOS ANGELES | 1 | 0.04 |
| CHILDREN S MEMORIAL HEALTH INSTITUTE | 1 | 0.04 |
| CHILDREN S MERCY HOSPITAL | 1 | 0.04 |
| CHILDRENS HOSP CHICAGO | 1 | 0.04 |
| CHILDRENS HOSP PHILADELPHIA | 1 | 0.04 |
| CHILDRENS HOSP ZHENGZHOU | 1 | 0.04 |
| CHILDRENS NATL MED CTR | 1 | 0.04 |
| CHINA MEDICAL UNIVERSITY | 1 | 0.04 |
| CHINA PHARMACEUTICAL UNIVERSITY | 1 | 0.04 |
| CHINESE ACADEMY OF MEDICAL SCIENCES PEKING UNION MEDICAL COLLEGE | 1 | 0.04 |
| CHINESE PEOPLES LIBERAT ARMY | 1 | 0.04 |
| CHODANG UNIV | 1 | 0.04 |
| CHONNAM NATIONAL UNIVERSITY | 1 | 0.04 |
| CHOSUN UNIVERSITY | 1 | 0.04 |
| CHR METZ THIONVILLE | 1 | 0.04 |
| CHRISTCHURCH HOSP | 1 | 0.04 |
| CHRISTIAN DOPPLER KLIN SALZBURG | 1 | 0.04 |
| CHU BORDEAUX | 1 | 0.04 |
| CHU LIMOGES | 1 | 0.04 |
| CHUNG YUAN CHRISTIAN UNIVERSITY | 1 | 0.04 |
| CHURCHILL HOSP | 1 | 0.04 |
| CHUSHIN MATSUMOTO HOSP | 1 | 0.04 |
| CIBERBBN | 1 | 0.04 |
| CIBEREHD | 1 | 0.04 |
| CIBERESP | 1 | 0.04 |
| CINVESTAV CENTRO DE INVESTIGACION Y DE ESTUDIOS AVANZADOS DEL INSTITUTO POLITECNICO NACIONAL | 1 | 0.04 |
| CITTA SALUTE SCI HOSP TURIN | 1 | 0.04 |
| CITY COLLEGE OF NEW YORK CUNY | 1 | 0.04 |
| CITY HLTH SERV DEBRECEN | 1 | 0.04 |
| CITY LONDON MIGRAINE CLIN | 1 | 0.04 |
| CITY UNIVERSITY LONDON | 1 | 0.04 |
| CLALIT HEALTH SERVICES | 1 | 0.04 |
| CLALIT RES INST | 1 | 0.04 |
| CLARIVATE ANALYTICS | 1 | 0.04 |
| CLIFTON HOSP | 1 | 0.04 |
| CLIN A RUE MCGILL | 1 | 0.04 |
| CLIN BARMELWEID | 1 | 0.04 |
| CLIN NEUROL PSYCHIAT CHILDREN YOUTH | 1 | 0.04 |
| CLIN NEUROPSYCHIAT | 1 | 0.04 |
| CLIN OUTCOMES SOLUT | 1 | 0.04 |
| CLIN PSYCHIAT PSYCHOTHERAPY GIESSEN | 1 | 0.04 |
| CLIN RES CTR | 1 | 0.04 |
| CLIN ROMANDE READAPTAT SUVACARE | 1 | 0.04 |
| CLIN SALUDCOOP NEIVA | 1 | 0.04 |
| CLIN STUDY SUPPORT INC | 1 | 0.04 |
| CLIN TRANSLAT SCI INST | 1 | 0.04 |
| CLIN TRIALS EPIDEMIOL RES UNIT | 1 | 0.04 |
| CLS 649 | 1 | 0.04 |
| CNRS NATIONAL INSTITUTE FOR BIOLOGY INSB | 1 | 0.04 |
| CNS CLIN RES GRP | 1 | 0.04 |
| CNS RES FDN INC | 1 | 0.04 |
| CODMAN SQ HLTH CTR | 1 | 0.04 |
| COLL ACUPUNCTURE MOXIBUST TUINA | 1 | 0.04 |
| COLL MED | 1 | 0.04 |
| COLL MT ST VINCENT | 1 | 0.04 |
| COLL N TROENDELAG | 1 | 0.04 |
| COLL PHARM | 1 | 0.04 |
| COLLEGE OF CHARLESTON | 1 | 0.04 |
| COLLEGIUM MEDICUM JAGIELLONIAN UNIVERSITY | 1 | 0.04 |
| COLUMBIA UNIVERSITY TEACHERS COLLEGE | 1 | 0.04 |
| COMENIUS UNIVERSITY BRATISLAVA | 1 | 0.04 |
| COMM RELIGIOUS SPIRITUAL ASSISTANCE CARE | 1 | 0.04 |
| COMMUN HLTH CARE PLAN | 1 | 0.04 |
| COMMUNAUTE D UNIVERSITES ET ETABLISSEMENTS D AQUITAINE COMUE | 1 | 0.04 |
| COMMUNITY HOME HLTH HOSP | 1 | 0.04 |
| COMMUNITY HOME HLTH HOSP SYMPTOM MANAGEMENT GRP | 1 | 0.04 |
| COMMUNITY HOSP HLTH CARE SYST | 1 | 0.04 |
| COMPLEXO HOSPITALARIO UNIVERSITARIO DE SANTIAGO DE COMPOSTELA | 1 | 0.04 |
| CONCORD HOSPITAL | 1 | 0.04 |
| CONCORD REPATRIATION GENERAL HOSPITAL | 1 | 0.04 |
| CONNECTICUT STATE UNIVERSITY SYSTEM | 1 | 0.04 |
| CONSEJO NACIONAL DE INVESTIGACIONES CIENTIFICAS Y TECNICAS CONICET | 1 | 0.04 |
| CONSULTANTS BEHAV RES | 1 | 0.04 |
| COPENHAGEN BUSINESS SCHOOL | 1 | 0.04 |
| CORK REG HOSP | 1 | 0.04 |
| CORNWALL PARTNERSHIP NHS FDN TRUST | 1 | 0.04 |
| CORREOS GRP VALENCIA | 1 | 0.04 |
| CORTEX PHARMACEUT | 1 | 0.04 |
| CREIGHTON UNIVERSITY | 1 | 0.04 |
| CSIC INSTITUTO DE BIOMEDICINA Y BIOTECNOLOGIA DE CANTABRIA IBBTEC | 1 | 0.04 |
| CSIC UMH INSTITUTO DE NEUROCIENCIAS DE ALICANTE IN | 1 | 0.04 |
| CTO HOSP | 1 | 0.04 |
| CTR ACT WORK DISABIL PREVENT REHABIL | 1 | 0.04 |
| CTR ANXIETY DEPRESS | 1 | 0.04 |
| CTR ATENCIO PRIMARIA CONSTANTI | 1 | 0.04 |
| CTR CEFALEE OPSED G SALVINI | 1 | 0.04 |
| CTR CEFALEE OSPED G SALVINI | 1 | 0.04 |
| CTR CHILD ADOLESCENT MENTAL HLTH EASTERN SOUT | 1 | 0.04 |
| CTR CLIN STUDIES | 1 | 0.04 |
| CTR COGNIT PSYCHOTHERAPY EDUC | 1 | 0.04 |
| CTR COMPREHENS CARE DIAG INHERITED BLEEDING DIS | 1 | 0.04 |
| CTR EXCELLENCE ENVIRONM MED | 1 | 0.04 |
| CTR FAMILY MED | 1 | 0.04 |
| CTR HLTH TECHNOL SERV RES CINTESIS UA | 1 | 0.04 |
| CTR HOSP ESQUIROL | 1 | 0.04 |
| CTR HOSP PAYS DAVESNES | 1 | 0.04 |
| CTR INNOVAT QUAL EFFECTIVENESS SAFETY | 1 | 0.04 |
| CTR LIFE SCI | 1 | 0.04 |
| CTR LOCAL SERV COMMUNAUTAIRES METRO CLSC METRO | 1 | 0.04 |
| CTR MICHOACANO SALUD MENTAL | 1 | 0.04 |
| CTR OUTCOMES RES EVALUAT | 1 | 0.04 |
| CTR PUBL MENTAL HLTH | 1 | 0.04 |
| CTR REPROD HUMANA CAMPINAS | 1 | 0.04 |
| CTR RES DEV MATH APPLICAT CIDMA | 1 | 0.04 |
| CTR RHEUMATOL REHABIL | 1 | 0.04 |
| CTR SANTE SERV SOCIAUX MONTAGNE | 1 | 0.04 |
| CTR UNIV FUNDACAO EDUC GUAXUPE UNIFEG | 1 | 0.04 |
| CURTIN UNIVERSITY | 1 | 0.04 |
| CYBERON | 1 | 0.04 |
| CZECH ACADEMY OF SCIENCES | 1 | 0.04 |
| DAEGU UNIVERSITY | 1 | 0.04 |
| DAK LAB AS | 1 | 0.04 |
| DANDERYDS HOSPITAL | 1 | 0.04 |
| DANKOOK UNIVERSITY | 1 | 0.04 |
| DAQING GRP OILFIELD GEN HOSP | 1 | 0.04 |
| DAVID TVILDIANI MED UNIV | 1 | 0.04 |
| DEFENCE RESEARCH DEVELOPMENT ESTABLISHMENT DRDE | 1 | 0.04 |
| DEFENCE RESEARCH DEVELOPMENT ORGANISATION DRDO | 1 | 0.04 |
| DELFT UNIVERSITY OF TECHNOLOGY | 1 | 0.04 |
| DENIZLI STATE HOSPITAL | 1 | 0.04 |
| DEPAUL UNIVERSITY | 1 | 0.04 |
| DEPT ANAT | 1 | 0.04 |
| DEPT ANESTHESIOL PERIOPERAT MED | 1 | 0.04 |
| DEPT CLIN PHARM | 1 | 0.04 |
| DEPT HLTH | 1 | 0.04 |
| DEPT NURSING HOME MED | 1 | 0.04 |
| DEPT PEDIAT | 1 | 0.04 |
| DEPT PHYS MED REHABIL | 1 | 0.04 |
| DEPT PSYCHIAT BEHAV MED SPECTRUM HLTH SYST | 1 | 0.04 |
| DEPT RES DEV | 1 | 0.04 |
| DESERT MED GRP | 1 | 0.04 |
| DEUTSCHES RHEUMA FORSCHUNGSZENTRUM DRFZ | 1 | 0.04 |
| DIABET PREVENT CONTROL CTR | 1 | 0.04 |
| DIACOR LTD | 1 | 0.04 |
| DIAKONHJEMMET HOSPITAL | 1 | 0.04 |
| DIAMOND HEADACHE CLIN LTD | 1 | 0.04 |
| DISKAPI YILDIRIM BEYAZIT TRAINING RESEARCH HOSPITAL | 1 | 0.04 |
| DIYARBAKIR TRAINING RESEARCH HOSPITAL | 1 | 0.04 |
| DOLEYS CLIN | 1 | 0.04 |
| DONGUK UNIV | 1 | 0.04 |
| DORSET HEALTHCARE UNIV NHS FDN TRUST | 1 | 0.04 |
| DOTHOUSE HLTH CTR | 1 | 0.04 |
| DOUGLAS GARDENS HOSP | 1 | 0.04 |
| DOUGLAS MENTAL HLTH UNIV INST | 1 | 0.04 |
| DR ABDURRAHMAN YURTASLAN ONCOLOGY HOSPITAL | 1 | 0.04 |
| DR JOSIP BENCEVIC GEN HOSP | 1 | 0.04 |
| DR PIFARRE FDN | 1 | 0.04 |
| DUBLIN CITY UNIVERSITY | 1 | 0.04 |
| DUKE NUS MED SCH | 1 | 0.04 |
| DURHAM UNIVERSITY | 1 | 0.04 |
| DYSTROPH EPIDERMOLYSIS BULLOSA RES ASSOC MEXICO F | 1 | 0.04 |
| EAST LIMBURG HOSPITAL | 1 | 0.04 |
| EASTERN SOUTHERN NORWAY | 1 | 0.04 |
| EBMSP | 1 | 0.04 |
| ECAMPUS UNIV | 1 | 0.04 |
| ECON INC | 1 | 0.04 |
| EDGE HILL UNIVERSITY | 1 | 0.04 |
| EDITH NOURSE ROGERS MEM HOSP | 1 | 0.04 |
| EDMONTON GEN HOSP | 1 | 0.04 |
| EDWARD HINES JR VA HOSPITAL | 1 | 0.04 |
| EGE UNIVERSITY | 1 | 0.04 |
| EIRL | 1 | 0.04 |
| ELAN PHARMACEUT INC | 1 | 0.04 |
| EMORY BRAIN HLTH CTR | 1 | 0.04 |
| ENDO PHARMACEUT INC | 1 | 0.04 |
| ENVIRONM RES INFORMAT INC | 1 | 0.04 |
| EOTVOS LORAND UNIVERSITY | 1 | 0.04 |
| EPIDEMIOL RESOURCES INC | 1 | 0.04 |
| EPITECH GRP SRL | 1 | 0.04 |
| ERENKOY MENTAL NEUROLOGICAL DISORDERS EDUCATION RESEARCH HOSPITAL | 1 | 0.04 |
| ERZINCAN MILITARY HOSPITAL | 1 | 0.04 |
| ERZURUM BOLGE TRAINING RESEARCH HOSPITAL | 1 | 0.04 |
| ESCOLA SUPER CIENCIAS SAUDE VITORIA | 1 | 0.04 |
| ESTRELLA MT COMMUNITY COLL | 1 | 0.04 |
| EUROGENETICS | 1 | 0.04 |
| EUROPEAN HLTH OUTCOMES RES | 1 | 0.04 |
| EUROPEAN LEAGUE PAIN | 1 | 0.04 |
| EUROPEAN UNIV ROME | 1 | 0.04 |
| EVANGEL HOSP UNNA | 1 | 0.04 |
| EVANGEL UNIV | 1 | 0.04 |
| EVANGELISMOS HOSPITAL | 1 | 0.04 |
| FAC CATOLICA RAINHA SERTAO | 1 | 0.04 |
| FAC CIENCIAS DEPORTE | 1 | 0.04 |
| FAC CIENCIAS SALUD | 1 | 0.04 |
| FAC HLTH SCI | 1 | 0.04 |
| FAC MED SAO JOSE DO RIO PRETO | 1 | 0.04 |
| FAIRFAX HOSP | 1 | 0.04 |
| FATIMA MEM HOSP | 1 | 0.04 |
| FDN C BESTA | 1 | 0.04 |
| FDN COMBATING NEUROL DIS CHILDHOOD | 1 | 0.04 |
| FDN EPIGENET MED | 1 | 0.04 |
| FDN KOVACS | 1 | 0.04 |
| FDN PAOLO PROCACCI | 1 | 0.04 |
| FDN PISANA SCI ONLUS | 1 | 0.04 |
| FED UNIV HLTH SCI PORTO ALEGRE | 1 | 0.04 |
| FINNISH BROADCASTING CO | 1 | 0.04 |
| FIRAT UNIVERSITY | 1 | 0.04 |
| FLORIDA A M UNIVERSITY | 1 | 0.04 |
| FMC POLAND | 1 | 0.04 |
| FOCUS THERAPEUT OUTCOMES | 1 | 0.04 |
| FOCUS THERAPEUT OUTCOMES INC | 1 | 0.04 |
| FOLKHALSAN INST GENET | 1 | 0.04 |
| FOREST INST PROFESS PSYCHOL | 1 | 0.04 |
| FOREST RES INST INC | 1 | 0.04 |
| FORIPSI | 1 | 0.04 |
| FORREST GEN HOSP | 1 | 0.04 |
| FOUNTAIN ANALYT | 1 | 0.04 |
| FRANZ TAPPEINER HOSP | 1 | 0.04 |
| FRAY ANTONIO ALCALDE OPD | 1 | 0.04 |
| FRED HUTCHINSON CANCER CENTER | 1 | 0.04 |
| FREDERIKSBERG UNIV HOSP | 1 | 0.04 |
| FREMANTLE HOSP | 1 | 0.04 |
| FRISKVERNKLINIKKEN | 1 | 0.04 |
| FUNDACAO EDUCAC FERNANDOPOLIS | 1 | 0.04 |
| FUNDACIO ST JOAN DE DEU | 1 | 0.04 |
| G D ANNUNZIO UNIVERSITY OF CHIETI PESCARA | 1 | 0.04 |
| GACHON UNIVERSITY | 1 | 0.04 |
| GADJAH MADA UNIVERSITY | 1 | 0.04 |
| GAIA INC | 1 | 0.04 |
| GARDEN STATE INFECT DIS ASSOC | 1 | 0.04 |
| GCPSIRO | 1 | 0.04 |
| GEDEON RICHTER CHEMISTRY WORKS | 1 | 0.04 |
| GEM CLIN RES CONSULTING | 1 | 0.04 |
| GEN HOSP | 1 | 0.04 |
| GENDREAU CONSULTING | 1 | 0.04 |
| GENERAL UNIVERSITY HOSPITAL PRAGUE | 1 | 0.04 |
| GEORGE E WAHLEN VAMC | 1 | 0.04 |
| GEORGE FAY YEE CTR HEALTHCARE INNOVAT | 1 | 0.04 |
| GEORGE PAPANIKOLAOU GENERAL HOSPITAL OF THESSALONIKI | 1 | 0.04 |
| GEORGIA SOUTHERN UNIVERSITY | 1 | 0.04 |
| GERIATR ADULT PSYCHIAT LLC | 1 | 0.04 |
| GERIATR CARDIOL FDN | 1 | 0.04 |
| GERMAN PENS INSURANCE CO OLDENBURG BREMEN | 1 | 0.04 |
| GERMAN SPORT UNIV COLOGNE | 1 | 0.04 |
| GGNET | 1 | 0.04 |
| GGZ BREBURG TILBURG | 1 | 0.04 |
| GGZ INGEEST VU MED CTR | 1 | 0.04 |
| GHENT UNIVERSITY HOSPITAL | 1 | 0.04 |
| GI CARE KIDS | 1 | 0.04 |
| GIRONA UNIVERSITY HOSPITAL DR JOSEP TRUETA | 1 | 0.04 |
| GLOUCESTERSHIRE ROYAL HOSP | 1 | 0.04 |
| GOALISTICS LLC | 1 | 0.04 |
| GOLD COAST UNIVERSITY HOSPITAL | 1 | 0.04 |
| GOODMAYES HOSP | 1 | 0.04 |
| GORDON LESLIE DIAMOND HLTH CARE CTR | 1 | 0.04 |
| GORYEB CHILDRENS HOSP ATLANTIC HLTH | 1 | 0.04 |
| GOTHENBURG UNIV PSYCHIAT OUTPATIENT CLIN | 1 | 0.04 |
| GOVT ISRAEL PINHEIRO HOSP | 1 | 0.04 |
| GPA NET | 1 | 0.04 |
| GREIFSWALD MEDICAL SCHOOL | 1 | 0.04 |
| GRIGORE T POPA UNIVERSITY OF MEDICINE PHARMACY | 1 | 0.04 |
| GRP HLTH RES INST | 1 | 0.04 |
| GRP TECNIMEDE | 1 | 0.04 |
| GUANG ZHOU MED UNIV | 1 | 0.04 |
| GUANGDONG PROV KEY LAB BRAIN FUNCT DIS | 1 | 0.04 |
| GWANG JU TRAUMA CTR | 1 | 0.04 |
| H LEE MOFFITT CANCER CENTER RESEARCH INSTITUTE | 1 | 0.04 |
| H SAN RAFFAELE RESNATI | 1 | 0.04 |
| HADASSAH HEBREO UNIV MED CTR | 1 | 0.04 |
| HAINING MATERNAL CHILD HLTH CARE HOSP | 1 | 0.04 |
| HAKODATE WATANABE HOSP | 1 | 0.04 |
| HAMMONDCARE | 1 | 0.04 |
| HAND MICROSURG CTR EL PASO | 1 | 0.04 |
| HANLLYM UNIV | 1 | 0.04 |
| HARBIN FIRST SPEC HOSP | 1 | 0.04 |
| HARBINE FIRST SPEC HOSP | 1 | 0.04 |
| HARNETT HLTH SYST | 1 | 0.04 |
| HARPER COLL | 1 | 0.04 |
| HARRIS HLTH SYST | 1 | 0.04 |
| HARRY R HORVITZ CTR PALLIAT MED | 1 | 0.04 |
| HARVARD PILGRIM HEALTH CARE | 1 | 0.04 |
| HARVARD TH CHAN SCH PUBL HLTH | 1 | 0.04 |
| HASAN KALYONCU UNIVERSITY | 1 | 0.04 |
| HAYAISHI HOSP OSAKA | 1 | 0.04 |
| HAYWOOD HOSP | 1 | 0.04 |
| HEADACHE MED CTR | 1 | 0.04 |
| HEADACHE TREATMENT CTR | 1 | 0.04 |
| HEALTHMETR OUTCOMES RES LLC | 1 | 0.04 |
| HEART RESEARCH INSTITUTE | 1 | 0.04 |
| HEINRICH HEINE UNIVERSITY DUSSELDORF HOSPITAL | 1 | 0.04 |
| HELIOPOLIS HOSP | 1 | 0.04 |
| HELIOS HOSP STRALSUND | 1 | 0.04 |
| HELIX CLIN RES INC | 1 | 0.04 |
| HELMHOLTZ ASSOCIATION | 1 | 0.04 |
| HELMHOLTZ CENTER MUNICH GERMAN RESEARCH CENTER FOR ENVIRONMENTAL HEALTH | 1 | 0.04 |
| HELSE FONNA HF | 1 | 0.04 |
| HENDRIX COLL | 1 | 0.04 |
| HENRY FORD HOSP HLTH SCI CTR | 1 | 0.04 |
| HERMANAS HOSP | 1 | 0.04 |
| HERMANN HOSP | 1 | 0.04 |
| HIGH PLAINS BAPTIST HOSP | 1 | 0.04 |
| HIGHER TECHNOL EDUC INST EPIRUS | 1 | 0.04 |
| HILLEL YAFFE HOSP | 1 | 0.04 |
| HIROSAKI NATL HOSP | 1 | 0.04 |
| HIROSAKI UNIVERSITY | 1 | 0.04 |
| HIROSHIMA UNIVERSITY | 1 | 0.04 |
| HIV CLIN RES PROGRAM | 1 | 0.04 |
| HLTH CTR OULU | 1 | 0.04 |
| HLTH PSYCHOL CHEM DEPENDENCY | 1 | 0.04 |
| HLTH PUBL SERV MALAGA | 1 | 0.04 |
| HOKKAIDO UNIVERSITY | 1 | 0.04 |
| HOLLY HILL HOSP | 1 | 0.04 |
| HOLOS UNIV GRAD SEMINARY | 1 | 0.04 |
| HOLY CROSS HOSP | 1 | 0.04 |
| HOME HOSP | 1 | 0.04 |
| HONG KONG BAPTIST UNIVERSITY | 1 | 0.04 |
| HOP CORBEIL ESSONNE | 1 | 0.04 |
| HOPITAL UNIVERSITAIRE AMBROISE PARE APHP | 1 | 0.04 |
| HOPITAL UNIVERSITAIRE ARMAND TROUSSEAU APHP | 1 | 0.04 |
| HOPITAL UNIVERSITAIRE BEAUJON APHP | 1 | 0.04 |
| HOPITAL UNIVERSITAIRE EUROPEEN GEORGES POMPIDOU APHP | 1 | 0.04 |
| HOPITAL UNIVERSITAIRE HENRI MONDOR APHP | 1 | 0.04 |
| HOPITAL UNIVERSITAIRE HOTEL DIEU APHP | 1 | 0.04 |
| HOPITAL UNIVERSITAIRE PAUL BROUSSE APHP | 1 | 0.04 |
| HOPITAL UNIVERSITAIRE PITIE SALPETRIERE APHP | 1 | 0.04 |
| HOSHI UNIVERSITY | 1 | 0.04 |
| HOSP ANGELES PEDREGAL | 1 | 0.04 |
| HOSP BERGAMO | 1 | 0.04 |
| HOSP CLIN UNIV VALLADOLID | 1 | 0.04 |
| HOSP DOS DE MAYO | 1 | 0.04 |
| HOSP NERVOUS DIS | 1 | 0.04 |
| HOSP PALLIAT NURSES ASSOC | 1 | 0.04 |
| HOSP PERMAI | 1 | 0.04 |
| HOSP PROCARDIACO | 1 | 0.04 |
| HOSP RAJA PERMAISURI BAINUN | 1 | 0.04 |
| HOSP SAO JOSE | 1 | 0.04 |
| HOSP SISTERS CHAR | 1 | 0.04 |
| HOSP SISTERS CHAR LINZ | 1 | 0.04 |
| HOSP SOCIOSANTARI PERE VIRGILI | 1 | 0.04 |
| HOSP SOCOR | 1 | 0.04 |
| HOSP SON LLATZER | 1 | 0.04 |
| HOSP SPECIAL SURG | 1 | 0.04 |
| HOSP TERRASSA | 1 | 0.04 |
| HOSP UNIV EVANGEL CURITIBA | 1 | 0.04 |
| HOSP VIAREGGIO | 1 | 0.04 |
| HOSPITAL CLINIC UNIVERSITARI DE VALENCIA | 1 | 0.04 |
| HOSPITAL DE CANCER DE BARRETOS | 1 | 0.04 |
| HOSPITAL SANTA MARIA | 1 | 0.04 |
| HOSPITAL SANTA MARIA DELLA MISERICORDIA | 1 | 0.04 |
| HOSPITAL SIRIO LIBANES | 1 | 0.04 |
| HOSPITAL UNIVERSITARI VALL D HEBRON | 1 | 0.04 |
| HOSPITAL UNIVERSITARIO LA PAZ | 1 | 0.04 |
| HOSPITAL UNIVERSITARIO MARQUES DE VALDECILLA HUMV | 1 | 0.04 |
| HOSPITAL UNIVERSITARIO RAMON Y CAJAL | 1 | 0.04 |
| HOUSTON VA HLTH SERV RES DEV CTR EXCELLENCE | 1 | 0.04 |
| HOWARD HUGHES MEDICAL INSTITUTE | 1 | 0.04 |
| HSIN SHENG COLL MED CARE MANAGEMENT | 1 | 0.04 |
| HSR D | 1 | 0.04 |
| HUDDINGE HOSP | 1 | 0.04 |
| HULL ROYAL INFIRM | 1 | 0.04 |
| HUMAINE CLIN SANTA CHIARA | 1 | 0.04 |
| HUNAN PROV HOSP BRAIN DIS | 1 | 0.04 |
| HUNTSMAN CANCER INSTITUTE | 1 | 0.04 |
| HURLEY MED CTR | 1 | 0.04 |
| HYOGO COLLEGE OF MEDICINE | 1 | 0.04 |
| I3 DRUG SAFETY | 1 | 0.04 |
| I3STATPROBE | 1 | 0.04 |
| ICELANDIC HEART ASSOCIATION | 1 | 0.04 |
| ICHILOV SORIASKY GEN HOSP | 1 | 0.04 |
| ICORD | 1 | 0.04 |
| IDC HERMITAGE CAPODIMONTE | 1 | 0.04 |
| IDIBAPS | 1 | 0.04 |
| ILLINOIS INSTITUTE OF TECHNOLOGY | 1 | 0.04 |
| INA CENT HOSP | 1 | 0.04 |
| INAGAKI CLIN | 1 | 0.04 |
| INC RES | 1 | 0.04 |
| INCHEON WORKERS HLTH CTR | 1 | 0.04 |
| INDIANA REGENSTRIEF INST INC | 1 | 0.04 |
| INFECT DIS RES COLLABORAT | 1 | 0.04 |
| INHA UNIVERSITY | 1 | 0.04 |
| INJE UNIVERSITY | 1 | 0.04 |
| INNLANDET HOSPITAL TRUST | 1 | 0.04 |
| INNOVAIT | 1 | 0.04 |
| INNOVAT CLIN RES SICR | 1 | 0.04 |
| INNTALKLIN | 1 | 0.04 |
| INST ASSISTENCIA SANITARIA | 1 | 0.04 |
| INST BEHAV SCI G DE LISIO | 1 | 0.04 |
| INST CANC RES | 1 | 0.04 |
| INST COMMUNITY MED | 1 | 0.04 |
| INST ELECT INFORMAT ENGN IETA | 1 | 0.04 |
| INST INT INTERNET INTERVENT HLTH | 1 | 0.04 |
| INST INVEST BIOMED ST PAU | 1 | 0.04 |
| INST INVEST INNOVAC CIENCIAS BIOMED CADIZ INIBI | 1 | 0.04 |
| INST INVEST SANITARIA GREGORIO MARANON | 1 | 0.04 |
| INST ISRAELITA DE ENSINO PESQUISA ALBERT EINSTE | 1 | 0.04 |
| INST MENTAL HLTH CARE | 1 | 0.04 |
| INST NACL MATERNO PERINATAL | 1 | 0.04 |
| INST NACL NEUROL NEUROCIRUG | 1 | 0.04 |
| INST NEUROL | 1 | 0.04 |
| INST NEUROL INVEST DR RAUL CARREA | 1 | 0.04 |
| INST PHARMACOL CLIN PHARMACOL TOXICOL | 1 | 0.04 |
| INST PREVIDENCIA SERVIDORES ESTADO MINAS GERAIS | 1 | 0.04 |
| INST PSYCHIAT | 1 | 0.04 |
| INST PSYCHIAT FMUSP | 1 | 0.04 |
| INST PSYCHIAT PSYCHOL NEUROSCI IOPPN | 1 | 0.04 |
| INST PSYCHOL | 1 | 0.04 |
| INST RECERCA ST JOAN DEU IRSJD | 1 | 0.04 |
| INST UNIV INVEST ATENCIO PRIMARIA IDIAP | 1 | 0.04 |
| INSTITUT D INVESTIGACIO BIOMEDICA DE BELLVITGE IDIBELL | 1 | 0.04 |
| INSTITUT D INVESTIGACIO BIOMEDICA DE GIRONA IDIBGI | 1 | 0.04 |
| INSTITUT D INVESTIGACIO SANITARIA PERE VIRGILI IISPV | 1 | 0.04 |
| INSTITUTE FOR ANTHROPOLOGICAL RESEARCH ZAGREB | 1 | 0.04 |
| INSTITUTE OF PHYSIOLOGY OF THE CZECH ACADEMY OF SCIENCES | 1 | 0.04 |
| INSTITUTE OF THEORETICAL EXPERIMENTAL BIOPHYSICS | 1 | 0.04 |
| INSTITUTO MEXICANO DEL SEGURO SOCIAL | 1 | 0.04 |
| INSTITUTO POLITECNICO NACIONAL MEXICO | 1 | 0.04 |
| INSTITUTO SUPERIOR DE CIENCIAS DA SAUDE EGAS MONIZ | 1 | 0.04 |
| INT COLLABORAT REPAIR DISCOVERIES | 1 | 0.04 |
| INT SOC CNS DRUG DEV | 1 | 0.04 |
| INTERNATIONAL MAIZE WHEAT IMPROVEMENT CENTER CIMMYT | 1 | 0.04 |
| IQVIA | 1 | 0.04 |
| IRANIAN INST HLTH SCI RES | 1 | 0.04 |
| IRCCS BONINO PULEJO | 1 | 0.04 |
| IRCCS BURLO GAROFOLO | 1 | 0.04 |
| IRCCS FDN | 1 | 0.04 |
| IRCCS FONDAZIONE STELLA MARIS | 1 | 0.04 |
| IRCCS INRCA | 1 | 0.04 |
| IRCCS ISTITUTO ORTOPEDICO GALEAZZI | 1 | 0.04 |
| ISLAMIC AZAD UNIVERSITY | 1 | 0.04 |
| ISRA UNIV | 1 | 0.04 |
| IST DIAG CURA HERMITAGE CAPODIMONTE | 1 | 0.04 |
| ISTANBUL BILGI UNIVERSITY | 1 | 0.04 |
| ISTANBUL GOZTEPE TRAINING AND RESEARCH HOSPITAL | 1 | 0.04 |
| ISTANBUL MEDENIYET UNIVERSITY | 1 | 0.04 |
| ISTANBUL MEDIPOL UNIVERSITY | 1 | 0.04 |
| ISTANBUL METROPOLITAN MUNICIPALITY | 1 | 0.04 |
| ISTANBUL PSYCHIAT INST | 1 | 0.04 |
| ISTANBUL SISLI HAMIDIYE ETFAL TRAINING RESEARCH HOSPITAL | 1 | 0.04 |
| ISTITUTO DI BIOFISICA IBF CNR | 1 | 0.04 |
| ISTITUTO ITALIANO DI TECNOLOGIA IIT | 1 | 0.04 |
| ISTITUTO NANOSCIENZE NANO CNR | 1 | 0.04 |
| IVIC ZULIA | 1 | 0.04 |
| IWATE MEDICAL UNIVERSITY | 1 | 0.04 |
| IZMIR KATIP CELEBI UNIVERSITY | 1 | 0.04 |
| IZMIR TEPECIK TRAINING RESEARCH HOSPITAL | 1 | 0.04 |
| JAGIELLONIAN UNIVERSITY | 1 | 0.04 |
| JAMES A HALEY VETERANS HOSPITAL | 1 | 0.04 |
| JAMES J PETERS VA MEDICAL CENTER | 1 | 0.04 |
| JAMES WHITCOMB RILEY HOSPITAL CHILDREN | 1 | 0.04 |
| JAN VAN BREEMEN RES INST READE | 1 | 0.04 |
| JANSSEN SCI AFFAIRS LLC | 1 | 0.04 |
| JIANAN MENTAL HOSP | 1 | 0.04 |
| JIANGNAN UNIVERSITY | 1 | 0.04 |
| JIANGSU KANION PHARMACEUT CO LTD | 1 | 0.04 |
| JIANGXI PROV KEY LAB AUTON NERVOUS FUNCT DIS | 1 | 0.04 |
| JIANGXI UNIVERSITY OF TRADITIONAL CHINESE MEDICINE | 1 | 0.04 |
| JIKEI UNIVERSITY | 1 | 0.04 |
| JINAN CENTRAL HOSPITAL | 1 | 0.04 |
| JINAN FIRST PEOPLES HOSP | 1 | 0.04 |
| JINDAI CLIN | 1 | 0.04 |
| JINDAI HOSP | 1 | 0.04 |
| JINLING HOSP | 1 | 0.04 |
| JOHN A HARTFORD FDN | 1 | 0.04 |
| JOHN COCHRAN HOSP | 1 | 0.04 |
| JOHN CURTIN SCHOOL OF MEDICAL RESEARCH | 1 | 0.04 |
| JOHN D DINGELL VA MED CTR | 1 | 0.04 |
| JOHN MUIR MED CTR | 1 | 0.04 |
| JOHNS HOPKINS CTR BEHAV HLTH | 1 | 0.04 |
| JOHNSON JOHNSON | 1 | 0.04 |
| JOHNSON JOHNSON USA | 1 | 0.04 |
| JOINT MUNICIPAL AUTHOR WELLBEING RAAHE DIST | 1 | 0.04 |
| JOURNAL WOMENS HLTH | 1 | 0.04 |
| KAFKAS UNIVERSITY | 1 | 0.04 |
| KANEBO LTD | 1 | 0.04 |
| KANGWON NATIONAL UNIVERSITY | 1 | 0.04 |
| KANSAI MEDICAL UNIVERSITY | 1 | 0.04 |
| KANTO ROSAI HOSP | 1 | 0.04 |
| KANTONSSPITAL AARAU AG KSA | 1 | 0.04 |
| KAOHSIUNG ARMED FORCES GEN HOSP | 1 | 0.04 |
| KARAMAN STATE HOSPITAL | 1 | 0.04 |
| KARLSKOGA HOSP | 1 | 0.04 |
| KARLSTAD UNIVERSITY | 1 | 0.04 |
| KAYSERI HLTH EDUC INST | 1 | 0.04 |
| KAYSERI TRAINING RESEARCH HOSPITAL | 1 | 0.04 |
| KAZAN FEDERAL UNIVERSITY | 1 | 0.04 |
| KEI MENTAL CLIN | 1 | 0.04 |
| KENT STATE UNIVERSITY | 1 | 0.04 |
| KERMAN UNIVERSITY OF MEDICAL SCIENCES | 1 | 0.04 |
| KH GOTTLICHER HEILAND | 1 | 0.04 |
| KHON KAEN REG HOSP | 1 | 0.04 |
| KING EDWARD VIII HOSP | 1 | 0.04 |
| KING GEORGE S MEDICAL UNIVERSITY | 1 | 0.04 |
| KINGS COLL LONDON | 1 | 0.04 |
| KIRIKKALE UNIVERSITY | 1 | 0.04 |
| KK WOMEN S CHILDREN S HOSPITAL | 1 | 0.04 |
| KKH HVIDOVRE | 1 | 0.04 |
| KLINIKUM KLAGENFURT | 1 | 0.04 |
| KLINIKUM SAARBRUCKEN GMBH | 1 | 0.04 |
| KNOWLEDGE CTR REHABIL FDN LIMBURG | 1 | 0.04 |
| KOC UNIVERSITY | 1 | 0.04 |
| KOCAELI DERINCE TRAINING RESEARCH HOSPITAL | 1 | 0.04 |
| KOCAELI KARAMURSEL STATE HOSPITAL | 1 | 0.04 |
| KOGA RED CROSS HOSP | 1 | 0.04 |
| KOMMUNE HOSP | 1 | 0.04 |
| KOMMUNE HOSP COPENHAGEN | 1 | 0.04 |
| KONKUK UNIVERSITY | 1 | 0.04 |
| KOOTENAI MED CTR | 1 | 0.04 |
| KOREA INSTITUTE OF SCIENCE TECHNOLOGY | 1 | 0.04 |
| KOSIN UNIV | 1 | 0.04 |
| KUNMING INSTITUTE OF BOTANY CAS | 1 | 0.04 |
| KUNMING UNIVERSITY OF SCIENCE TECHNOLOGY | 1 | 0.04 |
| KURASHIKI MUNICIPAL HOSP | 1 | 0.04 |
| KURUME UNIVERSITY | 1 | 0.04 |
| KWONG WAH HOSPITAL | 1 | 0.04 |
| KYOWA HOSP | 1 | 0.04 |
| KYUNG HEE UNIVERSITY | 1 | 0.04 |
| KYYHKYLA REHABIL CTR | 1 | 0.04 |
| LA SALLE UNIV | 1 | 0.04 |
| LAB CIBA GEIGY | 1 | 0.04 |
| LAHTI CENT HOSP | 1 | 0.04 |
| LANCASHIRE CARE FDN TRUST | 1 | 0.04 |
| LANDES NERVEN KRANKENHAUS VALDUNA | 1 | 0.04 |
| LANDES NERVENKRANKENHAUS VALDUNA | 1 | 0.04 |
| LANDSPITALI NATIONAL UNIVERSITY HOSPITAL | 1 | 0.04 |
| LANSI POHJA HEALTHCARE DIST | 1 | 0.04 |
| LAURENTIAN UNIVERSITY | 1 | 0.04 |
| LAURIERHOADESTHERAPY COM | 1 | 0.04 |
| LEEDS GENERAL INFIRMARY | 1 | 0.04 |
| LEGACY COMMUNITY HLTH CTR | 1 | 0.04 |
| LEGACY COMMUNITY HLTH SERV | 1 | 0.04 |
| LEHMAN COLLEGE CUNY | 1 | 0.04 |
| LEICESTER GENERAL HOSPITAL | 1 | 0.04 |
| LEL EMPLOYMENT PENS FUND | 1 | 0.04 |
| LEUPHANA UNIVERSITY LUNEBURG | 1 | 0.04 |
| LI KA SHING KNOWLEDGE INSTITUTE | 1 | 0.04 |
| LIAQUAT NATL HOSP | 1 | 0.04 |
| LILLEHAMMER COLL | 1 | 0.04 |
| LILLY CO LTD | 1 | 0.04 |
| LILLY DEUTSCHLAND GMBH | 1 | 0.04 |
| LILLY GERMANY | 1 | 0.04 |
| LILLY KOREA | 1 | 0.04 |
| LILLY SPAIN | 1 | 0.04 |
| LILLY SUZHOU PHARMACEUT CO LTD | 1 | 0.04 |
| LILLY USA LLC | 1 | 0.04 |
| LINGNAN UNIVERSITY | 1 | 0.04 |
| LINNAEUS UNIVERSITY | 1 | 0.04 |
| LONDON PSYCHIAT HOSP | 1 | 0.04 |
| LONDON SCHOOL ECONOMICS POLITICAL SCIENCE | 1 | 0.04 |
| LONG BEACH VA MED CTR | 1 | 0.04 |
| LONG ISL STATE VET HOME | 1 | 0.04 |
| LONGITUDINAL AGING STUDY AMSTERDAM | 1 | 0.04 |
| LOUGHBOROUGH UNIV TECHNOL | 1 | 0.04 |
| LOUIS STOKES CLEVELAND VETERANS AFFAIRS MEDICAL CENTER | 1 | 0.04 |
| LOUISE WATSON CONSULTING LTD | 1 | 0.04 |
| LOUISIANA STATE UNIVERSITY HEALTH SCIENCES CENTER NEW ORLEANS | 1 | 0.04 |
| LUNDBECK CORPORATION | 1 | 0.04 |
| LUXEMBOURG INSTITUTE OF HEALTH | 1 | 0.04 |
| LYELL MCEWIN HOSPITAL | 1 | 0.04 |
| MACCABI TEL AVIV FC | 1 | 0.04 |
| MACKAY MEMORIAL HOSPITAL | 1 | 0.04 |
| MAES CLIN | 1 | 0.04 |
| MAHIDOL UNIVERSITY | 1 | 0.04 |
| MAILMAN SCH PUBL HLTH | 1 | 0.04 |
| MAINE MEDICAL CENTER | 1 | 0.04 |
| MAINE SPINE REHABIL | 1 | 0.04 |
| MAKERERE UNIVERSITY | 1 | 0.04 |
| MALARDALEN UNIVERSITY | 1 | 0.04 |
| MALATYA STATE HOSPITAL | 1 | 0.04 |
| MANAKAI O MALAMA INTEGRAT HEALTHCARE GRP REHABI | 1 | 0.04 |
| MARAGHEH UNIV MED SCI | 1 | 0.04 |
| MARCHE POLYTECHNIC UNIVERSITY | 1 | 0.04 |
| MARIST COLL | 1 | 0.04 |
| MARYLAND DEPARTMENT OF HEALTH MENTAL HYGIENE | 1 | 0.04 |
| MASARYK UNIVERSITY BRNO | 1 | 0.04 |
| MATER DEI HOSP | 1 | 0.04 |
| MATER MISERICORDIAE UNIVERSITY HOSPITAL | 1 | 0.04 |
| MATHISON CTR MENTAL HLTH RES EDUC | 1 | 0.04 |
| MATSUMOTO MED CTR | 1 | 0.04 |
| MCCLELLAN MEM VET ADM MED CTR | 1 | 0.04 |
| MCNEAL MEM HOSP | 1 | 0.04 |
| MED SPIRITIST ASSOC AMEUBE | 1 | 0.04 |
| MEDICAL UNIVERSITY GDANSK | 1 | 0.04 |
| MEDICAL UNIVERSITY OF INNSBRUCK | 1 | 0.04 |
| MEDICAL UNIVERSITY OF LUBLIN | 1 | 0.04 |
| MEDICAL UNIVERSITY OF WARSAW | 1 | 0.04 |
| MEDIPLEX GRP | 1 | 0.04 |
| MEIJI SEIKA PHARMA CO LTD | 1 | 0.04 |
| MELBOURNE SHOULDER ELBOW CTR | 1 | 0.04 |
| MEM HOSP | 1 | 0.04 |
| MEMORIAL UNIVERSITY NEWFOUNDLAND | 1 | 0.04 |
| MENDELEYEV UNIVERSITY OF CHEMICAL TECHNOLOGY | 1 | 0.04 |
| MENSANA CLIN | 1 | 0.04 |
| MERCER UNIVERSITY | 1 | 0.04 |
| MERCY HLTH RES | 1 | 0.04 |
| METRO MINNESOTA CCOP | 1 | 0.04 |
| METROPOLITAN GEN HOSP | 1 | 0.04 |
| METROPOLITAN HOSP | 1 | 0.04 |
| MICHAEL E DEBAKEY VA MEDICAL CENTER | 1 | 0.04 |
| MICHAEL E DEBAKEY VAMC | 1 | 0.04 |
| MID MISSOURI MENTAL HLTH CTR | 1 | 0.04 |
| MIDATLANTIC MENTAL ILLNESS RES EDUC CLIN CTR VI | 1 | 0.04 |
| MIDDLE OSTROBOTHNIA CENT HOSP | 1 | 0.04 |
| MIDWEST CTR HEAD PAIN MANAGEMENT | 1 | 0.04 |
| MIE PREFECTURAL COLL NURSING | 1 | 0.04 |
| MIGUEL SERVET UNIVERSITY HOSPITAL | 1 | 0.04 |
| MINASERA ALDAN HOSP | 1 | 0.04 |
| MINIST HLTH | 1 | 0.04 |
| MINNESOTA STATE UNIVERSITY MANKATO | 1 | 0.04 |
| MISSOURI STATEUNIVERS | 1 | 0.04 |
| MODEL SPINAL CORD INJURY CARE SYST SO CALIF | 1 | 0.04 |
| MONDRIAAN | 1 | 0.04 |
| MONELL CHEMICAL SENSES CENTER | 1 | 0.04 |
| MONMOUTH MED CTR | 1 | 0.04 |
| MONTCLAIR STATE UNIVERSITY | 1 | 0.04 |
| MONTGOMERY FAMILY PRACTICE RESIDENCY PROGRAM | 1 | 0.04 |
| MOSCOW INSTITUTE OF PHYSICS TECHNOLOGY | 1 | 0.04 |
| MOST HOLY TRINITY HOSP | 1 | 0.04 |
| MOTHER CHILD HLTH INST SERBIA | 1 | 0.04 |
| MRC | 1 | 0.04 |
| MRC BIOSTATISTICS UNIT | 1 | 0.04 |
| MRC LABORATORY MOLECULAR BIOLOGY | 1 | 0.04 |
| MUGLA SITKI KOCMAN UNIVERSITY | 1 | 0.04 |
| MUNDIPHARMA GMBH | 1 | 0.04 |
| MUNICIPAL HLTH SERV AMSTERDAM | 1 | 0.04 |
| N SHORE HEBREW ACAD HIGH SCH | 1 | 0.04 |
| N TROENDELAG RES INST | 1 | 0.04 |
| NAGASAKI MEM HOSP | 1 | 0.04 |
| NAGASAKI UNIVERSITY | 1 | 0.04 |
| NAJU NATL HOSP | 1 | 0.04 |
| NAMAZI HOSP | 1 | 0.04 |
| NAMIK KEMAL UNIVERSITY | 1 | 0.04 |
| NANCHANG UNIVERSITY | 1 | 0.04 |
| NANJING MIL COMMAND | 1 | 0.04 |
| NANJING UNIVERSITY OF CHINESE MEDICINE | 1 | 0.04 |
| NANKAI UNIVERSITY | 1 | 0.04 |
| NANOAXIS LLC | 1 | 0.04 |
| NANTONG UNIVERSITY | 1 | 0.04 |
| NARA MEDICAL UNIVERSITY | 1 | 0.04 |
| NASA AMES RESEARCH CENTER | 1 | 0.04 |
| NASSAU UNIV | 1 | 0.04 |
| NATIONAL ACADEMY OF SCIENCES UKRAINE | 1 | 0.04 |
| NATIONAL AERONAUTICS SPACE ADMINISTRATION NASA | 1 | 0.04 |
| NATIONAL CANCER CENTER JAPAN | 1 | 0.04 |
| NATIONAL CENTER FOR CARDIOVASCULAR RESEARCH CNIC | 1 | 0.04 |
| NATIONAL CHENGCHI UNIVERSITY | 1 | 0.04 |
| NATIONAL HEALTH RESEARCH INSTITUTES TAIWAN | 1 | 0.04 |
| NATIONAL HOSPITAL NORWAY | 1 | 0.04 |
| NATIONAL INSTITUTE FOR ENVIRONMENTAL STUDIES JAPAN | 1 | 0.04 |
| NATIONAL INSTITUTE OF MENTAL HEALTH NEUROSCIENCES INDIA | 1 | 0.04 |
| NATIONAL INSTITUTE OF ONCOLOGY HUNGARY | 1 | 0.04 |
| NATIONAL JEWISH HEALTH | 1 | 0.04 |
| NATIONAL RESEARCH INSTITUTE OF CHINESE MEDICINE | 1 | 0.04 |
| NATIONAL TAIPEI UNIVERSITY | 1 | 0.04 |
| NATL ADVISORY UNIT AGEING HLTH | 1 | 0.04 |
| NATL CTR SPORT EXERCISE MED | 1 | 0.04 |
| NATL DENT CTR | 1 | 0.04 |
| NATL GRAD INST POLICY STUDIES | 1 | 0.04 |
| NATL HEMOPHILIA FDN | 1 | 0.04 |
| NATL HLTH SERV | 1 | 0.04 |
| NATL HLTH SYST | 1 | 0.04 |
| NATL INST NEUROL NEUROSURG | 1 | 0.04 |
| NATL INST OCCUPAT SAFETY HLTH | 1 | 0.04 |
| NATL INST WORKING LIFE | 1 | 0.04 |
| NATL PUBL HLTH INST | 1 | 0.04 |
| NATL REHABIL HOSP | 1 | 0.04 |
| NATL TAITUNG UNIV | 1 | 0.04 |
| NATURA | 1 | 0.04 |
| NAVAL HEALTH RESEARCH CENTER | 1 | 0.04 |
| NAVAL MED CTR | 1 | 0.04 |
| NECMETTIN ERBAKAN UNIVERSITY | 1 | 0.04 |
| NESS ZIONA MENTAL HLTH CTR | 1 | 0.04 |
| NETHERLANDS INSTITUTE FOR HEALTH SERVICES RESEARCH | 1 | 0.04 |
| NETHERLANDS INSTITUTE FOR NEUROSCIENCE NIN KNAW | 1 | 0.04 |
| NETHERLANDS NATIONAL INSTITUTE FOR PUBLIC HEALTH THE ENVIRONMENT | 1 | 0.04 |
| NEUROL OFF VIENNA | 1 | 0.04 |
| NEUROSCI CAMPUS AMSTERDAM | 1 | 0.04 |
| NEUROSCI PAIN RES INST | 1 | 0.04 |
| NEUROSCIENCE RESEARCH AUSTRALIA | 1 | 0.04 |
| NEUROSURG NEUROL GRP INC | 1 | 0.04 |
| NEW ENGLAND CTR HEADACHE | 1 | 0.04 |
| NEW ENGLAND INST BEHAV MED | 1 | 0.04 |
| NEW ENGLAND INST NEUROL HEADACHE | 1 | 0.04 |
| NEW ZEALAND BRAIN RES INST | 1 | 0.04 |
| NEWBORN CHILD HLTH | 1 | 0.04 |
| NICOLAUS COPERNICUS UNIVERSITY | 1 | 0.04 |
| NIH NATIONAL HEART LUNG BLOOD INSTITUTE NHLBI | 1 | 0.04 |
| NIH NATIONAL INSTITUTE OF DIABETES DIGESTIVE KIDNEY DISEASES NIDDK | 1 | 0.04 |
| NIHON UNIVERSITY | 1 | 0.04 |
| NIIGATA UNIV HLTH WELF | 1 | 0.04 |
| NIIGATA UNIVERSITY | 1 | 0.04 |
| NIKEA GEN HOSP | 1 | 0.04 |
| NINGBO MINGZHOU HOSP | 1 | 0.04 |
| NIZIP STATE HOSPITAL | 1 | 0.04 |
| NO NEW JERSEY CCOP | 1 | 0.04 |
| NORMAN COUSINS CTR | 1 | 0.04 |
| NORTH CAROLINA STATE UNIVERSITY | 1 | 0.04 |
| NORTH DIST HOSP | 1 | 0.04 |
| NORTHEAST OHIO MEDICAL UNIVERSITY NEOMED | 1 | 0.04 |
| NORTHEASTERN UNIVERSITY | 1 | 0.04 |
| NORTHERN ILLINOIS UNIVERSITY | 1 | 0.04 |
| NORTHERN OSTROBOTHNIA HOSP DIST | 1 | 0.04 |
| NORWEGIAN LUTHERAN HOSP | 1 | 0.04 |
| NORWEGIAN SCHOOL OF SPORT SCIENCES | 1 | 0.04 |
| NORWEGIAN UNIVERSITY OF LIFE SCIENCES | 1 | 0.04 |
| NOVA SOUTHEASTERN UNIVERSITY | 1 | 0.04 |
| NOVARTIS | 1 | 0.04 |
| NUMEDICUS LTD | 1 | 0.04 |
| NURSING FAC | 1 | 0.04 |
| NYU LANGONE MEDICAL CENTER | 1 | 0.04 |
| OAKLAND UNIVERSITY | 1 | 0.04 |
| OBRIEN INST PUBL HLTH | 1 | 0.04 |
| ODUNPAZARI COMMUNITY HLTH CTR | 1 | 0.04 |
| OKAYA CITY HOSP | 1 | 0.04 |
| OKAYAMA MED CTR | 1 | 0.04 |
| OKAYAMA UNIVERSITY | 1 | 0.04 |
| OKEHAZAMA HOSP | 1 | 0.04 |
| OLMSTED MEDICAL CENTER | 1 | 0.04 |
| OP POHJOLA GRP | 1 | 0.04 |
| OPEN UNIVERSITY UK | 1 | 0.04 |
| OPTUM | 1 | 0.04 |
| ORDU UNIVERSITY | 1 | 0.04 |
| OREBRO MED CTR HOSP | 1 | 0.04 |
| OREGON RESEARCH INSTITUTE | 1 | 0.04 |
| ORION ANALYT | 1 | 0.04 |
| ORTHOPAED INST CHILDREN | 1 | 0.04 |
| ORTON FDN | 1 | 0.04 |
| ORTON REHABIL CTR LTD | 1 | 0.04 |
| OSAKA CITY UNIVERSITY | 1 | 0.04 |
| OSAKA DENT UNIV | 1 | 0.04 |
| OSAKA YUKIOKA COLL HLTH SCI | 1 | 0.04 |
| OSLO AKERSHUS UNIVERSITY COLLEGE | 1 | 0.04 |
| OSPED FATEBENEFRATELLI | 1 | 0.04 |
| OSPED MAGGIORE | 1 | 0.04 |
| OSPED S MARIA DELLA PIETA | 1 | 0.04 |
| OSTRA SJUKHUSET | 1 | 0.04 |
| OTTO FRIEDRICH UNIV | 1 | 0.04 |
| OZEL TORBALI TIP MERKEZI | 1 | 0.04 |
| PACE UNIVERSITY | 1 | 0.04 |
| PAIN DOCTORS | 1 | 0.04 |
| PAIN MED CTR ZWEIBRUCKEN | 1 | 0.04 |
| PAIN MOTION RES GRP | 1 | 0.04 |
| PAIN RES CTR | 1 | 0.04 |
| PALO ALTO UNIV | 1 | 0.04 |
| PANUM INST | 1 | 0.04 |
| PAPAGEORGIOU HOSPITAL | 1 | 0.04 |
| PARADESSIA GOVT HOSP | 1 | 0.04 |
| PARAISO MED CTR | 1 | 0.04 |
| PARTNERS HEALTHCARE SYSTEM | 1 | 0.04 |
| PAVAHCS | 1 | 0.04 |
| PAYAME NOOR UNIVERSITY | 1 | 0.04 |
| PAYNE WHITNEY PSYCHIATR CLIN | 1 | 0.04 |
| PEKING UNION MEDICAL COLLEGE HOSPITAL | 1 | 0.04 |
| PELV PAIN CLIN | 1 | 0.04 |
| PENINSULA DEANERY | 1 | 0.04 |
| PENTICTON REG HOSP | 1 | 0.04 |
| PERMANENTE MEDICAL GROUPS | 1 | 0.04 |
| PFIZER WRD | 1 | 0.04 |
| PIEDMONT HOSP | 1 | 0.04 |
| PIERRE FABRE MEDICAMENT | 1 | 0.04 |
| PINGMEI GRP | 1 | 0.04 |
| PITUBA HOSP BAHIA | 1 | 0.04 |
| PMR HOSP | 1 | 0.04 |
| POCHON CHA UNIVERSITY | 1 | 0.04 |
| POLARIS HLTH DIRECT | 1 | 0.04 |
| POLICLIN S PIETRO | 1 | 0.04 |
| POLYTECHNIC UNIVERSITY OF CATALONIA | 1 | 0.04 |
| POMERANIAN UNIV SLUPSK | 1 | 0.04 |
| POMPEU FABRA UNIVERSITY | 1 | 0.04 |
| PONIENTE HOSP | 1 | 0.04 |
| PORTLAND CTR IMPROVE VET INVOLVEMENT CARE | 1 | 0.04 |
| PORTLAND VA MED CTR R D99 | 1 | 0.04 |
| PORTSMOUTH NAVAL HOSP | 1 | 0.04 |
| POSIT UNIV | 1 | 0.04 |
| PRESBYTERIAN COLL SCH PHARM | 1 | 0.04 |
| PRIME HLTH CLIN | 1 | 0.04 |
| PRINCE HENRY HOSP | 1 | 0.04 |
| PRINCE OF SONGKLA UNIVERSITY | 1 | 0.04 |
| PRINCESS MARGARET CANCER CENTRE | 1 | 0.04 |
| PRINCESS MARGARET HOSP | 1 | 0.04 |
| PROPHASE LLC | 1 | 0.04 |
| PSYCHIAT CLIN ANAGENISSIS | 1 | 0.04 |
| PSYCHIAT CTR NORTH ZEALAND | 1 | 0.04 |
| PSYCHIAT HOSP MARACAIBO | 1 | 0.04 |
| PSYCHIAT OUT PATIENT CLIN | 1 | 0.04 |
| PSYCHIAT OUTPATIENT CLIN | 1 | 0.04 |
| PSYCHOSOMAT HOSP BAD BRAMSTEDT | 1 | 0.04 |
| PUSAN MERINOL MED CTR | 1 | 0.04 |
| PUSAN NATIONAL UNIVERSITY HOSPITAL | 1 | 0.04 |
| PXL HOGESCH | 1 | 0.04 |
| QAZVIN UNIVERSITY OF MEDICAL SCIENCES QUMS | 1 | 0.04 |
| QUEEN SAVANG VADHANA MEM HOSP | 1 | 0.04 |
| QUEENS UNIVERSITY BELFAST | 1 | 0.04 |
| QUINNIPIAC UNIVERSITY | 1 | 0.04 |
| RABIN MEDICAL CENTER | 1 | 0.04 |
| RADCLIFFE INFIRM | 1 | 0.04 |
| RADFORD UNIV | 1 | 0.04 |
| RANCHO LOS AMIGOS MED CTR | 1 | 0.04 |
| RECEP TAYYIP ERDOGAN UNIVERSITY | 1 | 0.04 |
| RED DEER PRIMARY CARE NETWORK | 1 | 0.04 |
| REDWOOD REG MED GRP | 1 | 0.04 |
| REG HLTH CARE AGCY | 1 | 0.04 |
| REG SPECIALIST HOSP WROCLAW | 1 | 0.04 |
| REGENERON | 1 | 0.04 |
| REHACLIN ZURZACH | 1 | 0.04 |
| RESTOREFX | 1 | 0.04 |
| REVIVE THERAPEUT LTD | 1 | 0.04 |
| REYKJALUNDUR REHABIL CTR | 1 | 0.04 |
| RHEUMAKLIN | 1 | 0.04 |
| RICHARD L ROUDEBUSH DEPT VET AFFAIRS MED CTR | 1 | 0.04 |
| RICHARD L ROUDEBUSH VET ADM MED CTR | 1 | 0.04 |
| RICHARDSONS FAMILY PRACTICE | 1 | 0.04 |
| RICK HANSEN INST | 1 | 0.04 |
| RIJNDAM REHABIL CTR | 1 | 0.04 |
| RINNEKOTI RES CTR | 1 | 0.04 |
| RIVERVIEW HOSP | 1 | 0.04 |
| ROBBINS HEADACHE CLIN | 1 | 0.04 |
| ROEHAMPTON UNIVERSITY | 1 | 0.04 |
| ROESSINGH RES DEV | 1 | 0.04 |
| ROLLINS SCHOOL PUBLIC HEALTH | 1 | 0.04 |
| ROMANIAN ACADEMY OF SCIENCES | 1 | 0.04 |
| ROOSEVELT UNIV | 1 | 0.04 |
| ROSOMOFF PAIN CTR | 1 | 0.04 |
| ROSWELL PARK CANCER INSTITUTE | 1 | 0.04 |
| ROUDEBUSH VA CTR IMPLEMENTING EVIDENCE BASED PRAC | 1 | 0.04 |
| ROUDEBUSH VA MED CTR 11H | 1 | 0.04 |
| ROUDEBUSH VAMC HSR D | 1 | 0.04 |
| ROUDEBUSH VET AAIRS MED CTR 11 H | 1 | 0.04 |
| ROUDEBUSH VET AFFAIRS MED CTR 11 H | 1 | 0.04 |
| ROWAN UNIVERSITY | 1 | 0.04 |
| ROWAN UNIVERSITY SCHOOL OF OSTEOPATHIC MEDICINE | 1 | 0.04 |
| ROYAL BOURNEMOUTH HOSP | 1 | 0.04 |
| ROYAL BRISBANE WOMEN S HOSPITAL | 1 | 0.04 |
| ROYAL EDINBURGH ASSOCIATED HOSP | 1 | 0.04 |
| ROYAL HOSP CHILDREN | 1 | 0.04 |
| ROYAL HOSP SICK CHILDREN | 1 | 0.04 |
| ROYAL INFIRM | 1 | 0.04 |
| ROYAL INFIRMARY OF EDINBURGH | 1 | 0.04 |
| ROYAL LIVERPOOL BROADGREEN UNIVERSITY HOSPITALS NHS TRUST | 1 | 0.04 |
| ROYAL LIVERPOOL UNIVERSITY HOSPITAL | 1 | 0.04 |
| ROYAL MANCHESTER CHILDREN S HOSPITAL | 1 | 0.04 |
| ROYAL NATIONAL HOSPITAL FOR RHEUMATIC DISEASES RNHRD | 1 | 0.04 |
| ROYAL NATIONAL ORTHOPAEDIC HOSPITAL NHS TRUST | 1 | 0.04 |
| ROYAL NETHERLANDS ACADEMY OF ARTS SCIENCES | 1 | 0.04 |
| ROYAL PRESTON HOSPITAL | 1 | 0.04 |
| ROYAL S HANTS HOSP | 1 | 0.04 |
| RURAL INST INCLUSIVE COMMUNITIES | 1 | 0.04 |
| RUZINOV UNIV HOSP | 1 | 0.04 |
| RYERSON UNIVERSITY | 1 | 0.04 |
| S CARELIA CENT HOSP | 1 | 0.04 |
| SAARIKKA PRIMARY CARE PUBL UTIL | 1 | 0.04 |
| SAARPFALZ KREIS | 1 | 0.04 |
| SAINT BARNABAS HOSPITAL | 1 | 0.04 |
| SAINT LUKE S MID AMERICA HEART INSTITUTE | 1 | 0.04 |
| SAINT MARIANNA UNIVERSITY | 1 | 0.04 |
| SAINT MICHAELS HOSPITAL TORONTO | 1 | 0.04 |
| SAKARYA TRAINING RESEARCH HOSPITAL | 1 | 0.04 |
| SALISBURY UNIVERSITY | 1 | 0.04 |
| SAN ELOY HOSP | 1 | 0.04 |
| SAN FRANCISCO GENERAL HOSPITAL MEDICAL CENTER | 1 | 0.04 |
| SAN FRANCISCO HOSP | 1 | 0.04 |
| SAN GIOVANNI BATTISTA HOSP | 1 | 0.04 |
| SAN LUIGI HOSP ORBASSANO | 1 | 0.04 |
| SAN PABLO CEU UNIVERSITY | 1 | 0.04 |
| SAN PAOLO POLO UNIVERSITARIA HOSPITAL | 1 | 0.04 |
| SANDOZ | 1 | 0.04 |
| SANFORD BURNHAM PREBYS MEDICAL DISCOVERY INSTITUTE | 1 | 0.04 |
| SANJAY GANDHI POSTGRADUATE INSTITUTE OF MEDICAL SCIENCES | 1 | 0.04 |
| SANLIURFA TRAINING RESEARCH HOSPITAL | 1 | 0.04 |
| SANT EUGENIO HOSPITAL | 1 | 0.04 |
| SANTA CHIARA HOSPITAL | 1 | 0.04 |
| SANTOS HOSP | 1 | 0.04 |
| SAO CAMILO UNIV | 1 | 0.04 |
| SAPPORO MEDICAL UNIVERSITY | 1 | 0.04 |
| SATAKUNTA HOSP DIST | 1 | 0.04 |
| SCH ORGON PIERO BORRELLI | 1 | 0.04 |
| SCHERING PLOUGH CORPORATION | 1 | 0.04 |
| SCOTTSDALE HEALTHCARE FAMILY MED | 1 | 0.04 |
| SE DIST HOSP LEIPZIG | 1 | 0.04 |
| SE LOUISIANA VET HLTH CARE SYST | 1 | 0.04 |
| SEATTLE PACIFIC UNIV | 1 | 0.04 |
| SEATTLE UNIVERSITY | 1 | 0.04 |
| SEBELAS MARET UNIVERSITY | 1 | 0.04 |
| SEMA HOSPITAL | 1 | 0.04 |
| SENDAI CITY HOSP | 1 | 0.04 |
| SEONAM UNIV | 1 | 0.04 |
| SERV ANDALUZ SALUD | 1 | 0.04 |
| SERV MADRILENO SALAD | 1 | 0.04 |
| SERV MADRILENO SALUD | 1 | 0.04 |
| SEVKET YILMAZ TRAINING RESEARCH HOSPITAL | 1 | 0.04 |
| SHAHID BEHESHTI UNIVERSITY MEDICAL SCIENCES | 1 | 0.04 |
| SHANDONG ACADEMY OF MEDICAL SCIENCES | 1 | 0.04 |
| SHANDONG CTR DIS CONTROL PREVENT | 1 | 0.04 |
| SHANDONG PROVINCIAL HOSPITAL | 1 | 0.04 |
| SHANDONG UNIVERSITY OF TRADITIONAL CHINESE MEDICINE | 1 | 0.04 |
| SHANXI HOSP TRADIT CHINESE MED | 1 | 0.04 |
| SHARP GROSSMONT HOSP | 1 | 0.04 |
| SHARP HLTH CARE | 1 | 0.04 |
| SHATIN HOSP | 1 | 0.04 |
| SHEALY INST COMPREHENS HLTH CARE | 1 | 0.04 |
| SHOWA UNIVERSITY | 1 | 0.04 |
| SIDEVIEW | 1 | 0.04 |
| SIMG | 1 | 0.04 |
| SINAI HEALTH SYSTEM TORONTO | 1 | 0.04 |
| SINGAPORE CLIN RES INST | 1 | 0.04 |
| SINGAPORE GENERAL HOSPITAL | 1 | 0.04 |
| SINT LUCAS ANDREAS HOSPITAL | 1 | 0.04 |
| SKANE UNIVERSITY HOSPITAL | 1 | 0.04 |
| SKOKIE HOSP | 1 | 0.04 |
| SLOVAK ACADEMY OF SCIENCES | 1 | 0.04 |
| SLOVAK MEDICAL UNIVERSITY BRATISLAVA | 1 | 0.04 |
| SMBD JEWISH GEN HOSP | 1 | 0.04 |
| SOCIAL WELF HLTH CTR LEMPAALA | 1 | 0.04 |
| SON DURETA HOSPITAL | 1 | 0.04 |
| SOUTH TEXAS VET HEALTHCARE SYST ALMD | 1 | 0.04 |
| SOUTH TEXAS VET HEATH CARE SYST | 1 | 0.04 |
| SOUTHEAST UNIVERSITY CHINA | 1 | 0.04 |
| SOUTHERN ADELAIDE LOCAL HLTH NETWORK | 1 | 0.04 |
| SOUTHERN CROSS UNIVERSITY | 1 | 0.04 |
| SOUTHERN MEDICAL UNIVERSITY CHINA | 1 | 0.04 |
| SOUTHLAKE REGIONAL HEALTH CENTRE | 1 | 0.04 |
| SPA CTR SAN ANDRES | 1 | 0.04 |
| SPECTRA BIOMED INC | 1 | 0.04 |
| SPECTRUM HEALTHCARE WHITEMAN AFB | 1 | 0.04 |
| SPINAL CORD INJURY QUAL ENHANCEMENT RES INITIAT | 1 | 0.04 |
| SPITAL BERN ZIEGLER | 1 | 0.04 |
| SPOFFORD HALL | 1 | 0.04 |
| SRH GESUNDHEITSZENTRUM BAD WIMPFEN | 1 | 0.04 |
| SRINAKHARINWIROT UNIVERSITY | 1 | 0.04 |
| ST CHRISTOPHERS HOSP CHILDREN | 1 | 0.04 |
| ST JOAN DE DEU SSM | 1 | 0.04 |
| ST JOHN S MEDICAL COLLEGE | 1 | 0.04 |
| ST JOHN S NATIONAL ACADEMY OF HEALTH SCIENCES | 1 | 0.04 |
| ST JOHNS PHYS CLIN | 1 | 0.04 |
| ST JOHNS REHAB HOSP | 1 | 0.04 |
| ST JOSEPHS HOSP | 1 | 0.04 |
| ST JUDE CHILDREN S RESEARCH HOSPITAL | 1 | 0.04 |
| ST LOUIS VA MED CTR | 1 | 0.04 |
| ST MARYS DULUTH CLIN HLTH SYST | 1 | 0.04 |
| ST MARYS HEADQUARTERS | 1 | 0.04 |
| ST MARYS HOSP REHABIL CTR | 1 | 0.04 |
| ST ORSOLA FATEBENEFRATELLI HOSP | 1 | 0.04 |
| ST VINCENTS HOSPITAL SYDNEY | 1 | 0.04 |
| STATE HOSP | 1 | 0.04 |
| STATE KEY LAB NEW TECH CHINESE MED PHARMACEUT PRO | 1 | 0.04 |
| STEPPING HILL HOSP | 1 | 0.04 |
| STOCKHOLM COUNTY COUNCIL | 1 | 0.04 |
| STOCKTON STATE COLL | 1 | 0.04 |
| STRAUB CLIN HOSP | 1 | 0.04 |
| SUBST ABUSE MENTAL HLTH SERV ADM | 1 | 0.04 |
| SUNNAAS REHABIL HOSP | 1 | 0.04 |
| SUNY GENESEO | 1 | 0.04 |
| SUZHOU GUANG JI HOSP | 1 | 0.04 |
| SVYASA | 1 | 0.04 |
| SWAMI VIVEKANANDA YOGA RES FDN | 1 | 0.04 |
| SWANSEA UNIVERSITY | 1 | 0.04 |
| SWEDISH CONVENANT PAIN CTR | 1 | 0.04 |
| SWEDISH NATL SOCIAL INSURANCE HOSP | 1 | 0.04 |
| SWISS PARAPLEGIC RESEARCH | 1 | 0.04 |
| SWISS TROPICAL PUBLIC HEALTH INSTITUTE | 1 | 0.04 |
| SYDNEY ORAL MAXILLOFACIAL SURG | 1 | 0.04 |
| SYKEHUSET I VESTFOLD HF | 1 | 0.04 |
| SYMPTOM MANAGEMENT GRP | 1 | 0.04 |
| SYRACUSE HEMATOL ONCOL CCOP | 1 | 0.04 |
| SZEGED UNIVERSITY | 1 | 0.04 |
| SZENT ISTVAN UNIVERSITY | 1 | 0.04 |
| TABDI BUCA MED CTR | 1 | 0.04 |
| TAICHUNG VETERANS GENERAL HOSPITAL | 1 | 0.04 |
| TAINAN MUNICIPAL HOSP | 1 | 0.04 |
| TAIPEI MEDICAL UNIVERSITY HOSPITAL | 1 | 0.04 |
| TAIPEI TZUCHI HOSP | 1 | 0.04 |
| TAISHAN MEDICAL UNIVERSITY | 1 | 0.04 |
| TAMPA GENERAL HOSPITAL | 1 | 0.04 |
| TANTA UNIVERSITY | 1 | 0.04 |
| TAOYUAN ARMED FORCES GEN HOSP | 1 | 0.04 |
| TAOYUAN PSYCHIAT CTR | 1 | 0.04 |
| TARAS SHEVCHENKO NATIONAL UNIVERSITY KIEV | 1 | 0.04 |
| TATVAN STATE HOSPITAL | 1 | 0.04 |
| TAURANGA HOSP | 1 | 0.04 |
| TEAM INST | 1 | 0.04 |
| TECH KRANKENKASSE | 1 | 0.04 |
| TECHNICAL UNIVERSITY OF BERLIN | 1 | 0.04 |
| TEIKYO UNIVERSITY | 1 | 0.04 |
| TEL HAI ACADEMY COLLEGE | 1 | 0.04 |
| TEVA PHARMACEUT | 1 | 0.04 |
| TEXAS STATE UNIVERSITY SAN MARCOS | 1 | 0.04 |
| TEXAS STATE UNIVERSITY SYSTEM | 1 | 0.04 |
| TEXAS TECH UNIVERSITY HEALTH SCIENCE CENTER | 1 | 0.04 |
| TEXAS TECH UNIVERSITY SYSTEM | 1 | 0.04 |
| THE NEW SCHOOL | 1 | 0.04 |
| THERAPIEZENTRUM WIRBELSAULENERKRANKUNGEN | 1 | 0.04 |
| THIRD MED COLL CPLA | 1 | 0.04 |
| TIANJIN INST ENVIRONM OCCUPAT MED | 1 | 0.04 |
| TIANJIN THIRD CTR HOSP | 1 | 0.04 |
| TOHOKU UNIVERSITY | 1 | 0.04 |
| TOKAT STATE HOSPITAL | 1 | 0.04 |
| TOKYO MEDICAL DENTAL UNIVERSITY TMDU | 1 | 0.04 |
| TOKYO MEDICAL UNIVERSITY | 1 | 0.04 |
| TOKYO METROPOLITAN GOVT | 1 | 0.04 |
| TOKYO METROPOLITAN INSTITUTE OF MEDICAL SCIENCE | 1 | 0.04 |
| TOKYO WOMEN S MEDICAL UNIVERSITY | 1 | 0.04 |
| TORONTO GENERAL HOSPITAL | 1 | 0.04 |
| TOTAL HLTH EVALUAT CTR | 1 | 0.04 |
| TOWNSVILLE HOSP | 1 | 0.04 |
| TOYOTA MEM HOSP | 1 | 0.04 |
| TREATMENT NETWORK | 1 | 0.04 |
| TRENGWEATH MENTAL HLTH UNIT | 1 | 0.04 |
| TROIS CYPRES HOSP | 1 | 0.04 |
| TSINGHUA UNIVERSITY | 1 | 0.04 |
| TSUKUBA MED CTR | 1 | 0.04 |
| TUFT MED CTR | 1 | 0.04 |
| TURGUT OZAL UNIVERSITY | 1 | 0.04 |
| TURNING POINT ALCOHOL DRUG CENTRE AUSTRALIA | 1 | 0.04 |
| TUZLA STATE HOSPITAL | 1 | 0.04 |
| TWEESTEDEN HOSP | 1 | 0.04 |
| TZU CHI UNIVERSITY | 1 | 0.04 |
| UAB | 1 | 0.04 |
| UCB CELLTECH | 1 | 0.04 |
| UIT THE ARCTIC UNIVERSITY OF TROMSO | 1 | 0.04 |
| ULL CARE AS | 1 | 0.04 |
| ULUS HOSP | 1 | 0.04 |
| UMBERTO 1 HOSP VENICE MESTRE | 1 | 0.04 |
| UNIFORMED SERVICES UNIVERSITY OF THE HEALTH SCIENCES USA | 1 | 0.04 |
| UNIT STUDY TREATMENT PAIN ALGOS | 1 | 0.04 |
| UNITED STATES AIR FORCE | 1 | 0.04 |
| UNIV ALFONSO X EL SABIO | 1 | 0.04 |
| UNIV APPL SCI ARTS SOUTHERN SWITZERLAND SUPSI | 1 | 0.04 |
| UNIV CHICAGO MED | 1 | 0.04 |
| UNIV CHICAGO MED BIOL SCI | 1 | 0.04 |
| UNIV COMMUNITY HOSP | 1 | 0.04 |
| UNIV ESTATAL VALLE ECATEPEC | 1 | 0.04 |
| UNIV FED PR | 1 | 0.04 |
| UNIV FRANCISCO VITORIA | 1 | 0.04 |
| UNIV GOIAS | 1 | 0.04 |
| UNIV HLTH SCI CHICAGO MED SCH | 1 | 0.04 |
| UNIV HLTH SCI MED INFORMAT TECHNOL UMIT | 1 | 0.04 |
| UNIV HOSP | 1 | 0.04 |
| UNIV HOSP OSIJEK | 1 | 0.04 |
| UNIV HOSP TRAUMATOL | 1 | 0.04 |
| UNIV ISALUD | 1 | 0.04 |
| UNIV KLIN PSYCHIAT | 1 | 0.04 |
| UNIV MED SCI | 1 | 0.04 |
| UNIV MED SCI SERAFIN RUIZ DE ZARATE VILLA CLARA U | 1 | 0.04 |
| UNIV METROPOLITANA SANTOS UNIMES | 1 | 0.04 |
| UNIV MONTERREY | 1 | 0.04 |
| UNIV NOVARA | 1 | 0.04 |
| UNIV PRISHTINA | 1 | 0.04 |
| UNIV SAN BUENAVENTURA | 1 | 0.04 |
| UNIV SAN DIEGO HLTH | 1 | 0.04 |
| UNIV SCI TECHNOL | 1 | 0.04 |
| UNIV SO INDIANA | 1 | 0.04 |
| UNIV SPORTS MED | 1 | 0.04 |
| UNIV SPORTS MED REHABIL | 1 | 0.04 |
| UNIV SURCOLOMBIANA | 1 | 0.04 |
| UNIV TAMPERE | 1 | 0.04 |
| UNIV TEXAS SCH NURSING | 1 | 0.04 |
| UNIV VECHTA | 1 | 0.04 |
| UNIVERSIDAD AUTONOMA METROPOLITANA MEXICO | 1 | 0.04 |
| UNIVERSIDAD CARLOS III DE MADRID | 1 | 0.04 |
| UNIVERSIDAD COLEGIO MAYOR DE CUNDINAMARCA | 1 | 0.04 |
| UNIVERSIDAD DE CANTABRIA | 1 | 0.04 |
| UNIVERSIDAD DE CASTILLA LA MANCHA | 1 | 0.04 |
| UNIVERSIDAD DE HUELVA | 1 | 0.04 |
| UNIVERSIDAD DE LA FRONTERA | 1 | 0.04 |
| UNIVERSIDAD DE LEON | 1 | 0.04 |
| UNIVERSIDAD MIGUEL HERNANDEZ DE ELCHE | 1 | 0.04 |
| UNIVERSIDAD NACIONAL MAYOR DE SAN MARCOS | 1 | 0.04 |
| UNIVERSIDAD PABLO DE OLAVIDE | 1 | 0.04 |
| UNIVERSIDADE CATOLICA PORTUGUESA | 1 | 0.04 |
| UNIVERSIDADE CIDADE DE SAO PAULO | 1 | 0.04 |
| UNIVERSIDADE DE AVEIRO | 1 | 0.04 |
| UNIVERSIDADE DE CAXIAS DO SUL | 1 | 0.04 |
| UNIVERSIDADE DO ESTADO DE SANTA CATARINA | 1 | 0.04 |
| UNIVERSIDADE DO SUL DE SANTA CATARINA | 1 | 0.04 |
| UNIVERSIDADE DO VALE DO ITAJAI | 1 | 0.04 |
| UNIVERSIDADE FEDERAL DE GOIAS | 1 | 0.04 |
| UNIVERSIDADE FEDERAL DE SANTA CATARINA UFSC | 1 | 0.04 |
| UNIVERSIDADE FEDERAL DO ESTADO DO RIO DE JANEIRO | 1 | 0.04 |
| UNIVERSIDADE FEDERAL DO PAMPA | 1 | 0.04 |
| UNIVERSIDADE FEDERAL DO RIO GRANDE DO NORTE | 1 | 0.04 |
| UNIVERSIDADE FEDERAL DO RIO GRANDE DO SUL | 1 | 0.04 |
| UNIVERSIDADE FEDERAL DO TRIANGULO MINEIRO | 1 | 0.04 |
| UNIVERSIDADE LUTERANA DO BRASIL | 1 | 0.04 |
| UNIVERSIDADE PAULISTA | 1 | 0.04 |
| UNIVERSITA DI MODENA E REGGIO EMILIA | 1 | 0.04 |
| UNIVERSITAT SIEGEN | 1 | 0.04 |
| UNIVERSITE CATHOLIQUE LOUVAIN | 1 | 0.04 |
| UNIVERSITE DE STRASBOURG | 1 | 0.04 |
| UNIVERSITE PARIS EST COMUE | 1 | 0.04 |
| UNIVERSITE PARIS EST CRETEIL VAL DE MARNE UPEC | 1 | 0.04 |
| UNIVERSITES DE STRASBOURG ETABLISSEMENTS ASSOCIES | 1 | 0.04 |
| UNIVERSITETI I PRISHTINES | 1 | 0.04 |
| UNIVERSITI MALAYA | 1 | 0.04 |
| UNIVERSITY COLLEGE CORK | 1 | 0.04 |
| UNIVERSITY COLLEGE HOSPITAL IBADAN | 1 | 0.04 |
| UNIVERSITY HOHENHEIM | 1 | 0.04 |
| UNIVERSITY HOSPITAL ARNAU DE VILANOVA | 1 | 0.04 |
| UNIVERSITY HOSPITAL BRNO | 1 | 0.04 |
| UNIVERSITY HOSPITAL BRUSSELS | 1 | 0.04 |
| UNIVERSITY HOSPITALS OF LEICESTER NHS TRUST | 1 | 0.04 |
| UNIVERSITY NORTH CAROLINA HOSPITAL | 1 | 0.04 |
| UNIVERSITY OF AKRON | 1 | 0.04 |
| UNIVERSITY OF ALASKA ANCHORAGE | 1 | 0.04 |
| UNIVERSITY OF ALASKA SYSTEM | 1 | 0.04 |
| UNIVERSITY OF ARKANSAS FAYETTEVILLE | 1 | 0.04 |
| UNIVERSITY OF ARKANSAS LITTLE ROCK | 1 | 0.04 |
| UNIVERSITY OF BELGRADE | 1 | 0.04 |
| UNIVERSITY OF BREMEN | 1 | 0.04 |
| UNIVERSITY OF BRIGHTON | 1 | 0.04 |
| UNIVERSITY OF BUCKINGHAM | 1 | 0.04 |
| UNIVERSITY OF CALIFORNIA BERKELEY | 1 | 0.04 |
| UNIVERSITY OF CALIFORNIA MERCED | 1 | 0.04 |
| UNIVERSITY OF CALIFORNIA RIVERSIDE | 1 | 0.04 |
| UNIVERSITY OF CAPE TOWN | 1 | 0.04 |
| UNIVERSITY OF COLORADO DENVER | 1 | 0.04 |
| UNIVERSITY OF CRETE | 1 | 0.04 |
| UNIVERSITY OF DEBRECEN | 1 | 0.04 |
| UNIVERSITY OF DENVER | 1 | 0.04 |
| UNIVERSITY OF DEUSTO | 1 | 0.04 |
| UNIVERSITY OF EAST ANGLIA | 1 | 0.04 |
| UNIVERSITY OF EASTERN PIEDMONT AMEDEO AVOGADRO | 1 | 0.04 |
| UNIVERSITY OF FINANCE MANAGEMENT WARSAW | 1 | 0.04 |
| UNIVERSITY OF GAVLE | 1 | 0.04 |
| UNIVERSITY OF HAIFA | 1 | 0.04 |
| UNIVERSITY OF IBADAN | 1 | 0.04 |
| UNIVERSITY OF ILLINOIS PEORIA | 1 | 0.04 |
| UNIVERSITY OF ILLINOIS URBANA CHAMPAIGN | 1 | 0.04 |
| UNIVERSITY OF IOANNINA | 1 | 0.04 |
| UNIVERSITY OF JINAN | 1 | 0.04 |
| UNIVERSITY OF JJ STROSSMAYER OSIJEK | 1 | 0.04 |
| UNIVERSITY OF KOBLENZ LANDAU | 1 | 0.04 |
| UNIVERSITY OF KUALA LUMPUR | 1 | 0.04 |
| UNIVERSITY OF LIMERICK | 1 | 0.04 |
| UNIVERSITY OF LONDON SCHOOL OF PHARMACY | 1 | 0.04 |
| UNIVERSITY OF LUCERNE | 1 | 0.04 |
| UNIVERSITY OF LUXEMBOURG | 1 | 0.04 |
| UNIVERSITY OF MAINE | 1 | 0.04 |
| UNIVERSITY OF MAINE ORONO | 1 | 0.04 |
| UNIVERSITY OF MANNHEIM | 1 | 0.04 |
| UNIVERSITY OF MARYLAND BALTIMORE COUNTY | 1 | 0.04 |
| UNIVERSITY OF MARYLAND COLLEGE PARK | 1 | 0.04 |
| UNIVERSITY OF MASSACHUSETTS BOSTON | 1 | 0.04 |
| UNIVERSITY OF MASSACHUSETTS LOWELL | 1 | 0.04 |
| UNIVERSITY OF MASSACHUSETTS WORCESTER | 1 | 0.04 |
| UNIVERSITY OF MILANO BICOCCA | 1 | 0.04 |
| UNIVERSITY OF MONTANA | 1 | 0.04 |
| UNIVERSITY OF MONTANA SYSTEM | 1 | 0.04 |
| UNIVERSITY OF NAVARRA | 1 | 0.04 |
| UNIVERSITY OF NEWCASTLE | 1 | 0.04 |
| UNIVERSITY OF NORTH CAROLINA CHARLOTTE | 1 | 0.04 |
| UNIVERSITY OF NORTH DAKOTA GRAND FORKS | 1 | 0.04 |
| UNIVERSITY OF NORTH TEXAS HEALTH SCIENCE CENTER | 1 | 0.04 |
| UNIVERSITY OF NOTRE DAME | 1 | 0.04 |
| UNIVERSITY OF NOTRE DAME AUSTRALIA | 1 | 0.04 |
| UNIVERSITY OF NOTTINGHAM | 1 | 0.04 |
| UNIVERSITY OF OKLAHOMA NORMAN | 1 | 0.04 |
| UNIVERSITY OF OKLAHOMA SYSTEM | 1 | 0.04 |
| UNIVERSITY OF PALERMO | 1 | 0.04 |
| UNIVERSITY OF PARIS DESCARTES | 1 | 0.04 |
| UNIVERSITY OF PARIS DIDEROT | 1 | 0.04 |
| UNIVERSITY OF PERUGIA | 1 | 0.04 |
| UNIVERSITY OF QUEBEC MONTREAL | 1 | 0.04 |
| UNIVERSITY OF READING | 1 | 0.04 |
| UNIVERSITY OF RHODE ISLAND | 1 | 0.04 |
| UNIVERSITY OF ROSTOCK | 1 | 0.04 |
| UNIVERSITY OF SALFORD | 1 | 0.04 |
| UNIVERSITY OF SKOVDE | 1 | 0.04 |
| UNIVERSITY OF SOFIA | 1 | 0.04 |
| UNIVERSITY OF SOUTH ALABAMA | 1 | 0.04 |
| UNIVERSITY OF SOUTHERN MAINE | 1 | 0.04 |
| UNIVERSITY OF SOUTHERN MISSISSIPPI | 1 | 0.04 |
| UNIVERSITY OF SURREY | 1 | 0.04 |
| UNIVERSITY OF SUSSEX | 1 | 0.04 |
| UNIVERSITY OF TAMPERE | 1 | 0.04 |
| UNIVERSITY OF TARTU | 1 | 0.04 |
| UNIVERSITY OF TASMANIA | 1 | 0.04 |
| UNIVERSITY OF TEHRAN | 1 | 0.04 |
| UNIVERSITY OF TENNESSEE HEALTH SCIENCE CENTER | 1 | 0.04 |
| UNIVERSITY OF TENNESSEE KNOXVILLE | 1 | 0.04 |
| UNIVERSITY OF TEXAS EL PASO | 1 | 0.04 |
| UNIVERSITY OF TEXAS MEDICAL BRANCH GALVESTON | 1 | 0.04 |
| UNIVERSITY OF THE FREE STATE | 1 | 0.04 |
| UNIVERSITY OF TULSA | 1 | 0.04 |
| UNIVERSITY OF VERMONT MEDICAL CENTER | 1 | 0.04 |
| UNIVERSITY OF VERONA | 1 | 0.04 |
| UNIVERSITY OF WARWICK | 1 | 0.04 |
| UNIVERSITY OF WINCHESTER | 1 | 0.04 |
| UNIVERSITY OF WITWATERSRAND | 1 | 0.04 |
| UNIVERSITY OF WUPPERTAL | 1 | 0.04 |
| UNIVERSITY OF ZARAGOZA | 1 | 0.04 |
| UNIVERSITY TORONTO MISSISSAUGA | 1 | 0.04 |
| UROGENIX | 1 | 0.04 |
| US FOOD DRUG ADMINISTRATION FDA | 1 | 0.04 |
| US MED DIV | 1 | 0.04 |
| UW SCH NURSING | 1 | 0.04 |
| VA CENT CALIF HLTH CARE SYST | 1 | 0.04 |
| VA CONNECTICUT | 1 | 0.04 |
| VA CTR EXCELLENCE STRESS MENTAL HLTH | 1 | 0.04 |
| VA CTR EXCELLENCE STRESS MENTAL HLTH CESAMH | 1 | 0.04 |
| VA CTR IMPLEMENTING EVIDENCE BASED PRACTICE | 1 | 0.04 |
| VA CTR PRACTICE MANAGEMENT OUTCOMES RES | 1 | 0.04 |
| VA HSRD CTR EXCELLENCE IMPLEMENTAT EVIDENCE BASE | 1 | 0.04 |
| VA NEW ENGLAND MENTAL ILLNESS RES EDUC CLIN CTR | 1 | 0.04 |
| VA NORTH TEXAS HEALTH CARE SYSTEM | 1 | 0.04 |
| VA OFF PATIENT CARE | 1 | 0.04 |
| VA PALO ALTO HEALTH CARE SYSTEM | 1 | 0.04 |
| VA PORTLAND HLTH CARE SYST | 1 | 0.04 |
| VA PORTLAND HLTH CARE SYST R D99 | 1 | 0.04 |
| VA SOUTH CENT MENTAL ILLNESS RES EDUC CLIN CTR | 1 | 0.04 |
| VA ST LOUIS HLTH CARE SYST | 1 | 0.04 |
| VA ST LOUIS HLTH CARE SYST JOHN COCHRAN DIV | 1 | 0.04 |
| VA WNY HEALTHCARE SYST | 1 | 0.04 |
| VALI DHEBRON UNIV HOSP | 1 | 0.04 |
| VALL D HEBRON INSTITUT DE RECERCA VHIR | 1 | 0.04 |
| VALLEY HLTH SERV ASSOC | 1 | 0.04 |
| VAMC | 1 | 0.04 |
| VASTERAS CENTRAL HOSPITAL | 1 | 0.04 |
| VEDANTA RES | 1 | 0.04 |
| VET AFFAIRS BOSTON HEALTHCARE SYST | 1 | 0.04 |
| VET AFFAIRS CANADA | 1 | 0.04 |
| VET AFFAIRS SALT LAKE CITY HLTH CARE SYST | 1 | 0.04 |
| VET AFFAIRS WEST LOS ANGELES MED CTR | 1 | 0.04 |
| VETERANS MEDICAL RESEARCH FOUNDATION | 1 | 0.04 |
| VIETNAM ERA TWIN REGISTRY | 1 | 0.04 |
| VILLANOVA COLL NURSING | 1 | 0.04 |
| VILLANOVA UNIVERSITY | 1 | 0.04 |
| VISCONTI MODRONE PSYCHIAT MENTAL HLTH CTR | 1 | 0.04 |
| VITA SALUTE SAN RAFFAELE UNIVERSITY | 1 | 0.04 |
| VITOS CLIN PSYCHIAT GIESSEN MARBURG | 1 | 0.04 |
| VITOS CLIN PSYCHOSOMAT MED GIESSEN MARBURG | 1 | 0.04 |
| VITOS CLIN PSYCHOTHERAPY GIESSEN MARBURG | 1 | 0.04 |
| VRINNEVI HOSP | 1 | 0.04 |
| W PK HOSP | 1 | 0.04 |
| WAIKATO HOSPITAL | 1 | 0.04 |
| WASHINGTON STATE UNIV | 1 | 0.04 |
| WEIDEN HOSP | 1 | 0.04 |
| WEST CLIN | 1 | 0.04 |
| WEST LOS ANGELES VET AFFAIRS MED CTR | 1 | 0.04 |
| WESTERN UNIVERSITY OF HEALTH SCIENCES | 1 | 0.04 |
| WHITE PLAINS MED ASSOCIATES | 1 | 0.04 |
| WICHITA STATE UNIVERSITY | 1 | 0.04 |
| WILLIAM S MIDDLETON MEMORIAL VETERANS HOSPITAL | 1 | 0.04 |
| WINGATE INST PHYS EDUC SPORTS | 1 | 0.04 |
| WINONA STATE UNIVERSITY | 1 | 0.04 |
| WITTEN HERDECKE UNIVERSITY | 1 | 0.04 |
| WOMEN INFANTS HOSPITAL RHODE ISLAND | 1 | 0.04 |
| WOMENS MED GRP IRVINE | 1 | 0.04 |
| WOOSUK UNIV ORIENTAL HOSP | 1 | 0.04 |
| WORLDWIDE CLIN TRIALS | 1 | 0.04 |
| WRIGHT STATE UNIVERSITY DAYTON | 1 | 0.04 |
| WYETH EUROPEAN CLIN RES DEV | 1 | 0.04 |
| WYTHENSHAWE HOSPITAL | 1 | 0.04 |
| WYTHENSHAWE HOSPITAL NHS FOUNDATION TRUST | 1 | 0.04 |
| XUZHOU CENT HOSP | 1 | 0.04 |
| XUZHOU MEDICAL COLLEGE | 1 | 0.04 |
| YANGGU 2 PEOPLES HOSP | 1 | 0.04 |
| YEUNGNAM UNIVERSITY | 1 | 0.04 |
| YILDIRIM BEYAZIT UNIVERSITY | 1 | 0.04 |
| YOKOHAMA MINAMI KYOSAI HOSP | 1 | 0.04 |
| YOUNGDONG UNIV | 1 | 0.04 |
| YUKSEK IHTISAS TRAINING RESEARCH HOSPITAL | 1 | 0.04 |
| YUKSEK IHTISAS UNIVERSITY | 1 | 0.04 |
| ZABLOCKI VA MED CTR | 1 | 0.04 |
| ZHEJIANG PHARMACEUT COLL | 1 | 0.04 |
| ZHEJIANG PROVINCIAL PEOPLE S HOSPITAL | 1 | 0.04 |
| ZHEJIANG XIAOSHAN HOSP | 1 | 0.04 |
